# Synergistic Treatment with Antiretrovirals and Laser Interstitial Thermal ThErapy (STARLITE) for unresectable glioblastoma: A phase 1 study protocol

**DOI:** 10.1371/journal.pone.0328204

**Published:** 2025-08-28

**Authors:** Manav Daftari, Christian K. Ramsoomair, Daniel Aaronson, Deepa Seetharam, Emily A. Andreae, Leonela Wright, Sunwoo Han, Jay Chandar, Victor Lu, Vaidya Govindarajan, Michael E. Ivan, Ricardo J. Komotar, Macarena de la Fuente, Ashish H. Shah

**Affiliations:** 1 Section of Virology and Immunotherapy, Department of Neurosurgery, University of Miami Leonard M. Miller School of Medicine, Miami, Florida, United States of America; 2 Department of Neurosurgery, University of Miami Leonard M. Miller School of Medicine, Miami, Florida, United States of America; 3 Protocol Development Unit, Sylvester Comprehensive Cancer Center, University of Miami Leonard M. Miller School of Medicine, Miami, Florida, United States of America; 4 Biostatistics and Bioinformatics Shared Resource, Sylvester Comprehensive Cancer Center, University of Miami Leonard M. Miller School of Medicine, Miami, Florida, United States of America; 5 Neuro-Oncology Division, University of Miami Leonard M. Miller School of Medicine, Miami, Florida, United States of America; Goethe University Hospital Frankfurt, GERMANY

## Abstract

Glioblastoma (GBM) is the most common form of primary adult brain tumor with a five-year survival of <7%. Standard of care (SoC) involves maximal safe surgical resection with chemoradiation; nevertheless, most patients experience disease progression. Deep intracranial GBM is unresectable, limiting treatment to chemoradiation only. Thus, novel approaches are needed. Laser interstitial thermal therapy (LITT) is a promising minimally invasive procedure using thermal energy for focal necrosis. Another approach is antiretroviral therapy (ART), which was initially explored as a treatment for acquired immunodeficiency syndrome (AIDS)-related malignancies, that has recently shown preclinical promise for GBM. We present the Synergistic Treatment with Antiretrovirals and Laser Interstitial Thermal ThErapy (STARLITE) (NCT06428045) trial, which seeks to synergize LITT with ART to treat unresectable GBM. STARLITE is a single-institution, single-arm, phase 1 study. An ART regimen of abacavir, lamivudine, and ritonavir will be administered followed by SoC adjuvant therapy in patients with newly-diagnosed, unresectable GBM after LITT. The primary objective of the study will be the determination of the maximum tolerated dose (MTD) and/or recommended phase 2 dose (RP2D) of ritonavir as part of the ART regimen. Secondary objectives of the clinical trial will be to estimate progression-free survival (PFS) and overall survival (OS). LITT has been shown to have minimal post-operative complications while improving OS in unresectable GBM. In preclinical GBM models, ART has been shown to reduce cell viability and stemness. STARLITE aims to leverage the ability of LITT to target unresectable GBM foci and transiently open the blood-brain barrier (BBB) to increase the tumoral delivery of ART and augment its anti-tumor effects. We hypothesize this combination will be well-tolerated with minimal adverse effects and improve GBM prognosis.

**Trial Registration:**

This clinical trial is registered on ClinicalTrials.gov (NCT06428045). The authors confirm that all ongoing and related trials for this intervention are registered.

## 1. Introduction

Glioblastoma (GBM) is the most common primary brain malignancy in adults and is thought to arise from neural or oligodendrocyte progenitor cells. Although the pathogenesis of GBM is not fully understood, several hallmark mutations have been reported that drive dysregulation of numerous signaling pathways, including amplification of epidermal growth factor (EGFR), TERT promoter mutations, and chromosome 7/10 copy number alterations [1,2]. Given its intratumoral heterogeneity and variable oncogenic intracellular signaling, treatment strategies for GBM remain limited.

The current standard of care (SoC) therapy for GBM includes maximal safe resection followed by adjuvant chemotherapy and radiation [1–3]. Among these, extent of resection has long been known to correlate with outcomes in GBM, and more recently, extensive surgical resection beyond the regions of contrast-enhancement has been shown to improve survival [2,4]. However, due to pervasive local invasion of GBM in distant brain parenchyma, complete surgical resection of GBM remains difficult.

Among the most difficult to manage tumors are unresectable GBMs that are typically located in eloquent areas that are vital to a patient’s functional status. Current SoC treatment for unresectable GBM includes stereotactic biopsy followed by adjuvant chemoradiation. A biopsy for unresectable GBM allows for a histological and molecular diagnosis, however, no surgical cytoreduction benefit is provided [3,5]. Despite maximal medical management, the prognosis for unresectable GBM remains extremely poor with a 5-year survival rate of <7% [3,6–11]. In an effort to improve these dire outcomes, significant efforts have been made to develop novel approaches to treat unresectable GBM, improve overall survival (OS), and reduce disease progression.

One of the most promising treatments for unresectable GBM is laser interstitial thermal therapy (LITT). LITT utilizes thermal energy to ablate target tissue while preventing damage to the surrounding structure(s), resulting in tumor necrosis within the treatment field [12]. Further, the extent of LITT ablation can be controlled and monitored in real-time using magnetic resonance imaging (MRI) thermometry [12]. Although first described in 1983, LITT has only more recently become increasingly utilized due to advancements in intraoperative MRI techniques, laser probe design, and surgical stereotaxy [13]. The safety and efficacy of LITT for newly-diagnosed GBM has been studied with a meta-analysis by Ivan et al. showing MRI-guided LITT is safe with an overall median survival of 14.2 months, which is similar to SoC therapy for high-grade glioma (HGG) [14]. Particularly for deep-seated GBM, LITT offers a safe and effective method for cytoreduction with minimal disruption to surrounding eloquent brain regions [15]. Not only does LITT allow for cytoreduction of GBM cells, it also transiently opens the blood-brain barrier (BBB), allows innate and adaptive immune cell infiltration, and potentiates the effects of systemic therapies [15–17].

Another novel cancer treatment that has increasingly garnered attention is antiretroviral therapy (ART). ART was initially investigated as a treatment for human immunodeficiency virus (HIV)-associated malignancies [18]. However, to date, ART has been tested in clinical trials and used as an adjuvant cancer therapy in Kaposi’s sarcoma, breast cancer, pancreatic adenocarcinoma, head and neck cancer, and HGGs [19–22]. Protease inhibitors such as ritonavir are not only widely used for HIV but have also been found to have anti-angiogenic as well as anti-tumor effects that are unrelated to any anti-viral mechanism [18]. In a clinical trial involving patients with either progressive or recurrent GBM, ART consisting of a combination of ritonavir and lopinavir was found to be well-tolerated but the activity at the given dose and schedule was modest, and the treatment did not meet the efficacy endpoint [23]. Given the numerous recent publications of ART in preclinical GBM studies that describe pleiotropic inhibitory effects, including suppressed cell proliferation and improved survival, further exploration of ART as an adjunct therapeutic in the clinical care of GBM is warranted [18,24,25].

While ART may present a novel method for treating unresectable GBM, it has poor BBB penetrance and is unlikely to serve as an effective monotherapy as demonstrated in vitro [18,26–28]. We hypothesize that combining ART with LITT will be well-tolerated and may act synergistically to increase efficacy. Here, we present the protocol for the STARLITE phase 1 clinical trial, which assesses the tolerability and preliminary efficacy of LITT and ART for unresectable GBM. This novel clinical trial aims to combine the efficacy of focal and systemic therapies to enhance treatment outcomes for GBM (NCT #: NCT06428045).

## 2. Materials and methods

### 2.1. Study design

The STARLITE trial is a phase 1, single-institution, single-arm study to determine the maximum tolerated dose (MTD) and/or recommended phase 2 dose (RP2D) of ritonavir in combination with a fixed dose of abacavir and lamivudine in patients with newly-diagnosed, unresectable GBM. All patients will be assigned to the treatment group and receive LITT followed by an ART regimen consisting of abacavir, lamivudine, and ritonavir in addition to SoC adjuvant chemoradiation. This study consists of two parts: first, a dose escalation/de-escalation of ritonavir to determine the potential MTD and/or RP2D (part one) using a traditional 3 + 3 design and second, a dose expansion cohort (part two). In total, up to 24 study participants may be enrolled. In the first portion of the study, up to six patients may be enrolled in up to four different dose levels to determine the MTD/RP2D of ritonavir. In the second portion of the study, after the MTD/R2PD dose has been established, up to 18 patients, including the 6 patients from the RP2D cohort from part one, will be enrolled to confirm the safety of the RP2D and assess the preliminary efficacy of the treatment.

**Fig 1:**
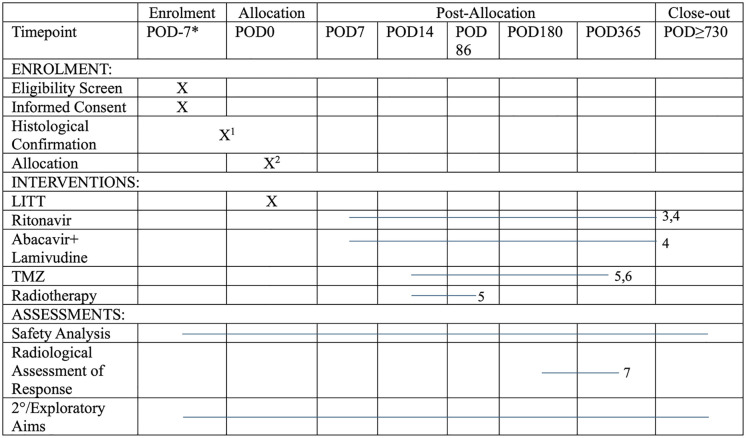
SPIRIT schedule of enrollment, interventions, and assessments for the STARLITE clinical trial. *Abbreviations:* ART, antiretroviral therapy; GBM, glioblastoma; LITT: laser interstitial thermal therapy; MTD, maximum tolerated dose; POD: postoperative day; RP2D, recommended phase 2 dose; TMZ: temozolomide.*Screening assessments are to be conducted up to 7 days before initiating the study, unless otherwise specified. ^1^ Patients will be evaluated by the neurosurgical team prior to surgery and will have either a suspected diagnosis of a GBM or a histologically confirmed GBM at the time of enrollment. If frozen section diagnosis is equivocal or non-diagnostic of glioma, the patient(s) may continue to receive LITT at the surgeon’s discretion and will be deferred for further study participation until the histological review is complete. ^2^ Single-arm study. ^3^ In part 1, the ritonavir dose will be dose escalated/de-escalated using at most four dose levels of ritonavir to determine the potential MTD (or RP2D, if the MTD is not attained). In part 2, ritonavir will be administered at the MTD (or RP2D, if the MTD is not attained) determined from part 1 of the study. ^4^ Patients may receive ART beyond their participation in this study at the discretion of their treating physician. ART may begin later if the participant is considered unable to begin treatment due to their postoperative condition, i.e., inability to swallow medications. ^5^ Adjuvant therapy consisting of radiotherapy and TMZ will be administered to all patients starting on POD14 ± 7 days, provided patients have sufficiently recovered from surgery. ^6^ Following completion of radiotherapy and the 6-week (42-day) regimen of 75 mg/m² TMZ, patients will have a 4-week (±2 week) rest period before starting adjuvant TMZ treatment, which will be administered for six 28-day cycles (168 days total) as follows: Cycle 1 at a dose of 150 mg/m² one time per day on days 1–5 of a 28-day cycle and Cycles 2–6 at a dose of 200 mg/m² one time per day on days 1–5 of a 28-day cycle. Please note that dose and frequency of TMZ administration may be altered at the discretion of the treating neuro-oncologist. ^7^ Radiographic assessments will occur at POD180, POD365, and/or at the timing of the best radiographic response.

Enrollment for the STARLITE clinical trial has not begun though is anticipated to begin in March 2025. Data collection will begin following enrollment of the first patient and is anticipated to conclude in March 2030. Following data collection, analysis will be conducted for the primary and secondary outcomes, and trial results will be published shortly after. A full timeline of enrollment, interventions, and assessments for the STARLITE clinical trial can be found in Fig 1.

### 2.2. Study population

Patients with newly-diagnosed, unresectable GBM who have not received prior treatment, including surgical resection or adjuvant therapy, will be eligible for enrollment. Patients with either a confirmed or presumed case of GBM and for which primary resection is not feasible will initially receive LITT as determined by a group of surgical neuro-oncologists. Investigators will enroll any patients that meet the inclusion criteria (Table 1). A maximum of 24 eligible patients will be included in the study. A summary of the inclusion and exclusion criteria can be found in Table 1.

**Table 1:** Overview of inclusion and exclusion criteria.

Inclusion criteria	Exclusion criteria
Age ≥ 18 years Patients with a histologically confirmed or suspected GBM by MRI In cases with suspected GBM, intraoperative diagnoses of GBM must be made by pathologists. Uni-focal or butterfly gliomas that can receive ≥70% of lesion volume ablated as determined by the treating surgeon Maximal safe resection is not possible or deemed high risk as determined by a group of surgical neuro-oncologists Preoperative Karnofsky performance score ≥ 70 Patients must have demonstrable normal organ function within 14 days of surgery. Patients must be able to understand and sign informed consent.	Received any treatment for GBM, including surgical resection or adjuvant therapy Patients who have received an open biopsy for this disease are still eligible for participation. HLA-B*5701 hypersensitivity Sensitivity to abacavir, lamivudine, or ritonavir Previous history of HIV infection or uncontrolled hepatitis B or C infection Taking dofetilide Regimen of prohibited medications that cannot be discontinued or switched to a more compatible medication Ineligible for MRI with and without contrast Recurrent GBM Current infection Fever within 48 hours of surgery Severe co-morbidity(ies) that increase the risk of surgery, radiation, or chemotherapy, as determined by the treating physician Any co-morbidity, psychiatric ailment, or other situation that in the Investigator’s opinion will prevent administration or completion of protocol therapy Pregnancy Enrollment in other clinical trials

Abbreviations: GBM, glioblastoma; HIV, human immunodeficiency virus; HLA-B*5701, human leukocyte antigen-B variant *5701; IDH, isocitrate dehydrogenase; MRI, magnetic resonance imaging.

### 2.3. Glioblastoma

GBM is defined in this protocol as isocitrate dehydrogenase (IDH) wild-type gliomas including GBM and molecular GBM (low-grade glioma on histology with molecular features of GBM).

### 2.4. Study groups

**Fig 2:**
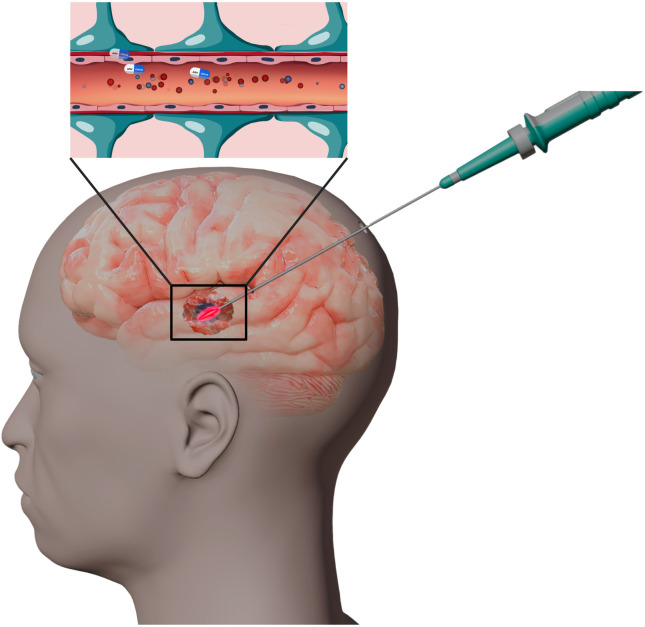
Graphic showing synergy of LITT and ART being evaluated in STARLITE clinical trial. Created in BioRender.com.

Since this is a single-arm study, all patients will receive the same trial intervention of LITT, ART, and SoC adjuvant treatment (temozolomide + radiotherapy) (Fig 2). In part one of STARLITE, up to four ritonavir dose levels will be tested using a 3 + 3 design (Table 2). [29] The expected total number of patients in part one is between 12–18 (if only dose levels 2, 3, and 4 are tested) and maximum 24 (if all four dose levels are tested with 6 patients per level). Once the MTD (or RP2D if the MTD is not attained) has been established, the six patients from the RP2D dose level and up to 18 patients will be enrolled in the dose expansion cohort to confirm the safety and tolerability of ritonavir at the determined RP2D and assess the preliminary efficacy of the treatment.

**Table 2:** STARLITE part 1 dose regimen.

Dose Level	Dosage and Frequency
1	100 mg 2 times daily
2 (Starting Dose)	300 mg 2 times daily
3	400 mg 2 times daily
4	600 mg 2 times daily

### 2.5. Intervention period

**Fig 3:**
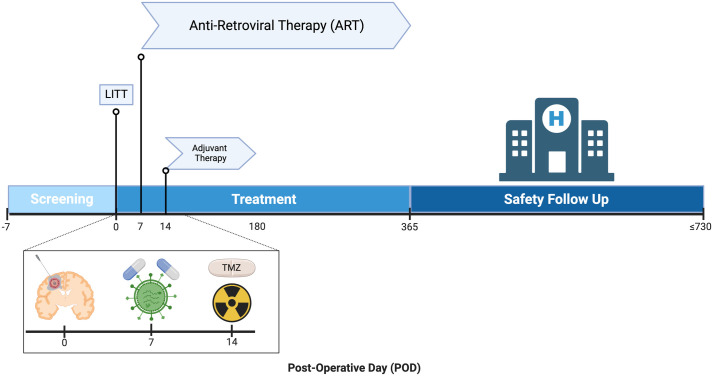
Timeline of STARLITE trial including Screening, Treatment, and Safety Follow-up Periods. Abbreviations: ART, anti-retroviral therapy; LITT, laser interstitial thermal therapy. Created in BioRender.com.

Individual participation is projected to be completed in approximately 24 months, including the screening period before LITT, the Treatment Period, and the Safety Follow-up Period (Fig 3). Patients will be screened up to 7 days before surgery for eligibility into the trial. The full timeline of the STARLITE study can be found in Fig 3. Pre-operatively, patients will be evaluated by the neurosurgical team and will have either a suspected diagnosis of a GBM or a histologically confirmed GBM diagnosis. If the frozen section diagnosis is equivocal or non-diagnostic of glioma, the patient(s) may continue to receive LITT at the surgeon’s discretion and will be deferred for further study participation until the histological review is complete.

#### 2.5.1. MRI-guided LITT.

First, patients will undergo stereotactic needle biopsy before LITT according to institutional standards. Next, laser ablation procedures will be performed using a previously established protocol by institutional neurosurgeons [12]. The degree of laser ablation will be determined by the treating neurosurgeon and the presence of surrounding eloquent structures. Post-operatively, patients will be admitted to the intensive care unit (ICU) and monitored inpatient for 24 hours according to institutional standards.

#### 2.5.2. Antiretroviral regimen.

Patients will be evaluated post-operatively by neuro-oncologists before starting ART. In patients who have sufficiently recovered post-operatively and are negative for human leukocyte antigen-B variant *5701 (HLA-B*5701) hypersensitivity (Table 1), ART will be initiated on postoperative day 7 (POD7).

ART medications will be self-administered by patients and recorded in a monthly medication diary. The ART regimen will consist of abacavir, lamivudine, and ritonavir. The abacavir+lamivudine tablets will be taken orally once daily at a fixed dose of 600 mg abacavir and 300 mg lamivudine. Similarly, ritonavir will be taken orally twice daily; however, the dosage will vary depending on the dose level assignment (Table 2) in part one. Dose escalation and de-escalation will follow the traditional 3 + 3 design. The starting dose of ritonavir will be evaluated at 300 mg twice daily. Dose escalation or de-escalation will depend on the development of dose-limiting toxicities (DLT) over 28 days (4 weeks) following ART administration. The highest dose with no more than 1 out of 6 patients experiencing a DLT will be considered the RP2D. The RP2D will be used in part two in a dose expansion cohort to confirm the safety and tolerability of the RP2D and to assess the preliminary efficacy of the treatment. Patients will receive ART for a maximum of 12 months (1 year) while on study or until disease progression. Please note that patients with a response of stable disease (SD) or better may continue to receive ART beyond the Treatment Period at the Investigator’s discretion.

#### 2.5.3. Adjuvant therapy.

Adjuvant therapy consisting of radiotherapy and temozolomide will be administered to all patients on POD14 ± 7 days, provided patients have sufficiently recovered from surgery. Radiotherapy will be administered for six weeks (42 days). Focal radiotherapy will be administered as per institutional guidelines at a total dose of 60 Gy in 1.8–2.0 Gy fractions (30 total) depending on prognosis and as determined by the treating radiation oncologist with adjuvant temozolomide based on the Stupp protocol [30]. Temozolomide will be administered concurrently with radiotherapy orally over a period of six weeks (42 days) at a dose of 75 mg/m² once daily on a continuous dosing regimen. Following completion of radiotherapy and the 6-week (42-day) regimen of 75 mg/m² temozolomide, participants will have a 4-week (±2 week) rest period before starting adjuvant temozolomide treatment. On POD86 ± 2 days, temozolomide will be administered orally as a maintenance treatment at 150 mg/m² once daily on days 1–5 of a 28-day cycle (Cycle 1) and then at 200 mg/m² once daily on days 1–5 of a 28-day cycle for five cycles (Cycles 2–6).

#### 2.5.4. Follow-up.

Patients will be followed for up to 24 months post-operatively or until patient expiration, whichever occurs later. Initially, patients will be followed throughout one year post-operatively while receiving ART treatment and for ≤1 year post-adjuvant treatment during the 12-month Safety Follow-up Period.

### 2.6. Evaluations

#### 2.6.1. Safety evaluation.

Patients will undergo clinical and laboratory assessments for safety monitoring purposes as described in the Schedule of Assessments of the protocol and be closely monitored for the development of unacceptable toxicity(ies) during the first 28 days (4 weeks) after administration of ART (i.e., the DLT monitoring period). Please note that additional assessments may be ordered at the Investigator’s discretion if clinically indicated.

#### 2.6.2. Disease evaluation.

Disease response will be evaluated using the Response Assessment in Neuro-Oncology (RANO) criteria [31]. Radiographic response assessments will be performed at six months and one year after LITT and/or at the timing of the best radiographic response. If diagnostic imaging results are suggestive of complete response (CR), partial response (PR), or progression, repeat MRI will be performed 4–6 weeks later. If pseudo-progression is considered, short-term steroids are allowed, and a follow-up MRI must confirm progression after 6 weeks. Lesions that decrease in size with steroids on 6-week follow-up MRI will be considered pseudo-progression and will not be considered true progression.

### 2.7. Outcomes

The primary objective of STARLITE is to determine the MTD and/or RP2D of ritonavir in combination with a fixed dose of abacavir and lamivudine in patients with newly-diagnosed, unresectable GBM.

#### 2.7.1. Primary endpoints.

The primary endpoints will be the frequency and severity of adverse events (AEs) and number of DLTs. DLTs will be recorded during the first 28 days (4 weeks) after ART administration and assessed by the National Cancer Institute Common Terminology Criteria for Adverse Events (NCI CTCAE).

#### 2.7.2. Secondary endpoints.

STARLITE’s secondary objectives are to assess the progression-free survival (PFS) and OS for patients with newly-diagnosed, unresectable GBM. PFS is defined as the elapsed time from the start date of LITT to the first documented evidence of disease progression or death from any cause, whichever is earlier. For surviving patients without progression, follow-up will be censored at the date of last documented progression-free status. OS is defined as the elapsed time from the start date of LITT to death from any cause. For surviving patients, follow-up will be censored at the last date known to be alive.

#### 2.7.3. Exploratory aims.

We will determine the preliminary clinical efficacy of ART combined with LITT as measured by the RANO criteria [31]. Exploratory endpoints include the radiographic objective response rate (ORR), which is any CR or PR after LITT, and duration of response (DoR). ORR is defined as the proportion of patients achieving CR or PR. DoR is defined as the elapsed time from date of the first response (CR or PR) to the date of initial objectively documented progression or death from any cause, whichever is earlier. For patients with neither progression nor death, follow-up will be censored at the date of last tumor evaluation.

### 2.8. Statistical methods

Baseline patient characteristics will be summarized using descriptive statistics. Counts and percentages will be used to summarize the distribution of categorical variables while median, range, mean, and standard deviation will be used for continuous variables. Comparisons between groups for categorical outcomes may be performed using Fisher’s exact or chi-square tests, as appropriate. For these analyses, a two-sided p-value < 0.05 will be considered statistically significant. All statistical analyses will be performed by a biostatistician.

#### 2.8.1. Primary outcomes.

Safety data will be analyzed by treatment dose level cohorts and summarized using descriptive statistics to report the number of patients treated, the number of patients who experience DLT, serious adverse events (SAEs), grade 3 or higher AE, the number of patients who discontinue treatment, and the reasons for discontinuation. A patient level summary by worst grade toxicity will be included. The Principal Investigator (PI) will assess toxicity and assign severity and attribution according to the NCI CTCAE. Safety data on all toxicities will be tabulated by type, grade, duration, attribution to treatment, and dose level received.

#### 2.8.2. Secondary and exploratory outcomes.

Secondary and exploratory efficacy endpoints include ORR, PFS, OS, and DoR. The Clopper-Pearson exact method will be used to summarize ORR using point estimates and a corresponding 95% confidence interval (CI). PFS, OS, and DoR will be analyzed using the Kaplan-Meier (KM) method. When the KM method is used, point estimates and 2-sided 95% CIs will be reported for selected times using Greenwood’s variance and the log-log transform method. Median PFS, OS, and DoR will also be reported if attained.

### 2.9. Missed doses

Patients who do not receive protocol therapy dose at the usual required time should be rescheduled as close to the original scheduled date as possible. An exception will be made when rescheduling becomes, in the Investigator’s opinion, medically unnecessary or unsafe because it is too close to the next scheduled evaluation. The missed dose in this case will be abandoned. In the event of toxicity development, the cycle period for ART medications may be decreased from 7 days to 5 days if an alternate cause for toxicity is not found. For patients with a delayed start to adjuvant treatment (i.e., later than two weeks), the timing of follow-up visits will be shifted accordingly, depending on the duration, to accommodate the delay. Other permitted dose holds, modifications, discontinuation, or adjustments to dosing frequency are permitted and described in detail in the protocol.

### 3.0. Ethical considerations

All patients enrolled in the STARLITE clinical trial will provide written informed consent as per Institutional Review Board (IRB) guidelines.

## 3. Discussion

Some challenges in the adjuvant treatment of GBM include drug penetration of the BBB, treatment-resistant GBM stem cells, and the immunosuppressive tumor microenvironment (TME). The BBB, composed of endothelial, ependymal, and tanycytic cells held together by tight junctions, prevents the passage of exogenous substances into the brain parenchyma. As a result, drug delivery to the brain is severely limited due to the inability of drugs to cross the BBB and maintain adequate drug concentrations [32]. Moreover, the GBM TME locally suppresses the host’s innate immune anti-tumor responses, creating a “cold” microenvironment [33].

We hypothesize that the LITT+ART combination treatment approach will be well-tolerated with minimal severe AEs. STARLITE aims to combine the synergistic effects of both LITT and ART to potentiate chemoradiation and improve GBM prognosis. Individually, LITT and ART have shown promising clinical and preclinical results, respectively.

LITT allows for the focal treatment of unresectable GBM in a safe and efficacious manner. In a consecutive case series of 100 patients, Shah et al. demonstrated the safety of LITT with a complication rate of 4% and no permanent complications. Compared with SoC craniotomy for GBM near areas of eloquence, LITT showed a decrease in major complications compared to craniotomy, 5.4% vs. 13.8%, respectively [34]. LITT as a first-line intervention holds tremendous promise due to a decreased postoperative interval to starting SoC medical management compared to resection. The safety and efficacy of LITT for newly-diagnosed GBM has been well-studied with a meta-analysis by Ivan et al. showing MRI-guided LITT was safe with an overall median survival of 14.2 months, which is similar to SoC therapy for HGG [14].

Mechanistically, LITT transiently induces the opening of the BBB. Leuthardt et al. found that opening of the BBB was induced by LITT within 1–2 weeks after the procedure and resolved within 4–6 weeks following the procedure [35]. This transient disruption to the BBB may improve the efficacy of adjuvant therapies for GBM. Lastly, preliminary evidence has suggested that LITT may cause a shift in the TME with increased cluster of differentiation 8 (CD8) T-cell infiltration and programmed death ligand 1 (PD-L1) expression found in post-LITT tumor samples [36].

ART has been well-studied for its anti-tumor properties in prostate cancer, non-small cell lung cancer, and breast cancer by inhibiting tumor cell proliferation, angiogenesis, invasion, and inflammation [18]. Ritonavir was first studied as an adjunct antiretroviral therapy in HIV-positive patients to increase CD4 + lymphocyte counts and reduce the number of HIV ribonucleic acid (RNA) copies and has more recently been studied as a possible treatment for brain malignancies [23,37]. The most common AEs associated with ritonavir therapy include nausea, diarrhea, elevations in hepatic aminotransferases, and elevated cholesterol or triglyceride levels [23,37]. Ritonavir has been studied in patients with progressive or recurrent HGGs as a combination therapy with lopinavir. In this phase 2 clinical trial, patients received combination ritonavir/lopinavir (400 mg/100 mg) orally twice daily. Although the study did not meet its primary objective, the combination therapy was well-tolerated with no grade 3/4 AEs [23]. Given the potential for AEs involving ART and ritonavir, patients in the STARLITE clinical trial will be closely monitored for DLTs.

Preclinical evidence suggests that the anti-tumorigenic effects of ART may also be translatable to GBM [18,26]. ART has been shown to reduce cell viability *in vitro* and reduce stemness in GBM. Rivas et al. showed a decrease in cell viability in glioma cell lines treated with abacavir and lamivudine [26]. Our group has recently demonstrated that HERV-K, a human endogenous retrovirus, sequences are significantly overexpressed in GBM and contribute to the stemness of GBM. We also demonstrated a significant decrease in pluripotency markers OCT4 and Nestin when patient-derived GBM neurospheres were treated with abacavir at 20 μM [27]. Furthermore, using the drug repositioning application DepMap data portal, abacavir has been shown to inhibit tumor cell proliferation in a variety of cancer cell lines, notably in GBM [27]. Additionally, combination therapy of temozolomide, ritonavir, and aprepitant inhibited cell proliferation by 78% [28]. When used alone, ritonavir induces a selective antineoplastic response and exhibits anti-migratory effects. However, when combined with temozolomide, the anti-tumor effects are enhanced, inducing autophagy and increased endoplasmic reticulum stress [25,26]. Given these findings, ART, in combination with SoC adjuvant therapy and the immune modulatory effects of LITT plus the ability of LITT to transiently open the BBB, represents a promising strategy to induce apoptosis in GBM cells and reduce tumor progression.

Given this trial uses a phase 1 single-arm design, some limitations include limited applicability of safety and efficacy results for long-term management of unresectable GBM. Further, because of its focus on the safety and tolerability of ART and LITT combination treatment, our understanding of the therapeutic potential of the individual treatment components will be limited. However, the long-term efficacy of LITT and ART combination therapy will be assessed in follow-up studies.

## 4. Conclusions

Although ART has theoretical potential to treat GBM, the effectiveness of ART medications are currently limited due to challenges in crossing the BBB. LITT, a proven method for treating surgically unresectable GBMs, has the potential to temporarily open the BBB, potentiating the effects of ART against GBM. This protocol outlines the STARLITE clinical trial, which is evaluating the tolerability and preliminary efficacy of combining LITT and ART for unresectable GBM. This innovative trial combines established surgical treatments with ART medications repurposed for GBM with the aim of synergistically acting to improve outcomes for patients with this challenging disease.

### Institutional review board statement

The study was conducted in accordance with the Declaration of Helsinki and approved by the Institutional Review Board of the University of Miami (#20231163, 04/22/2024).

## Supporting information

S1 Protocol 20231163_Shah_ProtocolV2.0b_24Feb2025_Reformatted. (PDF)

S1 Checklist STARLITE SPIRIT CHECKLIST. (PDF)
