# Supplementary material for: Synergistic Treatment with Antiretrovirals and Laser Interstitial Thermal ThErapy (STARLITE) for unresectable glioblastoma: A phase 1 study protocol
Source: PLoS One. 2025 Aug 28;20(8):e0328204. doi: 10.1371/journal.pone.0328204 (PMC12393708; doi:10.1371/journal.pone.0328204)
Supplement: S1 Protocol — (PDF) [file pone.0328204.s001.pdf]

Synergistic Treatment with Antiretrovirals and Laser Interstitial Thermal thErapy (STARLITE) for Unresectable High-Grade Gliomas: A Phase 1 Study

Sponsor Investigator/Principal Investigator	Ashish Harish Shah, MD<br>Department of Neurosurgery<br>University of Miami<br>Sylvester Comprehensive Cancer Center<br>1475 NW 12th Ave<br>Miami, FL 33136
Version	2.0
Version Date	24 February 2025
NCT #	NCT06428045

LIST OF STUDY CONTACTS

**Sponsor-Investigator/Principal Investigator**	Ashish Harish Shah, MD<br>Neurosurgery<br>University of Miami<br>1095 NW 14th Terrace Rm:2-06<br>Miami, FL 33136<br>(305) 243-6946<br>ashah@med.miami.edu
**Co-Principal Investigator**	Macarena de la Fuente, MD<br>Neurology<br>1475 NW 12th Ave floor 2<br>Miami, FL 33136<br>(305) 243-2858<br>MDelaFuente@med.miami.edu
**Biostatistician**	Sunwoo Han, PhD<br>Biostatistics and Bioinformatics Shared Resource (BBSR)<br>Sylvester Comprehensive Cancer Center<br>University of Miami School of Medicine<br>Don Soffer Clinical Research Center<br>1120 NW 14th Street, Room 1054<br>Miami USA-FL 33136-2107<br>Phone: 305-243-0181 (office); (305) 243-2865 (BBSR)<br>Email: sxh3040@med.miami.edu ; bbsr@med.miami.edu
**Protocol Writing**	Protocol Development Office<br>Sylvester Comprehensive Cancer Center<br>University of Miami<br>Phone: (305)243-3379<br>sccc-pdo@med.miami.edu
**Funding Sources**	University of Miami and Medtronic
**Regulatory Sponsor**	University of Miami Sylvester Comprehensive Cancer Center (SCCC)<br>Miami, FL 33136
**Study Intervention**	1. MR-guided Laser Interstitial Thermal Therapy (LITT)<br>2. Antiretroviral therapy (ART) of abacavir+lamivudine and ritonavir<br>3. Adjuvant therapy of temozolomide + radiotherapy
**Supplier for Study Medication(s)**	Not applicable; study medications will be provided from the commercial supply
**IND Status**	IND#: Exempt<br><br>IND Sponsor-Investigator: Ashish Harish Shah, MD

TABLE OF CONTENTS

LIST OF STUDY CONTACTS 2

SIGNATURE PAGE 3

LIST OF ABBREVIATIONS AND DEFINITIONS 7

PROTOCOL SUMMARY 11

SCHEMA 1: DIAGRAM OF STUDY ACTIVITIES 17
SCHEMA 2: DIAGRAM OF RITONAVIR DOSE ESCALATION AND EXPANSION COHORTS 18

1 BACKGROUND 19

1.1 STUDY RATIONALE 19
1.2 BACKGROUND ON GLIOBLASTOMA 19
1.3 BACKGROUND ON MAGNETIC RESONANCE IMAGING-GUIDED LASER INTERSTITIAL THERMAL THERAPY 19
1.4 BACKGROUND ON HUMAN ENDOGENOUS RETROVIRUSES 20
1.5 USE OF ANTIVIRALS AS TREATMENT FOR HIGH-GRADE GLIOMAS 20
1.6 RATIONALE FOR THE COMBINATION OF ANTIVIRAL THERAPY AND MAGNETIC RESONANCE IMAGING-GUIDED LASER INTERSTITIAL THERMAL THERAPY 23
1.7 BENEFIT/RISK ASSESSMENT 23
1.7.1 Assessment of Potential Benefits and Risks 23

2 OBJECTIVES AND ENDPOINTS 25

3 STUDY POPULATION 27

3.1 INCLUSION CRITERIA 27
3.2 EXCLUSION CRITERIA 27
3.3 RECRUITMENT AND ENROLLMENT 28
3.3.1 Facilities for Recruiting and Enrolling Participants 28
3.3.2 Recruitment Methods 28

4 STUDY DESIGN AND TREATMENT PLAN 29

4.1 STUDY DESIGN 29
4.2 STUDY POPULATION 29
4.3 SETTING–DESCRIPTION OF FACILITIES ENROLLING PARTICIPANTS 29
4.4 TREATMENT PLAN 29
4.4.1 Pre- and Perioperative Activities 30
4.4.2 Magnetic Resonance Imaging-guided Laser Interstitial Thermal Therapy 31
4.4.3 Post-operative Care and Monitoring 31
4.4.4 Antiretroviral Therapy (ART) 31
4.4.5 Adjuvant Therapy 31
4.5 DOSING AND ADMINISTRATION 32
4.5.1 Abacavir and Lamivudine 32
4.5.2 Ritonavir 32
4.5.3 MTD of Ritonavir 33
4.5.4 Potential RP2D of Ritonavir 34
4.5.5 Definition of Dose-Limiting Toxicities for Ritonavir 34
4.5.6 Adjuvant Therapy 34
4.6 MISSED DOSES AND ADHERENCE TO DRUG ADMINISTRATION 35
4.7 ACCESS TO STUDY MEDICATIONS AFTER END OF STUDY PARTICIPATION 36
4.8 CONCOMITANT THERAPY 36
4.9 PREMEDICATION 36
4.10 PROHIBITED AND PRECAUTIONARY USE THERAPIES 36

4.10.1 Prohibited Therapies *36*
4.10.2 Precautionary Use Therapies, Herbal Products, Supplements, Food, and Beverages *37*
4.11 CONTRACEPTION 48
4.12 PARTICIPANT DURATION AND FOLLOW-UP 49
4.13 STUDY DURATION 49
4.14 END OF STUDY DEFINITION 49

5 STUDY INTERVENTION DISCONTINUATION AND PARTICIPANT DISCONTINUATION/WITHDRAWAL 50

5.1 DISCONTINUATION OF STUDY INTERVENTION 50
5.2 PARTICIPANT DISCONTINUATION/WITHDRAWAL FROM THE STUDY 50
5.2.1 Data Retention When Participants Withdraw from FDA-Regulated Clinical Trials *50*
5.3 PARTICIPANT REPLACEMENT CRITERIA 50
5.4 SCREEN FAILURES 51
5.5 LOST TO FOLLOW UP 51

6 STUDY AGENTS 52

6.1 ABACAVIR+LAMIVUDINE 52
6.1.1 Description of Product and Mechanism of Action *52*
6.1.2 Formulation *52*
6.1.3 Acquisition and Accountability *52*
6.1.4 Agent Preparation and Administration *52*
6.1.5 Packaging *52*
6.1.6 Labeling *52*
6.1.7 Storage *52*
6.2 RITONAVIR 52
6.2.1 Description of Product and Mechanism of Action *52*
6.2.2 Formulation *53*
6.2.3 Acquisition and Accountability *53*
6.2.4 Agent Preparation and Administration *53*
6.2.5 Packaging *53*
6.2.6 Labeling *53*
6.2.7 Storage *53*
6.3 TEMOZOLOMIDE 53
6.3.1 Description of Product and Mechanism of Action *53*
6.3.2 Formulation *54*
6.3.3 Acquisition and Accountability *54*
6.3.4 Agent Preparation and Administration *54*
6.3.5 Packaging *54*
6.3.6 Labeling *54*
6.3.7 Storage *54*

7 TREATMENT DISCONTINUATION AND ADVERSE EVENT MANAGEMENT 55

7.1 DOSE INTERRUPTION AND DELAYS 55
7.1.1 Antiretroviral Therapy (ART) *55*
7.1.2 Adjuvant Treatment *55*
7.2 DOSE MODIFICATIONS OR DISCONTINUATION 55
7.3 ADVERSE EVENT MONITORING AND MANAGEMENT 57
7.3.1 Antiretroviral Therapy *57*
7.3.2 Laser Interstitial Thermal Therapy *59*
7.3.3 Radiotherapy *59*
7.3.4 Temozolomide *59*

8 SCHEDULE OF ASSESSMENTS 62

9 CORRELATIVE STUDIES ... 65

10 EVALUATION CRITERIA ... 66

10.1 SAFETY ASSESSMENTS ... 66
10.1.1 Medical History ... 66
10.1.2 Concomitant Medications ... 66
10.1.3 Physical Examinations ... 66
10.1.4 Vital Signs ... 66
10.1.5 Performance Status ... 66
10.1.6 Safety Laboratory Analysis ... 66
10.1.7 Imaging ... 68
10.1.8 Electrocardiogram ... 68
10.1.9 Stereotactic Needle Biopsy and Cerebrospinal Fluid Collection ... 68
10.1.10 Adverse Events ... 68
10.2 RESPONSE EVALUATIONS ... 68
10.3 RESPONSE ASSESSMENTS ... 70
10.4 TIME-TO-EVENT ENDPOINTS ... 70
10.4.1 Definitions ... 70
10.4.2 Survival Assessment ... 71

11 ADVERSE EVENTS AND SERIOUS ADVERSE EVENTS ... 72

11.1 DEFINITION OF ADVERSE EVENTS ... 72
11.1.1 Abnormal Laboratory Values ... 72
11.2 DEFINITION OF SERIOUS ADVERSE EVENTS ... 72
11.3 CLASSIFICATION OF AN ADVERSE EVENT ... 73
11.3.1 Severity of Event ... 73
11.3.2 Relationship to Study Intervention ... 73
11.3.3 Expectedness ... 74
11.3.4 Time Period and Frequency for Event Assessment and Follow-Up ... 74
11.4 ADVERSE EVENT REPORTING ... 74
11.5 SERIOUS ADVERSE EVENT REPORTING ... 75
11.6 REPORTING EVENTS TO PARTICIPANTS ... 75
11.7 EVENTS OF SPECIAL INTEREST ... 75
11.8 REPORTING OF PREGNANCY ... 76

12 UNANTICIPATED PROBLEMS ... 77

12.1 DEFINITION OF UNANTICIPATED PROBLEMS ... 77
12.2 UNANTICIPATED PROBLEM REPORTING ... 77
12.3 REPORTING UNANTICIPATED PROBLEMS TO PARTICIPANTS ... 77

13 DATA REPORTING ... 78

13.1 DATA SUBMISSION ... 78
13.2 DATA AND SAFETY MONITORING ... 78

14 STATISTICAL CONSIDERATIONS ... 79

14.1 OVERVIEW ... 79
14.2 SAMPLE SIZE JUSTIFICATION ... 79
14.3 DEFINITIONS AND POPULATIONS FOR ANALYSES ... 79
14.4 STATISTICAL ANALYSES ... 80
14.4.1 Baseline Descriptive Statistics ... 80
14.4.2 Analysis of the Primary Endpoint ... 80
14.4.3 Analysis of the Secondary and Exploratory Endpoint(s) ... 80
14.4.4 Planned Interim Analyses ... 80

14.5 EARLY STOPPING GUIDELINES 80
14.5.1 Early Stopping Due to Safety 80
14.5.2 Early Stopping Due to a Lack of Efficacy 81

15 SUPPORTING DOCUMENTATION AND OPERATIONAL CONSIDERATIONS 82

15.1 INFORMED CONSENT PROCESS 82
15.1.1 Consent/Assent and other Informational Documents Provided to Participants 82
15.1.2 Consent Procedures and Documentation 82
15.2 STUDY DISCONTINUATION AND CLOSURE 82
15.3 CONFIDENTIALITY AND PRIVACY 83
15.4 FUTURE USE OF STORED SPECIMENS AND DATA 84
15.5 STUDY AUDITING AND MONITORING 84
15.5.1 Trial Monitoring, Auditing, and Inspecting 84
15.6 QUALITY ASSURANCE AND QUALITY CONTROL 85
15.7 DATA HANDLING AND RECORD KEEPING 85
15.7.1 Data Collection and Management Responsibilities 85
15.7.2 Study Records Retention 85
15.8 COMPLIANCE WITH PROTOCOL 86
15.9 PUBLICATION AND DATA SHARING 87
15.10 CONFLICT OF INTEREST POLICY 87

16 REFERENCES 88

APPENDIX A: PERFORMANCE STATUS SCALES 90

TABLE OF TABLES

Table 1. Clinical Outcomes by Subtype of Brain Lesion Treated with LITT 19
Table 2. Overall Description of Study Treatment Procedures and Medication Administration 29
Table 3. Dose Levels for Ritonavir 33
Table 4: Established and Other Potentially Significant Drug Interactions 38
Table 5: Safety Criteria for Adjustment or Stopping of Doses of ART and/or TMZ 55
Table 6: Criteria for Response Assessment Incorporating MRI and Clinical Factors 68
Table 7. Early Stopping Rules for Safety in the Dose Expansion Cohort 81

TABLE OF FIGURES

Figure 1: Efficacy of antiretrovirals against patient-derived glioma neurospheres using XTT cell viability assay which is confirmed using pharmacogenetic drug screening approach (depmap.org). 21
Figure 2: Reverse transcriptase inhibitors are efficacious at targeting glioma cells. 22

LIST OF ABBREVIATIONS AND DEFINITIONS

Abbreviation	Definition
AEs	adverse events
ALS	amyotrophic lateral sclerosis
ALT	alanine transaminase
ANC	absolute neutrophil count
ART	antiretroviral therapy
AST	aspartate aminotransferase
AUC	area under the curve
BMP	basic metabolic panel
BUN	blood urea nitrogen
CBC	complete blood count
CFR	Code of Federal Reguations
CIs	confidence intervals
CL_{CR}	creatinine clearance
CMP	comprehensive metabolic panel
CNS	central nervous system
CPK	creatinine phosphokinase
CPT	cell preparation tubes
CR	complete response
CRF	Case Report Form
CRP	C-reactive protein
CSF	cerebrospinal fluid
CTCAE	Common Terminology Criteria for Adverse Events
CYP	cytochrome P450
DHHS	Department of Health and Human Services
DL	dose level
DLTs	dose-limiting toxicities
DMSO	dimethyl sulfoxide
DNA	deoxyribonucleic acid
DNLR	delta neutrophil to lymphocyte ratio
DoR	duration of response
DSMC	University of Miami Sylvester Comprehensive Cancer Center Data and Safety Monitoring Committee
ECG	electrocardiogram
eCRF	electronic Case Report Form
eGFR	estimated glomerular filtration rate

Abbreviation	Definition
EOA	extent of ablation
EOS	End of Study
FDA	Food and Drug Administration
FLAIR	fluid-attenuated inversion recovery
FMF	familial Mediterranean fever
GBM	glioblastoma multiforme
GCSF	granulocyte colony-stimulating factor
GCP	Good Clinical Practice
GD	gadolinium
GGT	gamma-glutamyl transferase
GI	gastrointestinal
Hep B	hepatitis B
Hep C	hepatitis C
HERVs	human endogenous retroviruses
HGG	high-grade gliomas
HIPAA	Health Insurance Portability and Accountability Act of 1996
HIV	human immunodeficiency virus
HLA-B	human leukocyte antigen-B
HLA-B*5701	human leukocyte antigen-B variant *5701
HSRO	Human Subjects Research Office
ICH	International Conference on Harmonisation
ICU	Intensive Care Unit
IDE	Investigational Device Exemption
IND	Investigational New Drug
INR	International Normalized Ratio
IQR	interquartile range
IRB	Institutional Review Board
IUD	intrauterine device
KM	Kaplan-Meier
LDH	lactate dehydrogenase
LFT	Liver Function Tests
LITT	laser interstitial thermal therapy
MI	myocardial infarction
MR	magnetic resonance
MRI	magnetic resonance imaging
MS	multiple sclerosis
MTD	maximum tolerated dose
MTTP	microsomal triglyceride transfer protein

Abbreviation	Definition
N/A; NA	not applicable
NCI	National Cancer Institute
nGBM	newly-diagnosed GBM
NLR	neutrophil to lymphocyte ratio
OHRP	Office for Human Research Protections
ORR	objective response rate
OS	overall survival
PAH	pulmonary arterial hypertension
PBMCs	peripheral blood mononuclear cells
PCP	*Pneumocystis* pneumonia
PCR	polymerase chain reaction
PD	progressive disease
P-gp	permeability glycoprotein
PHI	protected health information
POD	post-operative day
PR	partial response
PT	prothrombin time
PTT	partial thromboplastin time
QA	Quality Assurance
RANO	Response Assessment in Neuro-Oncology
rGBM	recurrent glioblastoma
RN	radiation necrosis
RNA	ribonucleic acid
RP2D	recommended phase 2 dose
RT	radiotherapy
RTV	ritonavir
SAEs	serious adverse events
SCCC	Sylvester Comprehensive Cancer Center
SD	stable disease
SJS	Stevens-Johnson syndrome
snRNA	single-nucleus ribonucleic acid
SSRIs	selective serotonin reuptake inhibitors
T1	T1-Weighted magnetic resonance imaging
T2	T2-Weighted magnetic resonance imaging
T3	triiodothyronine
T4	free thyroxine
TEN	toxic epidermal necrolysis

Abbreviation	Definition
TMZ	temozolomide
TRAEs	treatment-related adverse events
TSH	thyroid-stimulating hormone
TTR	time to recurrence
ULN	upper limit of normal
UM	University of Miami
UPs	unanticipated problems
USPI	United States Package Insert
VA	Veteran's Affairs
WOCBP	women of childbearing potential

PROTOCOL SUMMARY

<table>
<tr><td>Title</td><td colspan="2">Synergistic Treatment with Antiretrovirals and Laser Interstitial Thermal thErapy (STARLITE) for Unresectable High-Grade Gliomas: A Phase 1 Study</td></tr>
<tr><td>Background/Rationale</td><td colspan="2">Outcomes for glioblastoma remain poor despite maximal surgical resection and chemoradiation. To improve outcomes for this devastating disease, we will be synergistically targeting glioblastoma using minimally invasive laser interstitial thermal therapy (LITT) and antiretroviral therapy (ART) to potentiate chemoradiation. This will be a first-in-human study assessing the effects of LITT and ART for newly-diagnosed high-grade glioma (HGG).</td></tr>
<tr><td></td><td>Objectives</td><td>Endpoints</td></tr>
<tr><td>Primary</td><td></td><td></td></tr>
<tr><td rowspan="2"></td><td rowspan="2">To determine maximum tolerated dose (MTD) and/or recommended phase 2 dose (RP2D) of ritonavir in combination of fixed dose of abacavir and lamivudine in patients with newly-diagnosed, unresectable HGG.</td><td>Frequency and severity of AEs. For dose-limiting toxicity (DLT) as per National Cancer Institute Common Terminology Criteria for Adverse Events version 5.0 (NCI CTCAE v5.0), number of DLTs during the first 28 days (4 weeks) after the administration of ART.</td></tr>
<tr><td>DLT, assessed by NCI CTCAE v5.0, is defined as the manifestation of one or more of the following treatment-related adverse events (TEAEs) during the first 28 days (4 weeks) after administration of ART:
• Hematologic Events
o Grade 4 neutropenia lasting ≥ 7 days (granulocyte colony-stimulating factor [GCSF] use allowed)
o Grade 4 anemia
o Grade 4 thrombocytopenia, grade 3 if associated with bleeding
o Febrile neutropenia
• Non-hematologic ≥Grade 4 toxicity
o Hepatic impairment
• Uncontrolled diarrhea that is unresponsive to supportive care, medications, and dietary changes
• Wound dehiscence (≥Grade 3)
• Symptomatic cerebral edema with worsening midline shift > 10 mm (≥Grade 4) with neurological deficits
• Symptomatic intracranial hemorrhage (≥Grade 4)</td></tr>
</table>

		• Any TRAE that leads to >25% of the doses missed during the first 4 weeks
**Secondary**		
	To estimate progression-free survival (PFS) and overall survival (OS) for patients with newly-diagnosed, unresectable HGG.	Progression-free survival (PFS) is defined as the elapsed time from start date of LITT to first documented evidence of disease progression or death from any cause, whichever is earlier. For surviving patients without progression, follow-up will be censored at the date of last documented progression-free status.
		Overall survival (OS) defined as the elapsed time from start date of LITT to death from any cause. For surviving patients, follow-up will be censored at the last date known to be alive.
**Exploratory**		
	To assess preliminary clinical efficacy of ART+LITT as measured by the Response Assessment in Neuro-Oncology (RANO) criteria, in terms of radiographic objective response rate (ORR=complete response [CR]+partial response [PR]) and duration of radiographic response (DoR).	Radiographic objective response rate (ORR; complete response [CR] or partial response [PR]) after LITT as determined by the RANO criteria
		Duration of response (DoR) to treatment by the RANO criteria
Study Intervention	1. MR-guided Laser Interstitial Thermal Therapy (LITT)<br>2. Antiretroviral therapy (ART) of abacavir+lamivudine and ritonavir<br>3. Adjuvant therapy of temozolomide + radiotherapy	
Indication	Patients with newly-diagnosed, unresectable HGG	
Phase	1	
Study Design	This study will be a single institution, single-arm, phase 1 study to determine the RP2D of ritonavir as part of the ART regimen of abacavir, lamivudine, and ritonavir followed by standard of care adjuvant therapy (temozolomide + radiotherapy) in patients with newly-diagnosed, unresectable HGG post LITT. The study will consist of two parts: dose escalation/de-escalation of ritonavir (Part 1) with a traditional 3+3 design and dose expansion (Part 2).	

Treatment Plan	Enrolled participants will receive study treatment as described below: **Overall Description of Study Treatment Procedures and Medication Administration**

**Study Procedure or Medication**	**POD0**	**POD7***	**POD14 ±7 days**	**POD57 ±3 days**	**POD86 ±2 days**	**POD255 ±3 days to POD365 ±2 days or until disease progression****
**LITT**	Single-stage procedure following stereotactic needle biopsy	Not performed	Not performed	Not performed	Not performed	Not performed
**ART†**						
**Abacavir + Lamivudine**	Not administered	1 tablet consisting of 600 mg of abacavir and 300 mg of lamivudine 1 time daily	1 tablet consisting of 600 mg of abacavir and 300 mg of lamivudine 1 time daily	1 tablet consisting of 600 mg of abacavir and 300 mg of lamivudine 1 time daily	1 tablet consisting of 600 mg of abacavir and 300 mg of lamivudine 1 time daily	1 tablet consisting of 600 mg of abacavir and 300 mg of lamivudine 1 time daily
**Ritonavir**	Not administered	2 times daily; Dose assignment per 3+3 design	2 times daily; Dose assignment per 3+3 design	2 times daily; Dose assignment per 3+3 design	2 times daily; Dose assignment per 3+3 design	2 times daily; Dose assignment per 3+3 design
**Adjuvant Therapy**						
**RT**	Not administered	Not administered	Focal RT administered for 6 weeks (42 days) on a Monday through Friday schedule	Not administered	Not administered	Not administered
**TMZ**	Not administered	Not administered	Administered at 75 mg/m² orally 1 time daily on a continuous schedule (including weekends and holidays)	Start of a 4-week (±2 week) rest period prior to start of adjuvant TMZ treatment.	Adjuvant Therapy: Cycle 1: 150 mg/m² orally 1 time daily on days 1–5 of a 28-day cycle. Cycles 2 to 6: 200 mg/m² orally 1 time	Not administered

<table>
<tr><td></td><td>
<table>
<tr><td></td><td></td><td></td><td>for 6 weeks (42 days)</td><td></td><td>daily on days 1–5 of a 28-day cycle</td><td></td></tr>
</table>

Abbreviations: ART, antiretroviral therapy; LITT, laser interstitial thermal therapy; POD, postoperative day; RT, radiotherapy; TMZ, temozolomide
* Antiretroviral therapy (ART) may begin later if the participant is considered unable to begin treatment due to their postoperative condition, ie, inability to swallow medications.
** Participants with a response of stable disease (SD) or better may continue to receive ART beyond the Study Treatment Period at the Investigator’s discretion.
† Participants with a pending human leukocyte antigen-B (HLA-B) test result may still undergo the LITT procedure as part of their standard medical care. However, the HLA-B test result must be obtained prior to a participant starting ART as part of this study. Participants with the human leukocyte antigen-B variant *5701 (HLA-B*5701) must be excluded from study participation; however, these participants will continue to receive standard of care treatment, which includes radiotherapy and chemotherapy, as per the treating neuro-oncologist.

The study will be conducted in two parts as follows:

- **Part 1: Dose escalation/de-escalation of ritonavir** in the ART regimen will be conducted using at most four dose levels of ritonavir to determine the potential MTD (or RP2D, if the MTD is not attained).
- **Part 2: As dose expansion cohort,** an additional 6–18 participants including 6 participants treated with RP2D in part 1 will be enrolled to confirm the safety and tolerability of the RP2D of ritonavir in the ART regimen and to assess the preliminary efficacy of the treatment.
</td></tr>
<tr><td>Correlative Studies</td><td>Blood samples, cerebrospinal fluid (CSF) samples, and solid tumor specimens will be collected for correlative studies.</td></tr>
<tr><td>Number of Patients/Target Study Population</td><td>Investigators will enroll any patient with either a confirmed or presumed case of HGG that has not received a surgical resection or adjuvant treatment. LITT will be offered to patients who are not amenable to surgical resection as determined by a group of surgical neuro-oncologists. A total sample size of 24 eligible patients will be enrolled on this study.</td></tr>
<tr><td>Inclusion/Exclusion Criteria</td><td>

Inclusion Criteria

1. Age ≥ 18 years.
2. Patients with a histologically confirmed or suspected HGG by magnetic resonance imaging (MRI).
    a. For cases with suspected HGG, intraoperative frozen section diagnoses of HGG must be made by pathologists.
    b. HGG for this study include IDH wild-type gliomas including glioblastoma and molecular GBM (low-grade glioma on histology with molecular features of GBM).
3. Uni-focal or butterfly gliomas that can receive ≥70% of lesion volume ablated as determined by the treating surgeon.
4. Gliomas must be located or positioned where surgical resection is either not feasible or high-risk as deemed by a group of surgical neuro-oncologists.
5. Preoperative Karnofsky score ≥ 70.
6. Patients must have demonstrable normal organ function as defined below within 14 days of surgery.
    a. Absolute neutrophil count (ANC) ≥ 1500 cells/mm^3
</td></tr>
</table>

	b. Platelets ≥ 100,000 cells/mm^3<br>c. Hemoglobin ≥ 9.0 g/dL. Use of transfusion or other intervention to achieve this hemoglobin level is acceptable.<br>d. Blood urea nitrogen (BUN) ≤ 35 mg/dL and creatinine ≤ 1.9 mg/dL and estimated glomerular filtration rate (eGFR) or creatinine clearance rate > 50 mL per minute.<br>e. Electrocardiogram (ECG) without evidence of acute cardiac ischemia.<br>f. Prothrombin time (PT)/International Normalized Ratio (INR) <1.4<br>g. Liver function tests: Aspartate aminotransferase (AST) and alanine transaminase (ALT) at or below 2.5 times the upper limit of normal (ULN).<br>h. Sodium level > 130 mg/L. Use of salt resection or hypertonic saline to achieve this sodium level is acceptable.<br>7. Patients must be able to understand and sign informed consent.<br><br>**Exclusion Criteria**<br>1. Patients with HLA-B*5701 hypersensitivity. NOTE: patients with a pending HLA-B test result may still undergo the LITT procedure as part of their standard medical care. However, the HLA-B test result must be obtained prior to a participant starting ART as part of this study.<br>2. Patients with sensitivity to abacavir, lamivudine, or ritonavir.<br>3. Patients with a previous history of human immunodeficiency virus (HIV) infection.<br>4. Patients with uncontrolled hepatitis B or C infection.<br>5. Patients who have received any surgical resection for this tumor.<br>a. Patients who have received an open biopsy for this disease are still eligible for participation.<br>6. Patients who have received chemotherapy or radiation for this disease.<br>7. Patients who are taking dofetilide.<br>8. Patients on a regimen of 1 or more prohibited medications that cannot be discontinued or switched to a more compatible medication.<br>9. Patients not eligible to obtain MRI with and without contrast.<br>10. Recurrent HGG.<br>11. Presence of current infection, such as sepsis, meningitis, bacteremia, or pneumonia.<br>12. Fever within 48 hours of surgery (Temperature> 38.0°C).<br>13. Severe co-morbidity that would confer excess risk of surgery, radiation, or chemotherapy, as determined by the treating physician.<br>14. Any co-morbidity or psychiatric ailment that in the Investigator's opinion will prevent administration or completion of protocol therapy.<br>15. Pregnant women.<br>16. Patients must be willing to use contraception.<br>17. Patients receiving other investigational agents or concurrent enrollment in another therapeutic clinical trial.<br>18. Prisoners.<br>19. Adults unable to consent.
Description of Facilities Enrolling Participants	Study personnel will identify, recruit, enroll and treat patients by Investigators at the University of Miami Sylvester Comprehensive Cancer Center (SCCC) inclusive of all satellite sites. Sylvester Comprehensive Cancer Center (SCCC) will be the only study site.

Study Duration	Participation in the study will last approximately 24 months (2 years). This includes a Screening Period of up to 7 days before LITT, a Treatment Period of up to 12 months (1 year), and a Safety Follow-Up Period of up to 12 months (1 year) or until participant expiration, whichever occurs later. Participants that respond to study treatment may continue to receive ART at the Investigator's discretion.
Expected Study Duration	With a duration of enrollment of 36 months (3 years) and a per-patient study participation of approximately 24 months (2 years), the total estimated study duration will be approximately 5 years.
Statistical Analysis	For dose escalation/de-escalation of ritonavir, a traditional 3+3 design will be performed for testing four planned dose levels: 100, 300 (starting dose), 400, and 600 mg two times per day. Dose expansion will be conducted to confirm safety and tolerability of the determined RP2D from the dose-finding part and to assess the preliminary efficacy of the treatment.<br><br>Based on this 3+3 design, the number of participants for dose escalation/de-escalation is between 12–18 (if only dose levels 2, 3, and 4 are tested) and maximum 24 (if all four dose levels are tested with 6 participants per level). Thus, the expected total number of participants in part 1 is 12–24. An additional 6–18 participants including 6 participants treated with the RP2D in part 1 will be enrolled in dose-expansion cohort to be treated at the RP2D of ritonavir.

Schema 1: Diagram of Study Activities

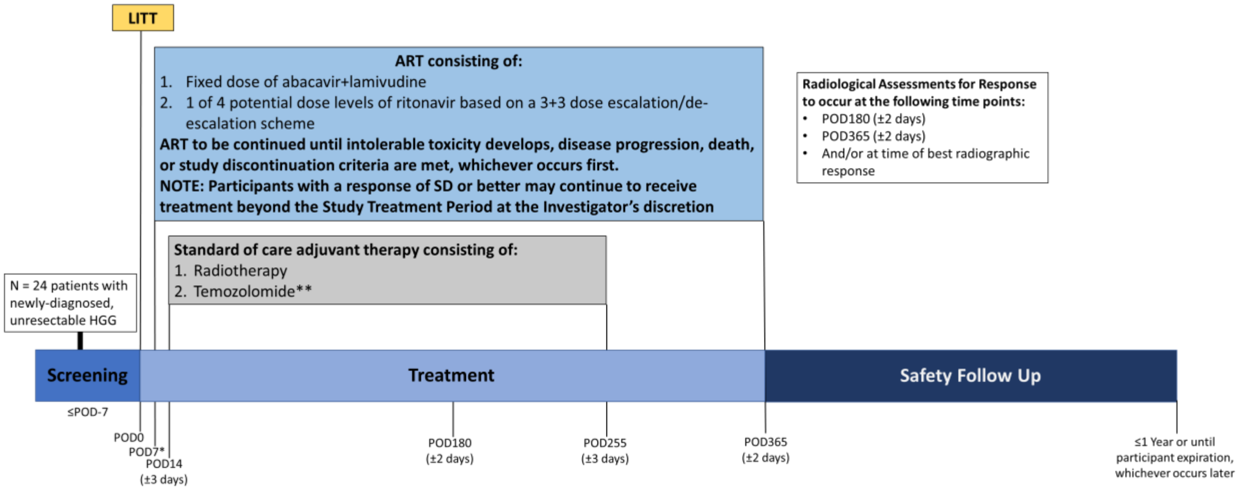

* Antiretroviral therapy (ART) may begin later if the participant is considered unable to begin treatment due to their postoperative condition, ie, inability to swallow medications.

** In accordance with standard of care, temozolomide will be administered concurrently with radiotherapy over a period of 6 weeks (42 days). Please note that radiotherapy will be administered on a Monday through Friday schedule while temozolomide will be administered at 75 mg/m² orally 1 time daily on a continuous schedule (including weekends and holidays). Following completion of radiotherapy and 75 mg/m² temozolomide, participants will have a 4-week (±2 week) rest period prior to starting adjuvant temozolomide treatment for 6 28-day cycles:

- Cycle 1: 150 mg/m² orally 1 time daily on days 1–5 of a 28-day cycle
- Cycles 2 to 6: 200 mg/m² orally 1 time daily on days 1–5 of a 28-day cycle

Schema 2: Diagram of Ritonavir Dose Escalation and Expansion Cohorts

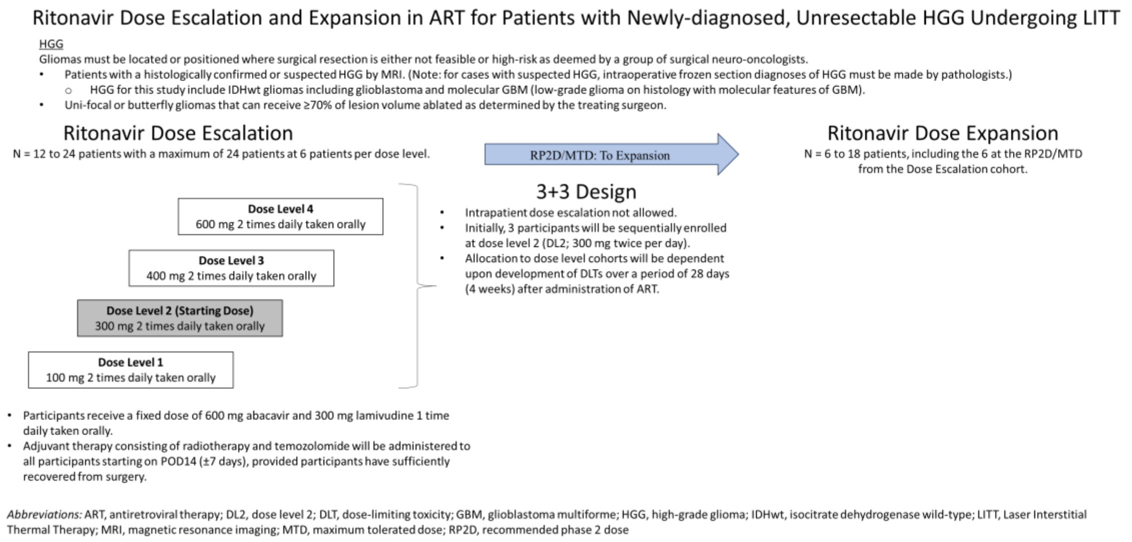

1 BACKGROUND

1.1 Study Rationale

Outcomes for glioblastoma remain poor despite maximal surgical resection and chemoradiation. To improve outcomes for this devastating disease, we will be synergistically targeting glioblastoma using minimally invasive laser interstitial thermal therapy (LITT) and antiretroviral therapy (ART) to potentiate chemoradiation. This will be a first-in-human study assessing the effects of LITT and ART for newly-diagnosed high-grade glioma (HGG).

1.2 Background on Glioblastoma

As reported by Khansur, Shah, Lacy, and Komotar, "Glioblastoma is the most lethal primary central nervous system tumor with an incidence rate of 3.19 per 100,000 person-years, averaging around 13,000 cases diagnosed in the United States per year (Reardon and Mitchell, 2017). Over the last 15 years, the treatment for glioblastoma multiforme (GBM) included maximal safe surgical resection with combination radiotherapy and adjuvant temozolomide chemotherapy (Stupp et al, 2009). Despite this treatment, the overall 5-year survival still remains poor with an average survival of 14 months after initial diagnosis (Stupp et al, 2009; Coffey, Lunsford, and Taylor, 1988; Ma et al, 2009). … Because of the dismal prognosis, attention has shifted to alternative adjuvant treatment modalities" (Khansur et al, 2019).

Traditionally, patients who are not candidates for surgical resection are offered biopsy alone as surgical treatment; however, survival in this select cohort remains a dismal 9 months after adjuvant chemoradiation (Shah et al, 2019). As a result, attention has shifted to other surgical adjuncts to improve tumor cytoreduction without compromising patient safety. Recently, magnetic resonance (MR)-guided LITT has been described for treatment of newly-diagnosed gliomas (Shah et al, 2019; de Groot et al, 2022).

1.3 Background on Magnetic Resonance Imaging-guided Laser Interstitial Thermal Therapy

For newly-diagnosed GBM (nGBM), stereotactic biopsy performed at the time of LITT trajectory planning enables diagnosis and cytoreduction through a single minimally invasive procedure. This benefits patients who would otherwise only be eligible for biopsy alone plus adjuvant chemoradiation, which is associated with survival time of 7–9 months (Fazeny-Dorner et al, 2003). Previous studies have showed that LITT for nGBM is associated with a survival benefit comparable to treatment with the conventional Stupp protocol (Stupp et al, 2005; Shah et al, 2020). Our recent institutional series also reports a survival benefit to biopsy and LITT for primary treatment of patients with deep nGBM lesions compared to historical controls who were offered biopsy and chemoradiation alone (Shah et al, 2019; Shah et al, 2020).

Table 1. Clinical Outcomes by Subtype of Brain Lesion Treated with LITT

**Lesion subtype**	**Median EOA (%), (IQR)**	**Median length of follow-up (months), (IQR)**	**Recurrence, n (%)**	**Median time-to-recurrence (months)**	**Percentage local control at 1-year follow-up**	**Median OS (months)**	**Death, n (%)**	**Complications (n), description**
Meningioma*	82.5 (76.0–87.3)	14.8 (9.5–20.1)	1 (25.0%)	N/A	75.0	20.7	1 (25%)	1, transient facial palsy

Metastasis	100.0 (88.0–100.0)	7.6 (3.4–17.2)	9 (20.0%)	55.9	77.4	16.9	17 (47%)	2, post-op seizure, wound infection
nGBM	98.0 (88.5–100.0)	5.6 (1.8–25.4)	3 (27.3%)	31.9	83.3	32.3	5 (46%)	–
rGBM	87.5 (77.0–99.5)	7.3 (5.6–13.5)	9 (64.3%)	5.6	24.3	7.3	12 (86%)	1, wound infection
RN	100.0 (98.8–100.0)	4.4 (1.3–11.6)	5 (25.0%)	N/A	67.2	16.4	4 (30%)	–
Other	92.5 (79.5–98.8)	5.4 (4.0–19.7)	2 (33.3%)	12.3	80.0	24.4	0 (0%)	–

Abbreviations: EOA, extent of ablation; IQR, interquartile range; LITT, laser interstitial thermal therapy; N/A, not applicable; nGBM, newly-diagnosed glioblastoma; OS, overall survival; rGBM, recurrent glioblastoma; RN, radiation necrosis; TTR, time to recurrence

Note: Length of time measured from date of LITT procedure. Median TTR is not reported for lesion that did not achieve 50% recurrence subtypes (ie, meningioma and RN).

* Meningioma subcategory contains 1 Grade 3 malignant lesion which contributed to the single incidences of death and recurrence in this subtype. Analysis with only Grade 1 meningiomas would report zero recurrences or deaths, with no median local control or median OS to report.

Previously, we have demonstrated safety and efficacy of MR-guided LITT for primary deep inaccessible gliomas (Shah et al, 2019; Shah et al, 2020). In our initial cohort of newly-diagnosed HGGs (n=11), the mean age was 59 years with a median preoperative lesion size of 6.8 cm^3 and an overall survival (OS) greater than 30 months with an 83% 1-year local control rate (Shah et al, 2020). Regarding safety, LITT was associated with an adverse outcome rate of approximately 4% (seizures, superficial wound infection, and transient neurological deficit) for all cases in the series (n=91) (**Table 1**).

A potential mechanism for improved outcomes in patients with nGBM is transient activation of the immune system. Previous studies have suggested that LITT activates the immune system and transiently opens the blood brain barrier (Leuthardt et al, 2016). We have demonstrated that the neutrophil to lymphocyte ratio (NLR) after LITT significantly changes in the immediate postoperative period and is associated with improved OS (OS; delta neutrophil to lymphocyte ratio [DNLR]> 7.0:440 days vs. DNLR<7.0: 239 days, P=0.0297). This preliminary data suggests that monitoring the inflammatory response after LITT in patients with nGBM offers a potential prognostic measurement to assist in predicting treatment efficacy and OS for patients with glioblastoma (Figueroa et al, 2020). Additionally, there is sufficient evidence that LITT activates both the adaptive and innate immune system in both preclinical and clinical models (Srinivasan et al, 2020; Shin et al, 2021). In non-glioma preclinical models, LITT has also stimulated the tumor-specific cytolytic T-cells (Vogl et al, 2009).

1.4 Background on Human Endogenous Retroviruses

Though human endogenous retroviruses (HERVs) make up approximately 8% of the human genome, they have lost their ability to replicate and form viral particles. However, HERVs may be re-expressed in pathologic conditions with epigenetic dysregulation such as amyotrophic lateral sclerosis (ALS), multiple sclerosis (MS), and cancer. Targeting this viral deoxyribonucleic acid (DNA) and its corresponding regulators may improve treatment response in patients with cancer (Shah et al, 2021).

1.5 Use of Antivirals as Treatment for High-Grade Gliomas

Numerous antiretroviral drugs, eg, the front-runner nelfinavir, are known to exhibit antitumor activity and have already been tested for their off-label use in cancer (Chow, Jiang, and Guan, 2009).

Unpublished data from our group supports that monotherapy with lamivudine, raltegravir, and abacavir, among others, has shown some antitumor effect in experimental models of GBM, but it's true combinatorial versatility is yet unknown. We have confirmed the efficacy of the drug combination abacavir, dolutegravir and lamivudine in both established glioma cell cultures and in patient-derived neurospheres *in vitro*. Using the drug repositioning application DepMAP, we have determined that the drug combination of abacavir, lamivudine, and ritonavir has broad efficacy among most glioma cells at therapeutic concentrations (2.5 μM). Additionally, abacavir alone sufficiently decreased cell viability of GBM neurospheres and reduced cancer stemness at sublethal concentrations (**Figure 1**). Our preclinical work has demonstrated that abacavir induces glioma cell death and reduces stemness markers Nestin and OCT4 (**Figure 2**). Therefore, these drugs would be excellent candidates for drug repositioning.

Additionally, the combination anti-retroviral treatment of abacavir, lamivudine, and ritonavir would be an ideal candidate for glioma clinical trials. Since all three drugs permeate the blood brain barrier and have marked efficacy in gliomas, the combination treatment may be ideal at targeting oncogenic transposable elements including HERV-K and glioma stem cells. Since LITT is known to open the blood brain barrier for 2–8 weeks after surgery, using antiretroviral therapy (ART) during the perioperative period may synergistically improve outcomes for this disease.

Figure 1: Efficacy of antiretrovirals against patient-derived glioma neurospheres using XTT cell viability assay which is confirmed using pharmacogenetic drug screening approach (depmap.org).

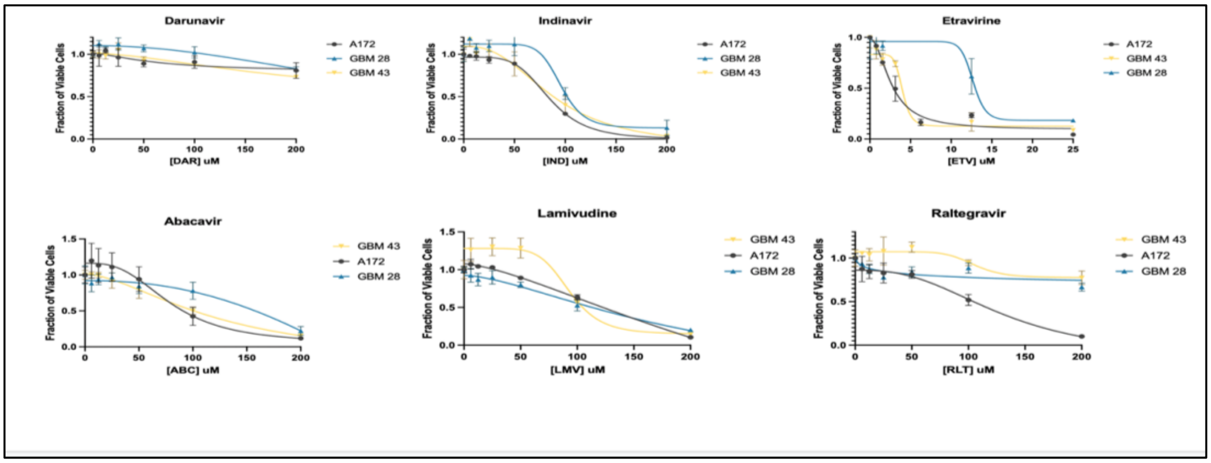

Figure 2: Reverse transcriptase inhibitors are efficacious at targeting glioma cells.
Unbiased screen of ART identifies several candidate drugs with potent antiglioma activity. (A) Heatmap depicting a correlation matrix that represents the comprehensive evaluation of antiretroviral drug efficacy against glioma cell lines using the Cancer Dependency Map (DepMap) database. 16 antiretroviral drugs were identified from 4518 drugs in the PRISM Repurposing Screen. Heatmap illustrating the impact of antiretroviral drugs on the survival of individual glioma cell lines in terms of log2fold change (range = -2.13 , 1.92, SD= 0.4692). Data sourced from the PRISM Repurposing Screen and analyzed using DepMap, with warmer colors indicating improved survival and cooler colors indicating reduced survival. Reverse transcriptase inhibitors decrease stemness and self-renewal capacity. (B) Abacavir and Lamivudine decrease GBM markers of stemness (OCT4) and invasion (Vimentin) at therapeutic dosages (20 µM, for 48 hrs, A172). (C) NRTIs such as Abacavir and Lamividuine significantly decreased proliferation compared to control in xCelligence assay. (ANOVA, $p<0.05$, ** $p<0.01$, *** $p<0.001$, **** $p<0.0001$)

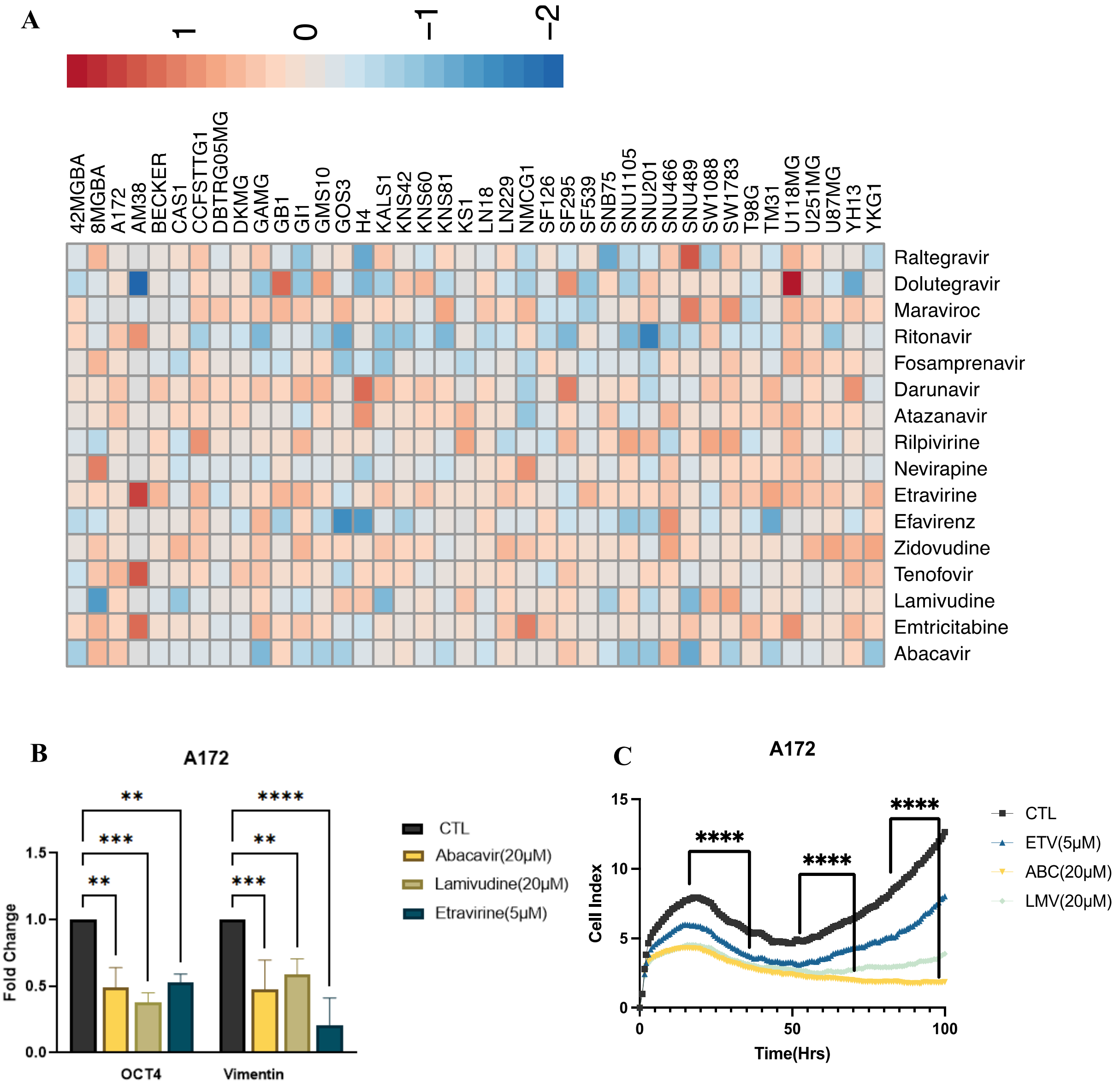

1.6 Rationale for the Combination of Antiviral Therapy and Magnetic Resonance Imaging-guided Laser Interstitial Thermal Therapy

The combinatorial effects of ART (abacavir, lamivudine, and ritonavir) with LITT, temozolomide, and radiotherapy have yet to be studied. Given the high attrition rates of candidate compounds in the translational process from basic science to regulatory approval, repositioning established drugs for new therapeutic purposes appears as a promising shortcut strategy to accelerate drug discovery (Chow, Jiang, and Guan, 2009). The concept has already been successfully applied in a variety of medical conditions, eg, multiple myeloma, breast cancer, non-small cell lung cancer (Wang et al, 2020), or colorectal cancer. In this regard, Rauschenbach et al. (2020) pointed out that antiviral protease inhibitors, such as ritonavir (RTV) showed antitumor effects on a molecular subclass of GBM. Overall, RTV resulted in cytostatic, anti-migratory, and radiosensitizing/chemosensitizing effects (Rauschenbach et al, 2020). Additionally, we have demonstrated that glioma patients with human immunodeficiency virus (HIV) who are treated with antiretrovirals have a significantly improved survival compared to untreated patients (Mendez Valdez et al, 2022).

Therefore, we propose a novel study assessing the efficacy of oral ART in combination with LITT chemotherapy and radiation therapy for patients with newly-diagnosed, unresectable HGG. The primary goal of this study is to determine the maximum tolerated dose (MTD) and/or the recommended phase 2 dose (RP2D) of ritonavir, since the appropriate dose of ritonavir is unclear, while abacavir (600 mg one time per day) and lamivudine (300 mg one time per day) are fixed.

We hypothesize that concurrent ART (abacavir, lamivudine, and ritonavir) with LITT and adjuvant therapy (temozolomide + radiotherapy) for patients with newly-diagnosed, unresectable HGG is efficacious when compared to historical controls.

1.7 Benefit/Risk Assessment

1.7.1 Assessment of Potential Benefits and Risks

There are several potential benefits to receiving the combination of ART and LITT for gliomas. In preclinical studies, ART decreases tumor size and effectively kills glioblastoma cells (**Section 1.5**). In some early clinical trials in cancer, ART has produced clinical responses (stable or partial responses) in treatment refractory cancers (Chow, Jiang, and Guan, 2009). Using this data, ART may be able to synergistically activate chemotherapy and radiation and may improve radiographic response and clinical outcomes for patients with glioblastoma.

As described in the United States Package Insert (USPI) for abacavir+lamivudine tablets, taking abacavir can result in serious or fatal hypersensitivity reactions. Participants who carry the human leukocyte antigen-B variant *5701 (HLAB*5701) allele are at a higher risk of such reactions. For this reason, all potential study participants will be screened for this allele prior to starting ART, and those who have the variant allele or a history of sensitivity to abacavir or lamivudine will be excluded from participation (**Section 3.2**). Since patients co-infected with hepatitis B and/or C and HIV can have exacerbation of hepatitis when taking lamivudine and to avoid any confounding factors of previous ART exposure, patients with a history of HIV infection will also be excluded from study participation (**Section 3.2**). Furthermore, patients with impaired kidney and/or liver function as described in **Section 3.2** will also be excluded from study participation. Since lactic acidosis and severe hepatomegaly with steatosis, including fatal cases, have been reported with the use of nucleoside analogues (abacavir+lamivudine USPI), study personnel will monitor participants for potential adverse reactions associated with ART, such as hepatic toxicity and adverse changes in blood chemistry or organ function, as described in **Sections 7**, **8**, and **10.1**.

Since participants are receiving combination surgical treatment and ART, there may be an increased risk of bleeding events when combining surgery with ritonavir use (ritonavir USPI). For example, in patients with HIV and hemophilia (type A and B) being treated with protease inhibitors, including ritonavir, increased bleeding, including spontaneous skin hematoma and hemarthrosis, were reported, and during the clinical trial, 2.3% of patients receiving treatment with ritonavir reported episodes of gastrointestinal (GI) bleeding. Bleeding episodes occurred at a median of 22 days after initiation of protease inhibitor therapy. In spite of these bleeding events, the majority of patients were able to continue taking their protease inhibitor therapy though some patients received additional coagulation factors. In a brief search of the literature, no reports of intracranial bleeding have been described in patients without hemophilia receiving ritonavir monotherapy. Ritonavir use is also associated with high cholesterol and hyperglycemia; therefore, patients will be monitored for abnormal changes in blood chemistry or organ function as described in **Sections 7**, **8**, and **10.1**

In addition to the risks listed above, there may be some unknown risks and side effects associated with use of these medications in patients with HGG versus patients with HIV, especially when combined with LITT and standard of care adjuvant radiotherapy and temozolomide treatment. To facilitate early identification and management of adverse events (AEs), study personnel will monitor participants closely for dose-limiting toxicities (DLTs) during the first 28 days (4 weeks) after administration of ART (**Section 4.5.2**) and assess participants for side effects periodically throughout the remainder of the study (**Sections 7**, **8** and **10.1**).

2 OBJECTIVES AND ENDPOINTS

**Objectives**	**Endpoints**
**Primary**	
To determine maximum tolerated dose (MTD) and/or recommended phase 2 dose (RP2D) of ritonavir in combination of fixed dose of abacavir and lamivudine in patients with newly-diagnosed, unresectable HGG.	Frequency and severity of AEs. For dose-limiting toxicity (DLT) as per National Cancer Institute Common Terminology Criteria for Adverse Events version 5.0 (NCI CTCAE v5.0), number of DLTs during the first 28 days (4 weeks) after the administration of ART (**Section** 4.5.2).
	As per **Section** 4.5.5, DLT, assessed by NCI CTCAE v5.0, is defined as the manifestation of one or more of the following treatment-related adverse events (TEAEs) during the first 28 days (4 weeks) after administration of ART:<br>• Hematologic Events<br>o Grade 4 neutropenia lasting ≥ 7 days (granulocyte colony-stimulating factor [GCSF] use allowed)<br>o Grade 4 anemia<br>o Grade 4 thrombocytopenia, grade 3 if associated with bleeding<br>o Febrile neutropenia<br>• Non-hematologic ≥Grade 4 toxicity<br>o Hepatic impairment<br>• Uncontrolled diarrhea that is unresponsive to supportive care, medications, and dietary changes<br>• Wound dehiscence (≥Grade 3)<br>• Symptomatic cerebral edema with worsening midline shift > 10 mm (≥Grade 4) with neurological deficits<br>• Symptomatic intracranial hemorrhage (≥Grade 4)<br>• Any TRAE that leads to >25% of the doses missed during the first 4 weeks
**Secondary**	
To estimate progression-free survival (PFS) and overall survival (OS) for patients with newly-diagnosed, unresectable HGG.	Progression-free survival (PFS) is defined as the elapsed time from start date of LITT to first documented evidence of disease progression or death from any cause, whichever is earlier. For surviving patients without progression, follow-up will be censored at the date of last documented progression-free status (**Section** 10.4.1.1).

Objectives	Endpoints
	Overall survival (OS) defined as the elapsed time from start date of LITT to death from any cause (**Section 10.4.1.2**). For surviving patients, follow-up will be censored at the last date known to be alive.
**Exploratory**	
To assess preliminary clinical efficacy of ART+LITT as measured by the Response Assessment in Neuro-Oncology (RANO) criteria (Leao et al, 2019) (**Section 10.2**), in terms of radiographic objective response rate (ORR=complete response [CR]+partial response [PR]) and duration of radiographic response (DoR).	Radiographic objective response rate (ORR; complete response [CR] or partial response [PR]) after LITT as determined by the RANO criteria (**Section 10.4.1.2**)<br><br>Duration of response (DoR) to treatment by the RANO criteria (**Section 10.4.1.2**)

3 STUDY POPULATION

3.1 Inclusion Criteria

1. Age ≥ 18 years.
2. Patients with a histologically confirmed or suspected HGG by MRI.
    a. For cases with suspected HGG, intraoperative frozen section diagnoses of HGG must be made by pathologists (**Section** 4.4.1).
    b. HGG for this study include IDH wild-type gliomas including glioblastoma and molecular GBM (low-grade glioma on histology with molecular features of GBM).
3. Uni-focal or butterfly gliomas that can receive ≥70% of lesion volume ablated as determined by the treating surgeon.
4. Gliomas must be located or positioned where surgical resection is either not feasible or high-risk as deemed by a group of surgical neuro-oncologists.
5. Preoperative Karnofsky score ≥ 70 (APPENDIX A).
6. Patients must have demonstrable normal organ function as defined below within 14 days of surgery.
    a. Absolute neutrophil count (ANC) ≥ 1500 cells/mm^3
    b. Platelets ≥ 100,000 cells/mm^3
    c. Hemoglobin ≥ 9.0 g/dL. Use of transfusion or other intervention to achieve this hemoglobin level is acceptable.
    d. Blood urea nitrogen (BUN) ≤ 35 mg/dL and creatinine ≤ 1.9 mg/dL and estimated glomerular filtration rate (eGFR) or creatinine clearance rate > 50 mL per minute.
    e. Electrocardiogram (ECG) without evidence of acute cardiac ischemia.
    f. Prothrombin time (PT)/International Normalized Ratio (INR) <1.4
    g. Liver function tests: Aspartate aminotransferase (AST) and alanine transaminase (ALT) at or below 2.5 times the upper limit of normal (ULN).
    h. Sodium level > 130 mg/L. Use of salt resection or hypertonic saline to achieve this sodium level is acceptable.
7. Patients must be able to understand and sign informed consent.

3.2 Exclusion Criteria

1. Patients with HLA-B*5701 hypersensitivity (**Section** 10.1.6.7). NOTE: patients with a pending HLA-B test result may still undergo the LITT procedure as part of their standard medical care. However, the HLA-B test result must be obtained prior to a participant starting ART as part of this study.
2. Patients with sensitivity to abacavir, lamivudine, or ritonavir (**Section** 7.3.1).
3. Patients with a previous history of HIV infection.
4. Patients with uncontrolled hepatitis B or C infection.
5. Patients who have received any surgical resection for this tumor.
    a. Patients who have received an open biopsy for this disease are still eligible for participation.
6. Patients who have received chemotherapy or radiation for this disease.
7. Patients who are taking dofetilide (**Section** 4.10.1).
8. Patients on a regimen of 1 or more prohibited medications as described in **Section** 4.10.1 that cannot be discontinued or switched to a more compatible medication. For more information on prohibited and precautionary use medications for patients on this study, please see **Section** 4.10.
9. Patients not eligible to obtain MRI with and without contrast.
10. Recurrent HGG.
11. Presence of current infection, such as sepsis, meningitis, bacteremia, or pneumonia.

12. Fever within 48 hours of surgery (Temperature> 38.0°C).
13. Severe co-morbidity that would confer excess risk of surgery, radiation, or chemotherapy, as determined by the treating physician.
14. Any co-morbidity or psychiatric ailment that in the Investigator's opinion will prevent administration or completion of protocol therapy.
15. Pregnant women.
16. Patients must be willing to use contraception as described in **Section 4.11**.
17. Patients receiving other investigational agents or concurrent enrollment in another therapeutic clinical trial.
18. Prisoners.
19. Adults unable to consent.

3.3 Recruitment and Enrollment

3.3.1 Facilities for Recruiting and Enrolling Participants

Investigators and designated personnel from the research study team at the University of Miami (UM) Sylvester Comprehensive Cancer Center (SCCC) and if applicable other sites (for multi-center studies) will identify, recruit, enroll and treat eligible participants for the study. Study personnel will access electronic medical record or other protected health information (PHI) without obtaining a signed Health Insurance Portability and Accountability Act of 1996 (HIPAA) authorization from the patient to identify potential participants for recruitment. Participants will be recruited at the UM SCCC via clinical practice offices.

3.3.2 Recruitment Methods

We plan on recruiting participants through patient advocacy groups (Glioblastoma Research Organization), online services (Google advertisements), and through social media platforms (Twitter, Instagram). Potential screening applicants will be seen in clinic for evaluation by our neurosurgical team, and eligibility will be determined based on the aforementioned criteria (**Sections 3.1** and **3.2**). No financial incentive will be offered to study participants.

4 STUDY DESIGN AND TREATMENT PLAN

4.1 Study Design

This study will be a single institution, single-arm, phase 1 study to determine the RP2D of ritonavir as part of the ART regimen of abacavir, lamivudine, and ritonavir followed by standard of care adjuvant therapy (temozolomide + radiotherapy) in patients with newly-diagnosed, unresectable HGG post LITT. The study will consist of two parts: dose escalation/de-escalation of ritonavir (Part 1) with a traditional 3+3 design and dose expansion (Part 2).

4.2 Study Population

Investigators will enroll any patient with either a confirmed or presumed case of HGG that has not received a surgical resection or adjuvant treatment. LITT will be offered to patients who are not amenable to surgical resection as determined by a group of surgical neuro-oncologists. A total sample size of 24 eligible patients will be enrolled on this study.

4.3 Setting–Description of Facilities Enrolling Participants

Study personnel will identify, recruit, enroll and treat participants by Investigators at the UM SCCC inclusive of all satellite sites. Sylvester Comprehensive Cancer Center (SCCC) will be the only study site.

4.4 Treatment Plan

Enrolled participants will receive study treatment as described in **Table 2**.

Table 2. Overall Description of Study Treatment Procedures and Medication Administration

Study Procedure or Medication	POD0	POD7*	POD14 ±7 days	POD57 ±3 days	POD86 ±2 days	POD255 ±3 days to POD365 ±2 days or until disease progression**
**LITT**	Single-stage procedure following stereotactic needle biopsy	Not performed	Not performed	Not performed	Not performed	Not performed
**ART†**						
**Abacavir+ Lamivudine**	Not administered	1 tablet consisting of 600 mg of abacavir and 300 mg of lamivudine 1 time daily	1 tablet consisting of 600 mg of abacavir and 300 mg of lamivudine 1 time daily	1 tablet consisting of 600 mg of abacavir and 300 mg of lamivudine 1 time daily	1 tablet consisting of 600 mg of abacavir and 300 mg of lamivudine 1 time daily	1 tablet consisting of 600 mg of abacavir and 300 mg of lamivudine 1 time daily
**Ritonavir**	Not administered	2 times daily; Dose assignment per 3+3 design (**Table 3**)	2 times daily; Dose assignment per 3+3 design (**Table 3**)	2 times daily; Dose assignment per 3+3 design (**Table 3**)	2 times daily; Dose assignment per 3+3 design (**Table 3**)	2 times daily; Dose assignment per 3+3 design (**Table 3**)

Adjuvant Therapy						
**RT**	Not administered	Not administered	Focal RT administered for 6 weeks (42 days) as described in **Section 4.4.5**	Not administered	Not administere d	Not administered
**TMZ**	Not administered	Not administered	Administered at 75 mg/m² orally 1 time daily on a continuous schedule (including weekends and holidays) for 6 weeks (42 days) (**Section 4.4.5**).	Start of a 4-week (±2 week) rest period prior to start of adjuvant TMZ treatment (**Section 4.4.5**).	Adjuvant Therapy: Cycle 1: 150 mg/m² orally 1 time daily on days 1–5 of a 28-day cycle. Cycles 2 to 6: 200 mg/m² orally 1 time daily on days 1–5 of a 28-day cycle (**Section 4.4.5**)	Not administered

Abbreviations: ART, antiretroviral therapy; LITT, laser interstitial thermal therapy; POD, postoperative day; RT, radiotherapy; TMZ, temozolomide

* Antiretroviral therapy (ART) may begin later if the participant is considered unable to begin treatment due to their postoperative condition, ie, inability to swallow medications (**Section 7.1.1**).

** Participants with a response of stable disease (SD) or better may continue to receive ART beyond the Study Treatment Period at the Investigator's discretion (**Section 4.7**).

† Participants with a pending HLA-B test result may still undergo the LITT procedure as part of their standard medical care. However, the HLA-B test result must be obtained prior to a participant starting ART as part of this study (**Section 10.1.6.7**). Participants with the HLA-B*5701 variant must be excluded from study participation as described in **Section 3.2**; however, these participants will continue to receive standard of care treatment, which includes radiotherapy and chemotherapy, as per the treating neuro-oncologist.

The study will be conducted in two parts as follows:

- **Part 1: Dose escalation/de-escalation of ritonavir** in the ART regimen will be conducted using at most four dose levels of ritonavir as described in **Table 3** to determine the potential MTD (or RP2D, if the MTD is not attained).
- **Part 2: As dose expansion cohort,** an additional 6–18 participants including 6 participants treated with the RP2D in part 1 will be enrolled to confirm the safety and tolerability of the RP2D of ritonavir in the ART regimen and to assess the preliminary efficacy of the treatment.

4.4.1 Pre- and Perioperative Activities

Participants will be evaluated by the neurosurgical team prior to surgery and will have either a suspected diagnosis of an HGG or a histologically confirmed HGG at the time of enrollment (**Section 3.1**). If frozen section diagnosis is equivocal or non-diagnostic of glioma, the participant(s) may continue to receive LITT at the surgeon's discretion and will be deferred for further study participation until the

histological review is completed (**Section 5.3**). Histological review at tumor board will occur within 7 days of the biopsy and further enrollment will be discussed among the study investigators and multidisciplinary team.

Since the turnaround period for HLA-B screening is approximately 3 to 7 days, participants with a pending HLA-B test result may still undergo the LITT procedure as part of their standard medical care. However, the HLA-B test result must be obtained prior to a participant starting ART as part of this study (**Section 10.1.6.7**). Participants with the HLA-B*5701 variant must be excluded from study participation as described in **Section 3.2**; however, these participants will continue to receive standard of care treatment, which includes radiotherapy and chemotherapy, as per the treating neuro-oncologist.

4.4.2 Magnetic Resonance Imaging-guided Laser Interstitial Thermal Therapy

Laser interstitial thermal therapy (LITT) is a single-stage procedure that immediately follows stereotactic needle biopsy (**Section 10.1.9**) and follows the same surgical tract as the initial biopsy in single trajectory cases. For cases with multiple trajectories, the second tract will only include the laser fiber insertion. The degree of laser ablation will be determined by the treating neurosurgeon and presence of eloquent or surrounding eloquent functional cortex/white matter tracts. For more information regarding the LITT procedure, please refer to the Visualase Operator's Manual. For stereotactic needle biopsy procedure, the neurosurgeon will procure cores of tissue at target depth and at the leading edge of the tissue. Laser fiber placement will be determined by the treating neurosurgeon based on the tumor location, biopsy location, lesion heatsinks, and vasculature. The treating neurosurgeon should prioritize a trajectory to maximize ablation of the tumor tissue along the long axis of the lesion. Laser ablation will begin in the MRI suite at test dose (30% of maximal power) to ensure target ablation. Once test dose is confirmed, ablation will continue at increments of 10–20% increases for 2:00–3:00 minute intervals until maximum temperatures have plateaued. High-temperature protective marks will be placed parallel to laser fiber and low-temperature protective marks will be placed to protect adjacent brain parenchyma per the surgeon's discretion. Once maximum ablation is achieved at desired depth, the catheter may be pulled back 5–10 mm to maximize ablation along the trajectory. The surgeon will continue ablation until maximal safe ablation is achieved. The Thermal Damage Estimate will be measured against lesion size on two perpendicular views along the catheter axis to determine the relative extent of ablation.

4.4.3 Post-operative Care and Monitoring

Participants will be admitted to the Intensive Care Unit (ICU) and monitored inpatient for 24 hours according to institutional standards.

4.4.4 Antiretroviral Therapy (ART)

Antiretroviral therapy (ART) consisting of abacavir, lamivudine, and ritonavir will be administered to participants 7 days after surgery (POD7), provided participants have sufficiently recovered from surgery (**Section 7.1.1**). We will test participants prior to starting ART for HLA-B*5701 hypersensitivity due to interactions with abacavir (**Section 10.1.6.7**). For more information on dosing and administration of ART, please see **Section 4.5**. Participants will be seen by a neuro-oncologist prior to receiving their first dose of ART (**Section 8**). The neuro-oncologist will manage the ART therapy.

4.4.5 Adjuvant Therapy

Adjuvant therapy consisting of radiotherapy and temozolomide will be administered to all participants starting on POD14 ± 7 days, provided participants have sufficiently recovered from surgery (**Section 7.1.2**). For more information on dosing and administration of radiotherapy and temozolomide, please see **Sections 4.5.6.1** and **4.5.6.2**, respectively.

4.5 Dosing and Administration

The ART therapy will be obtained from the research pharmacy and dispensed to participants free of charge. ART medications will be administered as described in the following sections (**Sections 4.5.1** and **4.5.2**). Starting from POD7 (or later, depending on participants' postoperative condition [**Section 7.1.1**]), the Investigator will ask participants to record the date, time, and dose of ART medications taken in a monthly medication diary. Participants will be asked to record their ART medication as soon as they take their medicines and to bring their medication diaries with them to every clinic visit as listed in the Schedule of Assessments (**Section 8**).

4.5.1 Abacavir and Lamivudine

The abacavir+lamivudine tablets will be self-administered orally one time per day at a fixed dose of 600 mg abacavir and 300 mg lamivudine according to the USPI. The following instructions will be provided to participants:

Instructions for Taking Abacavir+Lamivudine

- Take abacavir+lamivudine tablets exactly as your healthcare provider tells you to take it.
- Do not change your dose or stop taking abacavir+lamivudine without talking with your healthcare provider.
- If you miss a dose of abacavir+lamivudine, take it as soon as you remember. Do not take 2 doses at the same time or take more than your healthcare provider tells you to take.
- Stay under the care of a healthcare provider during treatment with abacavir+lamivudine.
- Abacavir+lamivudine tablets may be taken with or without food.
- Tell your healthcare provider if you have trouble swallowing abacavir+lamivudine tablets.
- Do not run out of abacavir+lamivudine. When your supply starts to run low, get more from your healthcare provider or pharmacy.
- If you take too much abacavir+lamivudine, call your healthcare provider or go to the nearest hospital emergency room right away.

4.5.2 Ritonavir

Ritonavir tablets will be self-administered orally twice per day at the assigned dose as described in **Table 3**. The following instructions will be provided to participants:

Instructions for Taking Ritonavir

- Take ritonavir exactly as your Study Doctor tells you to take it.
- You should stay under a healthcare provider's care during treatment with ritonavir. Do not change your dose of ritonavir or stop your treatment without talking with your healthcare provider first.
- Swallow ritonavir tablets whole. Do not chew, break, or crush tablets before swallowing. If you cannot swallow the ritonavir tablets whole, tell your healthcare provider. You may need a different medicine.
- Take ritonavir tablets with meals.
- Do not run out of ritonavir. Get your ritonavir prescription refilled from your healthcare provider or pharmacy before you run out.
- If you miss a dose of ritonavir, take it as soon as possible and then take your next scheduled dose at its regular time. If it is almost time for your next dose, wait and take the next dose at the regular time.
- Do not double the next dose.

- If you take too much ritonavir, call your local poison control center or go to the nearest hospital emergency room right away.

4.5.2.1 Dose Escalation/De-Escalation Rules for Ritonavir and Dose Expansion

Part 1: For dose escalation/de-escalation of ritonavir, we will use the traditional 3+3 design. In order to determine the MTD and/or RP2D for ritonavir, four dose levels are planned as described in **Table 3**.

Table 3. Dose Levels for Ritonavir

Dose Level	Dosage and Frequency
1	100 mg 2 times daily
2 (Starting Dose)	300 mg 2 times daily
3	400 mg 2 times daily
4	600 mg 2 times daily

The starting dose is dose level 2 (300 mg twice per day) with potential escalation to dose level 4 (600 mg twice per day) and de-escalation to dose level 1 (100 mg twice per day). Allocation to dose level cohorts will be dependent upon development of DLTs over a period of 28 days (4 weeks) after administration of ART. The clinical signs/symptoms considered DLTs for this study are described in **Section 4.5.5**.

Initially, 3 participants will be sequentially enrolled at dose level 2 (DL2; 300 mg twice per day). Using the 3+3 design, the following rules will be employed for dose escalation/de-escalation:

1. If no DLT out of 3 participants is experienced, the dose for the next participant will be escalated to the dose level 3 (DL3; 400 mg twice per day).
2. If 2 out of 2–3 participants experience a DLT, the dose for the next participant will be de-escalated to the dose level 1 (DL1; 100 mg twice per day).
3. If 1 out of 3 participants experiences a DLT, 3 additional participants will be sequentially enrolled in the same DL2.
    - Among the 6 participants, if 1 participant experiences a DLT, dose will be escalated to DL3 (400 mg twice per day).
    - If 2 out of 4–6 participants experience a DLT, dose will be de-escalated to DL1 (100 mg twice per day).

Once the dose is escalated to DL3, the same dose escalation/de-escalation rules applied at DL2 will be conducted at DL3. The highest dose with no more than 1 out of 6 participants experiencing a DLT will be considered as the RP2D.

Part 2: Dose expansion will be conducted to confirm safety and tolerability of the determined RP2D from the dose-finding part (Part 1) and to assess the preliminary efficacy of the treatment. Based on our 3+3 design in part 1, the number of participants for dose escalation/de-escalation is between 12–18 (if only dose levels 2, 3, and 4 are tested) and maximum 24 (if all four dose levels are tested with 6 participants per level). Thus, the expected total number of participants in part 1 is 12–24. An additional 6–18 participants including 6 participants treated with the RP2D in part 1 will be enrolled in dose-expansion cohort to be treated at the RP2D of ritonavir.

4.5.3 MTD of Ritonavir

The maximum tolerated dose (MTD) may not be reached if the highest dose tested (DL4) is found to be safe (that is, DLT in ≤1 out of 6 participants). In this case, DL4 may be the potential RP2D without declaration of an MTD.

4.5.4 Potential RP2D of Ritonavir

The recommended phase 2 dose (RP2D) will be defined as the highest dose level at which ≤1 out of 6 participants (<20%) experiences DLT. If unacceptable toxicity occurs (DLT in 2 out of 2–6 participants) at the reduced DL1, the trial will terminate without finding an RP2D.

4.5.5 Definition of Dose-Limiting Toxicities for Ritonavir

Dose-limiting toxicity (DLT) and disease assessment, assessed by NCI CTCAE v5.0, is defined as the manifestation of one or more of the following TRAEs during the first 28 days (4 weeks) after administration of ART:

- Hematologic Events
  - Grade 4 neutropenia lasting ≥ 7 days (GCSF use allowed)
  - Grade 4 anemia
  - Grade 4 thrombocytopenia, grade 3 if associated with bleeding
  - Febrile neutropenia
- Non-hematologic ≥Grade 4 toxicity
  - Hepatic impairment
- Uncontrolled diarrhea that is unresponsive to supportive care, medications, and dietary changes
- Wound dehiscence (≥Grade 3)
- Symptomatic cerebral edema with worsening midline shift > 10 mm (≥Grade 4) with neurological deficits
- Symptomatic intracranial hemorrhage (≥Grade 4)
- Any TRAE that leads to >25% of the doses missed during the first 4 weeks

4.5.6 Adjuvant Therapy

4.5.6.1 **Radiotherapy**

Radiotherapy will be administered for 6 weeks (42 days). Focal radiotherapy will be administered per institutional guidelines at a total dose of 60 Gy in 1.8–2.0 Gy fractions (30 total) depending on prognosis and as determined by the treating radiation oncologist. This standard of care is based on the Stupp protocol published in 2005 which uses 60 Gy delivered over 30 fractions given Monday through Friday over 6 weeks with concurrent and adjuvant temozolomide (Stupp et al, 2005). The gross tumor volume will be treated with a 2- to 3-cm expansion respecting anatomical boundaries to create a clinical target volume encompassing microscopic disease as determined by the radiation oncologist(s) with 3 to 5 mm expansion on the clinical target volume to account for daily treatment variation.

4.5.6.2 **Temozolomide**

Temozolomide will be administered concurrently with radiotherapy over a period of 6 weeks (42 days) as follows:

- Orally at a dose of 75 mg/m² one time per day on a continuous dosing regimen, including weekends and holidays.

Following completion of radiotherapy and the 6-week (42-day) regimen of 75 mg/m² temozolomide, participants will have a 4-week (±2 week) rest period before starting adjuvant temozolomide treatment.

Adjuvant temozolomide will be administered for 6 28-day cycles (168 days total) as follows:

- Cycle 1: orally at a dose of 150 mg/m² one time per day on days 1–5 of a 28-day cycle
- Cycles 2 to 6: orally at a dose of 200 mg/m² one time per day on days 1–5 of a 28-day cycle

Please note that dosage and frequency of temozolomide administration may be altered at the discretion of the treating neuro-oncologist. Temozolomide dose may be rounded up or down in accordance with institutional standards to match tablet strength and availability at the local pharmacy. The following instructions will be provided to participants:

Instructions for Taking Temozolomide

- Take temozolomide capsules exactly as your doctor tells you to.
- Temozolomide capsules contain a white capsule body with a color cap and the colors vary based on the dosage strength. Your doctor may prescribe more than 1 strength of temozolomide capsules for you, so it is important that you understand how to take your medicine the right way. Be sure that you understand exactly how many capsules you need to take on each day of your treatment and what strengths to take. This may be different whenever you start a new cycle. In medicine, a cycle is treatment given on a set schedule with periods of rest in between.
- Do not take more temozolomide than prescribed.
- Talk to your doctor or pharmacist before taking your dose if you are not sure how much temozolomide to take. This will help to prevent you from taking too much temozolomide and decrease your chances of getting serious side effects.
- Take each day's dose of temozolomide capsules at one time, with a full glass of water.
- Swallow temozolomide capsules whole. Do not chew, open, or split the capsules.
- Take temozolomide capsules at the same time each day.
- Take temozolomide the same way each time, either with food or without food, unless instructed by your Study Doctor or pharmacist.
- If temozolomide capsules are accidentally opened or damaged, be careful not to breathe in (inhale) the powder from the capsules or get the powder on your skin or mucous membranes (for example, in your nose or mouth). If contact with any of these areas happens, flush the area with water.
- To help reduce nausea and vomiting, try to take temozolomide on an empty stomach or at bedtime. Your doctor may prescribe medicine to help prevent or treat nausea or other medicines to reduce side effects with temozolomide.
- See your doctor regularly to check your progress. Your doctor will check you for side effects.
- If you take more temozolomide than prescribed, call your doctor or get emergency medical help right away.

4.6 Missed Doses and Adherence to Drug Administration

Participants who do not receive protocol therapy dose at the usual required time should be rescheduled as close to the original scheduled date as possible. An exception is made when rescheduling becomes, in the Investigator's opinion, medically unnecessary or unsafe because it is too close in time to the next scheduled evaluation. In that case, the missed dose should be abandoned (**Section 7.1**).

All doses of protocol therapy will be self-administered at home. The threshold for removing a participant from the study based on protocol therapy adherence will be determined by the Sponsor-Investigator. The definition for low adherence to protocol therapy is provided below as a guidance for the study team.

Low adherence to protocol therapy: Participants that receive less than 80% of the protocol therapy. This will be documented as a percentage of the protocol-mandated doses.

The information related to each trial drug administration, including the date, time, and dose of study drug, will be recorded on Velos. The Investigator will make sure that the information entered into the Case Report Form (CRF)/electronic Case Report Form (eCRF) regarding drug administration is accurate for each participant. Any reason for not adhering with study drug administration will be documented in the CRF/eCRF.

4.7 Access to Study Medications after End of Study Participation

Participants with a response of SD or better may continue to receive ART beyond the Study Treatment Period at the Investigator's discretion.

4.8 Concomitant Therapy

Pneumocystis pneumonia (PCP) prophylaxis will be provided to participants in accordance with institutional standards (**Section 7.3.4.3**).

During LITT, the Investigator or Investigator's designee will be treating participants with steroids to mitigate the associated swelling from this procedure (**Section 7.3.2**). Additional supportive care measures post-LITT will be provided in accordance with institutional standards.

Supportive care measures for radiotherapy will also be provided in accordance with institutional standards (**Section 7.3.3**).

Participants will be placed on an anti-nausea regimen during temozolomide treatment in accordance with institutional standards.

4.9 Premedication

Premedications to be administered to participants at the discretion of the Investigator.

4.10 Prohibited and Precautionary Use Therapies

4.10.1 Prohibited Therapies

No other study treatment or other anti-cancer treatment will be allowed while participants are in this study.

Per **Section 3.2**, participants may not take dofetilide while participating in this study.

As described in the USPI, "Ritonavir is an inhibitor of cytochrome P450 (CYP) 3A (CYP3A) and may increase plasma concentrations of agents that are primarily metabolized by CYP3A. Agents that are extensively metabolized by CYP3A and have a high first-pass metabolism appear to be the most susceptible to large increases in area under the curve (AUC) (greater than 3-fold) when co-administered with ritonavir. Co-administration of ritonavir with drugs highly dependent on CYP3A for clearance and for which elevated plasma concentrations are associated with serious and/or life-threatening events is contraindicated" (**Section 3.2**). Co-administration with other CYP3A substrates may require a dose adjustment or additional monitoring as shown in **Table 4**. Please note that initiation of medications that inhibit or induce CYP3A may increase or decrease concentrations of ritonavir, respectively. These interactions may lead to:

- Clinically significant adverse reactions, potentially leading to severe, life-threatening, or fatal events from greater exposures of concomitant medications.
- Clinically significant adverse reactions from greater exposures of ritonavir.

- Loss of therapeutic effect of ritonavir and possible development of resistance (in patients with HIV).

Additional medications that are contraindicated or prohibited for use with ritonavir in participants in this study are as follows (see Contraindications (4) section of the ritonavir USPI):

- Alpha 1-adrenoreceptor antagonist: alfuzosin
- Antianginal: ranolazine
- Anesthetic: meperidine (unless prescribed for short-term use; see **Table 4**)
- Antiarrhythmics: amiodarone, dronedarone, flecainide, propafenone, quinidine
- Antifungals: high doses (>200 mg/day) of ketoconazole or itraconazole, voriconazole (with the exception as described in **Table 4**)
- Anti-gout: colchicine (with the exceptions as described in **Table 4**)
- Antipsychotics: lurasidone, pimozide
- Ergot and Ergot Derivative medications such as dihydroergotamine, dihydroergotamine mesylate, ergotamine, ergotamine tartrate, methylergonovine, methylergonovine maleate, cisapride, flecainide, lovastatin, simvastatin, lomitapide
- GI Motility Agent: cisapride
- Hepatitis C direct acting antivirals: glecaprevir/pibrentasvir, simeprevir
- HIV-1 Protease Inhibitor saquinavir with ritonavir when taken in combination with rifampin (**Table 4**)
- HMG-CoA Reductase Inhibitors: lovastatin, simvastatin
- Long-acting beta-adrenoceptor agonist: salmeterol
- Microsomal triglyceride transfer protein (MTTP) Inhibitor: lomitapide
- PDE5 Inhibitor: sildenafil (Revatio®) when used for the treatment of pulmonary arterial hypertension (PAH)
- PDE5 Inhibitor avanafil
- Sedative/Hypnotics: triazolam, orally administered midazolam

Additional information on observed medication interactions that may require dose modifications of abacavir, lamivudine, and ritonavir are described in **Section 4.10.2**.

4.10.2 Precautionary Use Therapies, Herbal Products, Supplements, Food, and Beverages

Please note that the following information from the USPIs for abacavir+lamivudine and ritonavir serve as guides for the Investigator. If an adverse medication/supplement interaction with 1 or more ART medications is suspected though not specifically listed as such in the USPI, the dose of the suspected interacting medication/supplement should be modified or discontinued first before modifying the dose of abacavir, lamivudine, or ritanovir. If the dose of the suspected interacting medication/supplement cannot be modified or discontinued, then dose modifications and/or discontinuations of abacavir, lamivudine, and/or ritanovir may proceed at the discretion of the Investigator.

Medicines, Dietary Supplements, Foods, and Beverages that Interact with Abacavir+Lamivudine

Potential medication interactions can occur in people taking abacavir+lamivudine. Please ask participants if they are taking any of the following medicines or using the following dietary supplement, so the dose of these medicines/supplements can be adjusted.

- methadone
- sorbitol (an artificial sweetener)
- riociguat

Since ethanol decreases the elimination of abacavir, participants will be advised to limit alcohol consumption while participating in this study.

Medicines and Dietary Supplements that Interact with Ritonavir

Ritonavir also inhibits CYP2D6 to a lesser extent. Co-administration of substrates of CYP2D6 with ritonavir could result in increases (up to 2-fold) in the AUC of the other agent, possibly requiring a proportional dosage reduction. Ritonavir also appears to induce CYP3A, CYP1A2, CYP2C9, CYP2C19, and CYP2B6 as well as other enzymes, including glucuronosyl transferase.

The impact on the PR interval of co-administration of ritonavir with other drugs that prolong the PR interval (including calcium channel blockers, beta-adrenergic blockers, digoxin and atazanavir) has not been evaluated. As a result, co-administration of ritonavir with these drugs should be undertaken with caution, particularly with those drugs metabolized by CYP3A (**Section 7.3.1.7** and **Table 4**). Co-administration of ritonavir with other CYP3A substrates may require a dose adjustment or additional monitoring as described in **Section 7** or **Table 4**.

Treatment with ritonavir therapy alone or in combination with saquinavir has resulted in substantial increases in the concentration of total cholesterol and triglycerides.

When co-administering ritonavir with other protease inhibitors, see the full prescribing information for that protease inhibitor including important Warnings and Precautions.

Table 4 provides a list of established or potentially clinically significant drug interactions. Alteration in dose or regimen may be recommended based on drug interaction studies or predicted interaction (see Contraindications (4), Warnings and Precautions (5.1), and Clinical Pharmacology (12.3) sections of the USPI) for magnitude of interaction. Please note that the examples in **Table 4** are a guide and are not considered a comprehensive list of all possible drugs that may interact with ritonavir. The Investigator should consult appropriate references for comprehensive information.

Table 4: Established and Other Potentially Significant Drug Interactions

**Concomitant Drug Class:**<br>**Drug Name**	**Effect on Concentration of Ritonavir or Concomitant Drug**	**Clinical Comment**
HIV-Antiviral Agents		
HIV-1 Protease Inhibitor:<br>atazanavir<br>darunavir<br>fosamprenavir	↑ amprenavir<br>↑ atazanavir<br>↑ darunavir	See the complete prescribing information for fosamprenavir, atazanavir, darunavir for details on co-administration with ritonavir.
HIV-1 Protease Inhibitor:<br>indinavir	↑ indinavir	Appropriate doses for this combination, with respect to efficacy and safety, have not been established.
HIV-1 Protease Inhibitor:<br>saquinavir	↑ saquinavir	See the complete prescribing information for saquinavir for details on co-administration of saquinavir and ritonavir. Saquinavir/ritonavir in combination with rifampin is not recommended due to the risk of severe hepatotoxicity (presenting as increased hepatic

		transaminases) if the three drugs are given together.
HIV-1 Protease Inhibitor:<br>tipranavir	↑ tipranavir	See the complete prescribing information for tipranavir for details on co-administration of tipranavir and ritonavir.
Non-Nucleoside Reverse Transcriptase Inhibitor:<br>delavirdine	↑ ritonavir	Appropriate doses of this combination with respect to safety and efficacy have not been established.
HIV-1 CCR5 – antagonist: maraviroc	↑ maraviroc	See the complete prescribing information for maraviroc for details on co-administration of maraviroc and ritonavir-containing protease inhibitors.
Integrase Inhibitor:<br>raltegravir	↓ raltegravir	The effects of ritonavir on raltegravir with ritonavir dosage regimens greater than 100 mg twice daily have not been evaluated, however raltegravir concentrations may be decreased with ritonavir coadministration.
Other Agents		
Alpha 1-Adrenoreceptor Antagonist:<br>alfuzosin	↑ alfuzosin	Contraindicated due to potential hypotension (see Contraindications (4) section of the ritonavir USPI) (**Section 4.10.1**).
Antianginal:<br>ranolazine	↑ ranolazine	Contraindicated due to potential for serious and/or life-threatening reactions (see Contraindications (4) of the ritonavir USPI) (**Section 4.10.1**).
Analgesics, Narcotic:<br>tramadol,<br>propoxyphene,<br>methadone,<br>fentanyl	↑ analgesics<br><br>↓ methadone<br><br>↑ fentanyl	A dose decrease may be needed for these drugs when co-administered with ritonavir.<br><br>Dosage increase of methadone may be considered.<br><br>Careful monitoring of therapeutic and adverse effects (including potentially fatal respiratory depression) is recommended when fentanyl is concomitantly administered with ritonavir.
Anesthetic:<br>meperidine	↓ meperidine/↑ normeperidine (metabolite)	Dosage increase and long-term use of meperidine with ritonavir are not recommended due to the increased concentrations of the metabolite normeperidine which has both analgesic activity and CNS stimulant activity (eg, seizures).

Antialcoholics:<br>disulfiram/metronidazole		Ritonavir formulations contain ethanol, which can produce disulfiram-like reactions when co-administered with disulfiram or other drugs that produce this reaction (eg, metronidazole).
Antiarrhythmics:<br>amiodarone,<br>dronedarone,<br>flecainide,<br>propafenone, quinidine	↑ antiarrhythmics	Contraindicated due to potential for cardiac arrhythmias (see Contraindications (4) section of the ritonavir USPI) (**Section 4.10.1**).
Antiarrhythmics:<br>disopyramide,<br>lidocaine, mexiletine	↑ antiarrhythmics	Caution is warranted and therapeutic concentration monitoring is recommended for antiarrhythmics when co-administered with ritonavir, if available.
Anticancer Agents:<br>abemaciclib,<br>apalutamide,<br>dasatinib,<br>encorafenib,<br>ibrutinib,<br>ivosidenib,<br>neratinib,<br>nilotinib,<br>venetoclax,<br>vinblastine,<br>vincristine	↑ anticancer agents<br>↓ ritonavir#	Avoid co-administration of encorafenib or ivosidenib with ritonavir due to potential risk of serious adverse events such as QT interval prolongation. If co-administration of encorafenib with ritonavir cannot be avoided, modify dose as recommended in encorafenib USPI. If co-administration of ivosidenib with ritonavir cannot be avoided, reduce ivosidenib dose to 250 mg once daily.<br><br>Avoid use of neratinib, venetoclax or ibrutinib with ritonavir.<br><br>For vincristine and vinblastine, consideration should be given to temporarily withholding the ritonavir containing antiretroviral regimen in participants who develop significant hematologic or GI side effects when ritonavir is administered concurrently with vincristine or vinblastine.<br><br>A decrease in the dosage or an adjustment of the dosing interval of nilotinib and dasatinib may be necessary for participants requiring co-administration with strong CYP3A inhibitors such as ritonavir. Please refer to the nilotinib and dasatinib prescribing information for dosing instructions.
Anticoagulant:<br>warfarin	↑↓ warfarin	Initial frequent monitoring of the INR during ritonavir and warfarin

		co-administration is recommended.
Anticoagulant:<br>rivaroxaban	↑ rivaroxaban	Avoid concomitant use of rivaroxaban and ritonavir. Co-administration of ritonavir and rivaroxaban may lead to risk of increased bleeding.
Anticonvulsants:<br>carbamazepine,<br>clonazepam,<br>ethosuximide	↑ anticonvulsants	A dose decrease may be needed for these drugs when co-administered with ritonavir and therapeutic concentration monitoring is recommended for these anticonvulsants, if available.
Anticonvulsants:<br>divalproex,<br>lamotrigine, phenytoin	↓ anticonvulsants	A dose increase may be needed for these drugs when co-administered with ritonavir and therapeutic concentration monitoring is recommended for these anticonvulsants, if available.
Antidepressants:<br>nefazodone,<br>SSRIs: eg,<br>fluoxetine,<br>paroxetine,<br>tricyclics: eg,<br>amitriptyline,<br>nortriptyline	↑ antidepressants	A dose decrease may be needed for these drugs when co-administered with ritonavir.
Antidepressant:<br>bupropion	↓ bupropion<br>↓ active metabolite, hydroxybupropion	Participants receiving ritonavir and bupropion concurrently should be monitored for an adequate clinical response to bupropion.
Antidepressant:<br>desipramine	↑ desipramine	Dosage reduction and concentration monitoring of desipramine is recommended.
Antidepressant:<br>trazodone	↑ trazodone	Adverse events of nausea, dizziness, hypotension and syncope have been observed following co-administration of trazodone and ritonavir. A lower dose of trazodone should be considered.
Antiemetic:<br>dronabinol	↑ dronabinol	A dose decrease of dronabinol may be needed when co-administered with ritonavir.
Antifungals:<br>ketoconazole<br>itraconazole<br>voriconazole	↑ ketoconazole<br>↑ itraconazole<br>↓ voriconazole	High doses of ketoconazole or itraconazole (greater than 200 mg per day) are not recommended.<br><br>Co-administration of voriconazole and ritonavir doses of 400 mg every 12 hours or greater is contraindicated due to the potential for loss of antifungal response (see Contraindications

		(4) section of the ritonavir USPI) (**Section 4.10.1**).<br><br>Co-administration of voriconazole and ritonavir 100 mg should be avoided, unless an assessment of the benefit/risk to the patient justifies the use of voriconazole.
Anti-gout:<br>colchicine	↑ colchicine	Contraindicated due to potential for serious and/or life-threatening reactions in participants with renal and/or hepatic impairment (see Contraindications (4) section of the ritonavir USPI) (**Section 4.10.1**).<br><br>For participants with normal renal or hepatic function:<br>*Treatment of gout flares-co-administration of colchicine in participants on ritonavir:* 0.6 mg (1 tablet) for 1 dose, followed by 0.3 mg (half tablet) 1 hour later. Dose to be repeated no earlier than 3 days.<br><br>*Prophylaxis of gout flares-co-administration of colchicine in participants on ritonavir:* If the original colchicine regimen was 0.6 mg twice a day, the regimen should be adjusted to 0.3 mg once a day. If the original colchicine regimen was 0.6 mg once a day, the regimen should be adjusted to 0.3 mg once every other day.<br><br>*Treatment of FMF-co-administration of colchicine in participants on ritonavir:* Maximum daily dose of 0.6 mg (may be given as 0.3 mg twice a day).
Anti-infective:<br>clarithromycin	↑ clarithromycin	For participants with renal impairment, adjust clarithromycin dose as follows:<br>• For participants with CL_{CR} 30 to 60 mL per min the dose of clarithromycin should be reduced by 50%.<br>No dose adjustment for participants with normal renal function is necessary.
Antimycobacterial:<br>bedaquiline	↑ bedaquiline	Bedaquiline should only be used with ritonavir if the benefit of co-administration outweighs the risk.

Antimycobacterial:<br>rifabutin	↑ rifabutin and rifabutin metabolite	Dosage reduction of rifabutin by at least three-quarters of the usual dose of 300 mg per day is recommended (eg, 150 mg every other day or 3 times a week). Further dosage reduction may be necessary.
Antiparasitic:<br>atovaquone	↓ atovaquone	Clinical significance is unknown; however, increase in atovaquone dose may be needed.
Antiparasitic:<br>quinine	↑ quinine	A dose decrease of quinine may be needed when co-administered with ritonavir.
Antipsychotics:<br>lurasidone<br><br><br><br><br>pimozide	↑ lurasidone<br><br><br><br><br>↑ pimozide	Contraindicated due to potential for serious and/or life-threatening reactions (see Contraindications (4) section of the ritonavir USPI) (**Section 4.10.1**).<br><br>Contraindicated due to potential for serious and/or life-threatening reactions such as cardiac arrhythmias (see Contraindications (4) section of the ritonavir USPI) (**Section 4.10.1**).
Antipsychotics:<br>perphenazine,<br>risperidone,<br>thioridazine	↑ antipsychotics	A dose decrease may be needed for these drugs when co-administered with ritonavir.
Antipsychotics:<br>quetiapine	↑ quetiapine	<u>Initiation of ritonavir in participants taking quetiapine:</u><br>Consider alternative ART to avoid increases in quetiapine exposures. If coadministration is necessary, reduce the quetiapine dose to 1/6 of the current dose and monitor for quetiapine-associated adverse reactions. Refer to the quetiapine prescribing information for recommendations on adverse reaction monitoring.<br><br><u>Initiation of quetiapine in participants taking ritonavir:</u><br>Refer to the quetiapine prescribing information for initial dosing and titration of quetiapine.
β-Blockers:<br>metoprolol, timolol	↑ beta-blockers	Caution is warranted and clinical monitoring of participants is recommended. A dose decrease may be needed for these drugs when co-administered with ritonavir.

Bronchodilator:<br>theophylline	↓ theophylline	Increased dosage of theophylline may be required; therapeutic monitoring should be considered.
Calcium channel blockers:<br>diltiazem, nifedipine, verapamil	↑ calcium channel blockers	Caution is warranted and clinical monitoring of participants is recommended. A dose decrease may be needed for these drugs when co-administered with ritonavir.
Digoxin	↑ digoxin	Concomitant administration of ritonavir with digoxin may increase digoxin levels. Caution should be exercised when co-administering ritonavir with digoxin, with appropriate monitoring of serum digoxin levels.
Endothelin receptor antagonists: bosentan	↑ bosentan	<u>Co-administration of bosentan in participants on ritonavir:</u><br>In participants who have been receiving ritonavir for at least 10 days, start bosentan at 62.5 mg once daily or every other day based upon individual tolerability.<br><br><u>Co-administration of ritonavir in participants on bosentan:</u><br>Discontinue use of bosentan at least 36 hours prior to initiation of ritonavir.<br><br>After at least 10 days following the initiation of ritonavir, resume bosentan at 62.5 mg once daily or every other day based upon individual tolerability.
GnRH Receptor Antagonists:<br>elagolix	↑ elagolix<br>↓ ritonavir	Concomitant use of elagolix 200 mg twice daily and ritonavir for more than 1 month is not recommended due to potential risk of adverse events such as bone loss and hepatic transaminase elevations. Limit concomitant use of elagolix 150 mg once daily and ritonavir to 6 months.
Ergot Derivatives:<br>dihydroergotamine, ergotamine, methylergonovine	↑ ergot derivatives	Contraindicated due to potential for acute ergot toxicity characterized by vasospasm and ischemia of the extremities and other tissues including the CNS (see Contraindications (4) section of the ritonavir USPI) (**Section 4.10.1**).

GI Motility Agent:<br>cisapride	↑ cisapride	Contraindicated due to potential for cardiac arrhythmias (see Contraindications (4) section of the ritonavir USPI) (**Section 4.10.1**).
Hepatitis C direct acting antiviral:<br>glecaprevir/pibrentasvir<br><br>simeprevir	↑ glecaprevir<br>↑ pibrentasvir<br><br>↑simeprevir	It is not recommended to co-administer ritonavir with glecaprevir/pibrentasvir, or simeprevir.
Lipid-modifying agents<br>HMG-CoA Reductase Inhibitor:<br>lovastatin<br>simvastatin<br><br>atorvastatin<br>rosuvastatin<br><br>MTTP Inhibitor:<br>lomitapide	↑ lovastatin<br>↑ simvastatin<br><br>↑ atorvastatin<br>↑ rosuvastatin<br><br>↑ lomitapide	Contraindicated due to potential for myopathy including rhabdomyolysis (see Contraindications (4) section of the ritonavir USPI) (**Section 4.10.1**).<br><br>Titrate atorvastatin and rosuvastatin dose carefully and use the lowest necessary dose. If ritonavir is used with another protease inhibitor, see the complete prescribing information for the concomitant protease inhibitor for details on co-administration with atorvastatin and rosuvastatin.<br><br>Lomitapide is a sensitive substrate for CYP3A4 metabolism. CYP3A4 inhibitors increase the exposure of lomitapide, with strong inhibitors increasing exposure approximately 27-fold. Concomitant use of moderate or strong CYP3A4 inhibitors with lomitapide is contraindicated due to potential for hepatotoxicity (see Contraindications (4) section of the ritonavir USPI) (**Section 4.10.1**).
Immunosuppressants:<br>cyclosporine,<br>tacrolimus,<br>sirolimus<br>(rapamycin)	↑ immunosuppressants	Therapeutic concentration monitoring is recommended for immunosuppressant agents when co-administered with ritonavir.
Kinase Inhibitors:<br>fostamatinib (*also see anticancer agents above*)	↑ fostamatinib metabolite R406	Monitor for toxicities of R406 exposure resulting in dose-related adverse events such as hepatotoxicity and neutropenia. Fostamatinib dose reduction may be required.
Long-acting beta-adrenoceptor agonist:<br>salmeterol	↑ salmeterol	Concurrent administration of salmeterol and ritonavir is not recommended. The combination

		may result in increased risk of cardiovascular adverse events associated with salmeterol, including QT prolongation, palpitations and sinus tachycardia.
Oral Contraceptives or Patch Contraceptives:<br>ethinyl estradiol	↓ ethinyl estradiol	Alternate methods of contraception should be considered (**Section 4.11**).
PDE5 Inhibitors:<br>avanafil<br>sildenafil,<br>tadalafil,<br>vardenafil	↑ avanafil<br>↑ sildenafil<br>↑ tadalafil<br>↑ vardenafil	Sildenafil when used for the treatment of PAH (Revatio®) is contraindicated due to the potential for sildenafil-associated adverse events, including visual abnormalities, hypotension, prolonged erection, and syncope (see Contraindications (4) section of the ritonavir USPI) (**Section 4.10.1**).<br><br>Do not use ritonavir with avanafil because a safe and effective avanafil dosage regimen has not been established.<br><br>Particular caution should be used when prescribing sildenafil, tadalafil or vardenafil in participants receiving ritonavir. Coadministration of ritonavir with these drugs may result in an increase in PDE5 inhibitor associated adverse events, including hypotension, syncope, visual changes, and prolonged erection.<br><br>Use of PDE5 inhibitors for PAH: Sildenafil (Revatio®) is contraindicated (see Contraindications (4) section of the ritonavir USPI).<br><br>The following dose adjustments are recommended for use of tadalafil (Adcirca®) with ritonavir:<br><br><u>Co-administration of ADCIRCA in participants on ritonavir:</u><br>In participants receiving ritonavir for at least 1 week, start ADCIRCA at 20 mg once daily. Increase to 40 mg once daily based upon individual tolerability.<br><br><u>Co-administration of ritonavir in participants on ADCIRCA:</u>

		Avoid use of ADCIRCA during the initiation of ritonavir. Stop ADCIRCA at least 24 hours prior to starting ritonavir. After at least 1 week following the initiation of ritonavir, resume ADCIRCA at 20 mg once daily. Increase to 40 mg once daily based upon individual tolerability.<br><br><u>Use of PDE5 inhibitors for the treatment of erectile dysfunction:</u><br><br>It is recommended not to exceed the following doses:<br>• Sildenafil: 25 mg every 48 hours<br>• Tadalafil: 10 mg every 72 hours<br>• Vardenafil: 2.5 mg every 72 hours<br><br>Use with increased monitoring for adverse events.
Sedative/hypnotics:<br>buspirone, clorazepate, diazepam, estazolam, flurazepam, zolpidem	↑ sedative/hypnotics	A dose decrease may be needed for these drugs when co-administered with ritonavir.
Sedative/Hypnotics:<br>triazolam,<br>orally administered midazolam	↑ triazolam<br>↑ midazolam	Contraindicated due to potential for prolonged or increased sedation or respiratory depression (see Contraindications (4) section of the ritonavir USPI) (**Section 4.10.1**).
Sedative/Hypnotics:<br>Parenteral midazolam	↑ midazolam	Co-administration should be done in a setting which ensures close clinical monitoring and appropriate medical management in case of respiratory depression and/or prolonged sedation. Dosage reduction for midazolam should be considered, especially if more than a single dose of midazolam is administered.
Stimulant:<br>methamphetamine	↑ methamphetamine	Use with caution. A dose decrease of methamphetamine may be needed when co-administered with ritonavir.
Systemic/Inhaled/Nasal/Ophthalmic Corticosteroids:<br>eg, betamethasone<br>budesonide<br>ciclesonide<br>dexamethasone<br>fluticasone	↑ glucocorticoids	Coadministration with corticosteroids whose exposures are significantly increased by strong CYP3A inhibitors can increase the risk for Cushing's syndrome and adrenal suppression. Alternative

methylprednisolone<br>mometasone<br>prednisone<br>triamcinolone		corticosteroids including beclomethasone and prednisolone (whose PK and/or PD are less affected by strong CYP3A inhibitors relative to other studied steroids) should be considered, particularly for long-term use.

Abbreviations: ART, antiretroviral therapy; CL_{CR}, creatinine clearance; CNS, central nervous system; CYP3A, cytochrome P450 3A; CYP3A4, cytochrome P450 3A4; FMF, familial Mediterranean fever; GI, gastrointestinal; HIV, human immunodeficiency virus; INR, International Normalized Ratio; MTTP, microsomal triglyceride transfer protein; PAH, pulmonary arterial hypertension; PD, pharmacodynamics: P-gp, permeability glycoprotein; PK, pharmacokinetics; SSRIs, selective serotonin reuptake inhibitors; USPI, United States Package Insert
[#] Refers to interaction with apalutamide.

Medicines and Dietary Supplements that May Interact with Temozolomide

Dose modifications of temozolomide may be required for participants taking a medicine or dietary supplement that contains valproic acid.

Investigators should avoid prescribing the aforementioned medications or dietary supplements for participants while they are receiving study treatment.

4.11 Contraception

Contraception Requirements for Women
The following are acceptable measures of contraception:

- Abstinence (not having sexual relations with a person of the opposite sex)
- Implantable hormone (eg, Norplant[*])
- Intrauterine Device (IUD)
- Male partner has had a vasectomy
- Female sterilization
- Hormonal injection[*]
- Oral contraceptives[*]

[*] Ritonavir may reduce how well hormonal birth control works. Females who may become pregnant should use another effective form of birth control or an additional barrier method of birth control during treatment with ritonavir.

Participants must use contraception from the time of signing the informed consent form, during study treatment, and for at least 6 months after stopping study treatment.

Female participants should not become pregnant or breastfeed for at least 6 months after the last dose of study treatment. Similarly, female partners of male participants should not become pregnant for at least 6 months after the male participant has received the last dose of study treatment.

Participants must not donate an egg for at least 6 months after the last dose of study treatment. If female participants become pregnant while participating in the study or within 6 months of completing study treatment, they should inform their Study Doctor immediately. The Study Doctor may want to follow the pregnancy and may ask the female partner to sign a consent form, so the Study Doctor can collect information about the outcome of the pregnancy.

Contraception Requirements for Men

One of the following forms of contraception should be used by men or their female partner of childbearing potential:

- Abstinence (not having sexual relations with a person of the opposite sex)
- Implantable hormone (eg, Norplant)
- Intrauterine Device (IUD)
- Vasectomy
- Female sterilization
- Hormonal injection
- Oral contraceptives

Male participants must use contraception from the time of signing the informed consent form, during study treatment, and for at least 3 months after stopping study treatment. Participants should also refrain from donating semen during therapy and for at least 3 months after stopping study treatment.

There is theoretical concern that study treatment can result in sperm abnormalities and/or that men can transmit harmful substances in their semen during sex. Therefore, males must remain abstinent or use a condom, even if they have undergone a vasectomy, when engaging in sexual activities.

If a female partner of a male participant becomes pregnant or suspects becoming pregnant during study treatment or within 3 months after the male participant completed study treatment, the Study Doctor must be informed immediately. The Study Doctor may want to follow the pregnancy and may ask the female partner to sign a consent form, so the Study Doctor can collect information about the outcome of the pregnancy.

4.12 Participant Duration and Follow-Up

Participation in the study will last approximately 24 months (2 years). This includes a Screening Period of up to 7 days before LITT, a Treatment Period of up to 12 months (1 year), and a Safety Follow-Up Period of up to 12 months (1 year) or until participant expiration, whichever occurs later. Participants that respond to study treatment may continue to receive ART at the Investigator's discretion (**Section** 4.7).

4.13 Study Duration

With a duration of enrollment of 36 months (3 years) and a per-patient study participation of approximately 24 months (2 years), the total estimated study duration will be approximately 5 years.

4.14 End of Study Definition

The end of study will occur when the last patient enrolled expires or completes the last visit for the Safety Follow-up Period, which is estimated to occur 24 months (2 years) after enrollment.

5 STUDY INTERVENTION DISCONTINUATION AND PARTICIPANT DISCONTINUATION/WITHDRAWAL

5.1 Discontinuation of Study Intervention

Discontinuation from protocol treatment does not mean discontinuation from the study, and remaining study procedures should be completed as indicated by the protocol. If a clinically significant finding is identified (including but not limited to changes from baseline) after enrollment, the Investigator or qualified designee will determine if any change in participant management is needed. Any new clinically relevant finding will be reported as an AE (**Section 11.4**).

The data to be collected at the time of study intervention discontinuation will include the data collected during the end of treatment and follow-up visits.

5.2 Participant Discontinuation/Withdrawal from the Study

Participants are free to withdraw from participation in the study at any time upon request. An Investigator may discontinue or withdraw a participant from the study for the following reasons:

- Pregnancy;
- Significant study intervention non-compliance;
- If any clinical AE (**Section 11.4**), laboratory abnormality (**Section 11.1.1**), or other medical condition or situation occurs such that continued participation in the study would not be in the best interest of the participant;
- Disease progression, which requires discontinuation of the study intervention (**Section 10.2**);
- If the participant meets an exclusion criterion (either newly developed or not previously recognized) that precludes further study participation (**Section 3.2**); or
- Participant unable to receive ART for 3 weeks (**Section 7**).

The reason for participant discontinuation or withdrawal from the study will be recorded on the CRF/eCRF.

5.2.1 Data Retention When Participants Withdraw from FDA-Regulated Clinical Trials

An Investigator may ask a participant who is withdrawing whether the participant wishes to provide continued follow-up and further data collection subsequent to their withdrawal from the interventional portion of the study. Under this circumstance, the discussion with the participant would distinguish between study-related interventions and continued follow-up of associated clinical outcome information, such as medical course or laboratory results obtained through non-invasive chart review, and address the maintenance of privacy and confidentiality of the participant's information.

If a participant withdraws from the interventional portion of the study but agrees to continued follow-up of associated clinical outcome information as described in the previous paragraph, the Investigator must obtain the participant's informed consent for this limited participation in the study (assuming such a situation was not described in the original informed consent form). In accordance with Food and Drug Administration (FDA) regulations, Institutional Review Board (IRB) approval of informed consent documents would be required (21 Code of Federal Regulations [CFR] 50.25, 56.109(b), 312.60, 312.66, 812.100).

5.3 Participant Replacement Criteria

Participants who sign the informed consent form and are enrolled but do not receive the investigational treatment will be replaced.

Participants who sign the informed consent form, receive the protocol treatment, and subsequently withdraw or are withdrawn or discontinued from the study will be replaced.

5.4 Screen Failures

Screen failures are defined as participants who consent to participate in the clinical trial but are not subsequently entered in the study. Screen failures will not be rescreened if they are eligible at a later time.

5.5 Lost to Follow Up

A participant will be considered lost to follow up if he or she fails to return for 3 consecutive visits and is unable to be contacted by the study site staff.

The following actions must be taken if a participant fails to return to the clinic for a required study visit:

- **For Missed Treatment Visits:** Study personnel will attempt to contact the participant and reschedule the missed treatment visit as soon as possible and counsel the participant on the importance of maintaining the assigned visit schedule and ascertain if the participant wishes to and/or should continue in the study.
- **For Missed Post-Treatment Follow-Up Visits:** Study personnel will attempt to contact the participant and reschedule the missed post-treatment follow-up visit within $\leq$14 days and counsel the participant on the importance of maintaining the assigned visit schedule and ascertain if the participant wishes to and/or should continue in the study.
- Before a participant is deemed lost to follow-up, the Investigator or designee will make every effort to regain contact with the participant (where possible, 3 telephone calls and, if necessary, a certified letter to the participant's last known mailing address or local equivalent methods). These contact attempts should be documented in the participant's medical record or study file.
- Should the participant continue to be unreachable, he or she will be considered to have withdrawn from the study with a primary reason of lost to follow up.

6 STUDY AGENTS

6.1 Abacavir+Lamivudine

6.1.1 Description of Product and Mechanism of Action

Abicavir+Lamivudine tablets are a combination of abacavir and lamivudine, which are both nucleoside analogue HIV-1 reverse transcriptase inhibitors, and are indicated in combination with other antiretroviral agents for the treatment of HIV-1 infection. For more information, please refer to the USPI.

6.1.2 Formulation

Abacavir+Lamivudine will be supplied commercially as tablets, which contain 600 mg of abacavir as abacavir sulfate and 300 mg of lamivudine. The tablets are modified capsule-shaped, orange, film-coated, and debossed with "GS FC2" on 1 side with no markings on the reverse side. For more information on the commercially available formulation, please refer to the USPI.

6.1.3 Acquisition and Accountability

Abacavir+Lamivudine will be supplied commercially through the research pharmacy. Participants will receive ART medications free of charge while taking part in this study.

6.1.4 Agent Preparation and Administration

Abacavir+Lamivudine will be prepared per the USPI and institutional standards.

6.1.5 Packaging

The drug product is abacavir+lamivudine and will be supplied as tablets. The tablets are packaged as follows:

- Bottles of 30 tablets (NDC 49702-206-13).

Detailed information on abacavir+lamivudine can be found in the USPI. All study treatment supplies must be stored in accordance with the manufacturer's instructions and package labeling. Until dispensed to the participants, the study treatment will be stored in a securely locked area accessible to authorized personnel only.

6.1.6 Labeling

Upon supply to site, abacavir+lamivudine will be labeled "for investigational use only" as per FDA regulations.

6.1.7 Storage

Abacavir+Lamivudine must be stored in a secure area with access limited to the Investigator and authorized staff and under the physical conditions that are consistent with abacavir+lamivudine-specific requirements.

6.2 Ritonavir

6.2.1 Description of Product and Mechanism of Action

Ritonavir is an antiretroviral drug indicated in combination with other antiretroviral agents for the treatment of HIV-1 infection. For more information, please refer to the USPI.

6.2.2 Formulation

Ritonavir will be supplied commercially as tablets containing 100 mg ritonavir. Ritonavir tablets are white, film-coated ovaloid tablets debossed with the "a" logo and the code NK or debossed with "NK" on 1 side. For more information on the commercially available formulation, please refer to the USPI.

6.2.3 Acquisition and Accountability

Ritonavir will be supplied commercially through the research pharmacy. Participants will receive ART medications free of charge while taking part in this study.

6.2.4 Agent Preparation and Administration

Ritonavir will be prepared per the USPI and institutional standards.

6.2.5 Packaging

The drug product is ritonavir and will be supplied as tablets. The tablets are packaged as follows:

- Bottles of 30 tablets (NDC 0074-3333-30).

Detailed information on ritonavir can be found in the USPI. All study treatment supplies must be stored in accordance with the manufacturer's instructions and package labeling. Until dispensed to the participants, the study treatment will be stored in a securely locked area accessible to authorized personnel only.

6.2.6 Labeling

Upon supply to site, ritonavir will be labeled "for investigational use only" as per FDA regulations.

6.2.7 Storage

Ritonavir must be stored in a secure area with access limited to the Investigator and authorized staff and under the physical conditions that are consistent with ritonavir-specific requirements.

6.3 Temozolomide

6.3.1 Description of Product and Mechanism of Action

Temozolomide is indicated for the treatment of adult patients with newly-diagnosed glioblastoma concomitantly with radiotherapy and then as maintenance treatment. Temozolomide is also indicated for the treatment of adult patients with refractory anaplastic astrocytoma who have experienced disease progression on a drug regimen containing nitrosourea and procarbazine.

Temozolomide is an alkylating drug. For more information, please refer to the USPI.

6.3.2 Formulation

Temozolomide will be supplied commercially. For more information on the commercially available formulation, please refer to the USPI.

6.3.3 Acquisition and Accountability

Temozolomide will be supplied commercially.

6.3.4 Agent Preparation and Administration

Temozolomide will be prepared per the USPI and institutional standards.

6.3.5 Packaging

The drug product is temozolomide and will be supplied as capsules.

Detailed information on temozolomide can be found in the USPI. All study treatment supplies must be stored in accordance with the manufacturer's instructions and package labeling. Until dispensed to the participants, the study treatment will be stored in a securely locked area accessible to authorized personnel only.

6.3.6 Labeling

Temozolomide will be supplied from the commercial stock and will not be re-labeled.

6.3.7 Storage

Temozolomide must be stored in a secure area with access limited to the Investigator and authorized staff and under the physical conditions that are consistent with temozolomide-specific requirements.

7 TREATMENT DISCONTINUATION AND ADVERSE EVENT MANAGEMENT

7.1 Dose Interruption and Delays

7.1.1 Antiretroviral Therapy (ART)

ART will begin at POD7 (**Sections 4.4.4** and **4.5**). However, ART may begin later if the participant is considered unable to begin treatment due to their postoperative condition, ie, inability to swallow medications.

In the event of toxicity development as described in **Table 5**, the cycle period for ART medications may be decreased from 7 days to 5 days if an alternate cause for toxicity is not found. Please also refer to **Table 5** for permitted dose holds and modifications of ART component medications.

7.1.2 Adjuvant Treatment

Radiotherapy and temozolomide will begin 2 weeks (±7 days) after surgery, provided the participant has sufficiently recovered from surgery (**Table 2** and **Section 4.4.5**). Temozolomide treatment will be administered concurrently with radiotherapy at a dose of 75 mg/m² as described in **Section 4.5.6.2**. Following completion of radiotherapy and the 6-week (42-day) regimen of 75 mg/m² temozolomide, participants will have a 4-week (±2 week) rest period before starting adjuvant temozolomide treatment. To accommodate this delay in adjuvant temozolomide treatment start, the timing of follow-up assessments will be shifted accordingly. For permitted dose holds and modifications of temozolomide in the event of toxicity development or other AEs, please refer to **Table 5**.

7.2 Dose Modifications or Discontinuation

Permitted dose modifications or adjustments for ART components and TMZ are described in **Table 5**.

Table 5: Safety Criteria for Adjustment or Stopping of Doses of ART and/or TMZ

Toxicity Considered Related to ART	Dose Modification and/or Action
Gastrointestinal toxicity (eg, nausea, vomiting, diarrhea, GI bleeding)	• In the case of nausea or vomiting, participants should be instructed to take the tablets over a 15-minute period of time, and an anti-emetic should be prescribed if clinically indicated.<br>• In the case of diarrhea, an anti-diarrheal agent should be prescribed if clinically indicated.<br>• In the case of GI bleeding, participants should be started on a proton pump inhibitor and observed until cessation of GI bleeding. If bleeding persists for 4 weeks after ART, ritonavir can be stopped.<br>• If GI toxicity persists, ART may be decreased from 7 to 5 days if an alternate cause for toxicity is not found (**Section 7.1.1**).<br>• Adjust concomitant medications which may be contributing (eg, anti-seizure drugs or TMZ or steroids)

Toxicity Considered Related to ART	Dose Modification and/or Action
≥ Grade 3 non-hematologic toxicity (eg, severe skin reaction, etc.)	• If the event is attributable to ART, hold dosing of ART until the abnormal values return to the levels as specified in the Inclusion/Exclusion Criteria (**Sections** 3.1 and 3.2).<br>• The duration of ART may be decreased from 7 to 5 days each cycle if an alternate cause for toxicity is not found (**Section** 7.1.1).<br>• Adjust concomitant medications which may be contributing (eg, anti-seizure drugs or TMZ)<br>• If an allergic reaction/hypersensitivity reaction is noted, such as SJS or TEN, either abacavir or ritonavir will be discontinued, depending on the suspected cause of the reaction (**Sections** 7.3.1.2, 7.3.1.9, and 7.3.1.10).*<br>• If a diagnosis of pancreatitis is confirmed, ritonavir therapy should be discontinued (**Section** 7.3.1.5).
Acute cardiotoxic event or renal failure	Acute cardiotoxic event:<br>• Ensure that no other cause is found for the abnormality, including other drugs known to cause cardiotoxicity.<br>• If no other cause is found, **de-escalate ritonavir** (**Table 3**).<br>Renal failure:<br>• In the event of renal failure, initiation of dialysis should be considered if participant is refractory to institutional supportive care measures.<br>• Ensure that no other cause is found for the abnormality, including other drugs known to cause renal dysfunction/failure.<br>• If no other cause is found, **de-escalate ritonavir** (**Table 3**).
ALT ≥ 2.5 times ULN and bilirubin ≥ 2.5 times ULN in absence of cholestasis or any other apparent cause	• Ensure that no other cause is found for the abnormality, including other drugs known to cause liver enzyme elevation.<br>• If no other cause is found, **de-escalate ritonavir** (**Table 3**).<br>• Follow the participant with repeat chemistry testing (**Sections** 10.1.6.3 and 10.1.6.4) every 2–3 days until recovery.
≥ Grade 3 hematologic toxicity (eg, anemia, leucopenia, thrombocytopenia)	• Hold TMZ if clinically indicated until abnormal value returns to levels specified in the Inclusion/Exclusion Criteria (**Sections** 3.1 and 3.2).<br>• If no other cause is found and TMZ is held, ritonavir dosing should be de-escalated as described in **Table 3** and **Sections** 4.5.2.1 and 4.5.5.<br>• If ritonavir dose is de-escalated and hematological toxicity continues, adjust lamivudine dose as described in **Section** 7.3.1.1.*

Abbreviations: ALT, alanine transaminase; ART, antiretroviral therapy; GFR, glomerular filtration rate; GI, gastrointestinal; SJS, Stevens-Johnson syndrome; TEN, toxic epidermal necrolysis; TMZ, temozolomide; ULN, upper limit of normal

* If dose adjustment or discontinuation is indicated for either lamivudine or abacavir, the abacavir and lamivudine combination product (abacavir+lamivudine) will be discontinued, and the individual components used to construct the treatment regimen. Ritonavir will continue at the same dose and frequency with no modifications.

7.3 Adverse Event Monitoring and Management

7.3.1 Antiretroviral Therapy

7.3.1.1 Hematological Toxicity

For ART, higher lamivudine exposures were associated with higher rates of hematologic toxicities (neutropenia and anemia), although discontinuations due to neutropenia or anemia each occurred in <1% of patients. Patients with a sustained creatinine clearance between 30 and 49 mL per min who receive abacavir+lamivudine should be monitored for hematologic toxicities. If new or worsening neutropenia or anemia develop and temozolomide (TMZ) is not the cause, lamivudine will be adjusted per lamivudine prescribing information. If lamivudine dose adjustment is indicated, abacavir+lamivudine will be discontinued, and the individual components will be used to construct the treatment regimen. Ritonavir will continue at the same dose and frequency with no modifications.

Toxicity may also be more common in participants with previous exposure to radiation and alkylating agents. The hematological status of all participants will be monitored during treatment (**Sections 8**, **10.1.6.1**, and **10.1.6.2**).

7.3.1.2 Abacavir Hypersensitivity Syndrome

In clinical trials, serious and sometimes fatal hypersensitivity reactions have occurred with abacavir, a component of the combination product abacavir+lamivudine. These reactions have been characterized by 2 or more of the following signs or symptoms: (1) fever; (2) rash; (3) gastrointestinal (GI) symptoms (including nausea, vomiting diarrhea, or abdominal pain); (4) constitutional symptoms (including generalized malaise, fatigue, or achiness); (5) respiratory symptoms (including dyspnea, cough, or pharyngitis). Almost all abacavir hypersensitivity reactions include fever and/or rash as part of the syndrome. If a hypersensitivity reaction is noted, either abacavir or ritonavir will be discontinued, depending on the suspected cause of the reaction (**Table 5** and **Sections 7.3.1.9** and **7.3.1.10**). If abacavir is the suspected cause of the reaction, abacavir+lamivudine will be discontinued, and the participant started on lamivudine. Ritonavir will continue at the same dose and frequency with no modifications.

7.3.1.3 Hepatic Toxicity

Hepatotoxicity has been reported in patients treated with abacavir+lamivudine and ritonavir. The adequacy of hepatic function will be confirmed for all potential participants prior to administration of ART and will be monitored during treatment (**Sections 8** and **10.1.6.4**). Refer to **Table 5** for recommendations on ritonavir dose adjustment for participants who develop hepatic toxicity during the course of treatment.

7.3.1.4 Laboratory Abnormalities

Laboratory abnormalities observed in clinical trials of abacavir were anemia, neutropenia, liver function test abnormalities, and elevations of creatinine phosphokinase (CPK), blood glucose, and triglycerides. Additional laboratory abnormalities observed in clinical trials of lamivudine were thrombocytopenia and elevated levels of bilirubin, amylase, and lipase. In the expanded access program for abacavir, pancreatitis and increased gamma-glutamyl transferase (GGT) were noted. Any identified laboratory abnormalities noted (**Table 5** and **Sections 10.1.6** and **11.1.1**) will be managed according to institutional standards.

7.3.1.5 Pancreatitis

Pancreatitis has been observed in patients receiving ritonavir therapy, including those who developed hypertriglyceridemia. In some cases, fatalities have been observed. Patients with advanced HIV disease may be at increased risk of elevated triglycerides and pancreatitis. Pancreatitis should be considered if clinical symptoms (nausea, vomiting, abdominal pain) or abnormalities in laboratory values (such as increased serum lipase or amylase values) suggestive of pancreatitis should occur. Patients who exhibit these signs or symptoms should be evaluated, and ritonavir therapy should be discontinued if a diagnosis of pancreatitis is made (**Table 5** and **Sections 10.1.6.3** and **10.1.6.4**).

7.3.1.6 Myocardial Infarction

Several prospective, observational, epidemiological studies have reported an association with the use of abacavir and the risk of myocardial infarction (MI). Meta-analyses of randomized, controlled clinical trials have observed no excess risk of MI in abacavir-treated participants as compared with control participants. To date, there is no established biological mechanism to explain the potential increase in risk. In totality, the available data from the observational studies and from controlled clinical trials show inconsistency; therefore, evidence for a causal relationship between abacavir treatment and the risk of MI is inconclusive.

As a precaution, the underlying risk of coronary heart disease should be considered when prescribing antiretroviral therapies, including abacavir, and action taken to minimize all modifiable risk factors (eg, hypertension, hyperlipidemia, diabetes mellitus, smoking) (**Sections 3.1** and **10.1.8**).

7.3.1.7 PR Interval Prolongation

Ritonavir prolongs the PR interval in some patients. Post marketing cases of second- or third-degree atrioventricular block have been reported in patients.

The impact on the PR interval of co-administration of ritonavir with other drugs that prolong the PR interval (including calcium channel blockers, beta-adrenergic blockers, digoxin and atazanavir) has not been evaluated. As a result, co-administration of ritonavir with these drugs should be undertaken with caution, particularly with those drugs metabolized by CYP3A (**Section 4.10.2**). Clinical monitoring is recommended (**Sections 8** and **10.1.8**).

7.3.1.8 Immunogenicity

Adverse events (AEs) will be assessed on an ongoing basis for relationship to immune function and the potential immunologic effects of treatment with ART and temozolomide (**Section 10.1.10**).

7.3.1.9 Skin Reactions

In the post-marketing setting, suspected Stevens-Johnson syndrome (SJS) and toxic epidermal necrolysis (TEN) have been reported in patients receiving abacavir primarily in combination with medications known to be associated with SJS and TEN, respectively. Because of the overlap of clinical signs and symptoms between hypersensitivity to abacavir and SJS and TEN, and the possibility of multiple drug sensitivities in some patients, abacavir should be discontinued and not restarted in such cases. There have also been reports of erythema multiforme with abacavir use. If abacavir is the suspected cause of the reaction, abacavir+lamivudine will be discontinued, and the participant started on lamivudine. Ritonavir will continue at the same dose and frequency with no modifications.

7.3.1.10 Allergic Reactions/Hypersensitivity

Allergic reactions including urticaria, mild skin eruptions, bronchospasm, and angioedema have been reported in patients taking ritonavir. Cases of anaphylaxis, TEN, and SJS have also been reported. Discontinue treatment if severe reactions develop.

7.3.1.11 **Diabetes Mellitus/Hyperglycemia**

New onset diabetes mellitus, exacerbation of pre-existing diabetes mellitus, and hyperglycemia have been reported during postmarketing surveillance in HIV-infected patients receiving protease inhibitor therapy. Some patients required either initiation or dose adjustments of insulin or oral hypoglycemic agents for treatment of these events. In some cases, diabetic ketoacidosis has occurred. In those patients who discontinued protease inhibitor therapy, hyperglycemia persisted in some cases. Because these events have been reported voluntarily during clinical practice, estimates of frequency cannot be made and a causal relationship between protease inhibitor therapy and these events has not been established.

Consider monitoring for hyperglycemia, new onset diabetes mellitus, or an exacerbation of diabetes mellitus in patients treated with ritonavir (**Sections 8** and **10.1.6.3**).

7.3.1.12 **Fat Redistribution**

Redistribution/accumulation of body fat including central obesity, dorsocervical fat enlargement (buffalo hump), peripheral wasting, facial wasting, breast enlargement, and "cushingoid appearance" have been observed in patients receiving antiretroviral therapy. The mechanism and long-term consequences of these events are currently unknown. A causal relationship has not been established.

7.3.1.13 **Patients with Hemophilia**

There have been reports of increased bleeding, including spontaneous skin hematomas and hemarthrosis, in patients with hemophilia type A and B treated with protease inhibitors. In some patients, additional factor VIII was given. In more than half of the reported cases, treatment with protease inhibitors was continued or reintroduced. A causal relationship between protease inhibitor therapy and these events has not been established.

7.3.2 Laser Interstitial Thermal Therapy

Participants will be monitored in the inpatient setting post-LITT procedure as described in **Section 4.4.3**.

The most commonly reported adverse reactions of LITT are headaches. In rare cases, severe adverse reactions including intracranial hemorrhage, stroke, symptomatic cerebral edema, coma, weakness on one or both sides of the body, or sensory/vision loss can occur. During the procedure, the Investigator or Investigator's designee will be treating participants with steroids to mitigate these side effects (**Section 4.8**).

Additional supportive care measures will be provided in accordance with institutional standards (**Section 4.8**).

7.3.3 Radiotherapy

Supportive care measures will be provided in accordance with institutional standards (**Section 4.8**).

7.3.4 Temozolomide

7.3.4.1 **Hematological Toxicity/Myelosuppression**

Bone marrow suppression has been reported during treatment with temozolomide. In isolated cases, bone marrow toxicity may be irreversible and could lead to death in patients with pre-existing immune-suppression. Typically, bone marrow suppression is more common with longer durations and higher doses of temozolomide.

Myelosuppression, including pancytopenia, leukopenia and anemia, some with fatal outcomes, have occurred with temozolomide. Geriatric patients and women have been shown in clinical trials to have a higher risk of developing myelosuppression.

Prior to dosing, patients must have an ANC of 1.5 x 10^9 cells/L or greater and a platelet count of 100 x 10^9 platelets/L or greater (**Section 3.1**).

For the concomitant phase with radiotherapy, obtain a complete blood count (CBC) prior to initiation of treatment and weekly during treatment.

For the 28-day treatment cycles, obtain a CBC prior to treatment on Day 1 and on Day 22 of each cycle (**Section 10.1.6.1**). Perform CBCs weekly until recovery if the ANC falls below 1.5 x 10^9 cells/L and the platelet count falls below 100 x 10^9 platelets/L.

For more information on laboratory measurement of blood cell populations and frequency of testing for CBC with differential during the study, please see **Sections 10.1.6.1** and **8**, respectively.

7.3.4.2 Myelodysplastic Syndrome and Secondary Malignancies

Cases of myelodysplastic syndrome and secondary malignancies, including myeloid leukemia, have been observed following temozolomide administration.

7.3.4.3 *Pneumocystis* Pneumonia

Pneumocystis pneumonia (PCP) can occur in patients receiving temozolomide. The risk of PCP is increased in patients receiving steroids or with longer treatment regimens.

For patients with newly-diagnosed glioblastoma, provide PCP prophylaxis for all patients during the concomitant phase (**Section 4.8**). Continue in patients who experience lymphopenia until resolution to grade 1 or less (**Section 4.8**).

Monitor all patients receiving temozolomide for the development of lymphopenia and PCP (**Section 10.1.6**).

7.3.4.4 Hepatotoxicity

Fatal and severe hepatotoxicity have been reported in patients receiving temozolomide. Patients will be monitored for hepatotoxicity according to institutional standards.

For more information on laboratory measurement of liver function during the study, please see **Sections 10.1.6.4** and **8**, respectively.

7.3.4.5 Embryo-fetal Toxicity and Effects on Fertility

Based on findings from animal studies and its mechanism of action, temozolomide can cause fetal harm when administered to a pregnant woman. Adverse developmental outcomes have been reported in both pregnant patients and pregnant partners of male patients. Oral administration of temozolomide to rats and rabbits during the period of organogenesis resulted in embryo lethality and poly malformations at doses less than the maximum human dose based on body surface area.

Investigators or study personnel should advise females of reproductive potential to use effective contraception as described in **Section 4.11**.

In addition to the contraception requirements as described in **Section 4.11**, Investigators or study personnel should advise male participants of reproductive potential that temozolomide may impair fertility.

8 SCHEDULE OF ASSESSMENTS

Study Procedures	Screening*	POD-1**	POD0***	POD1–2****	POD7†	POD14±7 days	POD28±3 days	POD86±2 days	POD180±2 days	POD365±2 days/EOS‡	Safety Follow-up**, **
Administrative Activities											
Informed Consent (15.1)	X										
Inclusion/Exclusion Criteria (3.1 & 3.2)	X										
Demographics (Error! Reference source not found.)	X										
Medical History (10.1.1)	X										
Clinical Activities											
Concomitant Medications (10.1.2)	X	X		X	X	X	X	X	X	X	X
Physical Exam[1] (10.1.3)	X	X	X	X	X	X	X	X	X	X	X
Height (10.1.3)	X										
Weight (10.1.3)	X										
Vital Signs (10.1.4)	X	X	X	X		X	X	X	X	X	X
Karnofsky Performance Status (10.1.5)	X	X	X	X		X	X	X	X	X	X
ECG (10.1.8)		X									
Clinical Laboratory Assessments											
CBC with Differential[1] (10.1.6.1)	X	X		X	X	X[3]	X[3]	X[3]	X[3]	X	X
Coagulation Studies[1] (10.1.6.2)	X	X									
Blood Chemistry[1] (10.1.6.3)	X	X		X	X	X[3]	X[3]	X[3]	X[3]	X	X
Organ Function Tests[1] (10.1.6.4)	X			X (POD2)	X	X	X	X	X	X	X
Thyroid Tests[1] (10.1.6.5)	X										
Viral Testing (10.1.6.6)	X										
HLA-B Testing (10.1.6.7)	X										
Pregnancy Testing (10.1.6.8)	X	WOCBP: Monthly (±2 weeks) and as clinically indicated									
Urinalysis (10.1.6.9)	X										
Imaging											

Study Procedures	Screening*	POD-1**	POD0***	POD1–2****	POD7†	POD14±7 days	POD28±3 days	POD86±2 days	POD180±2 days	POD365±2 days/EOS‡	Safety Follow-up⁑, ⁂
MRI[1,8] (10.1.7)	X	X	X[2]					X	X	X	X
Study Treatment											
LITT (4.4.2)			X								
ART (4.4.4)					X	X	X	X	X	X[4]	X[4]
Adjuvant Therapy (4.4.5)						X	X	X	X		
Correlative Studies											
Stereotactic Needle Biopsy & CSF[7] (10.1.9 & 0)			X								
Blood Sample Collection[1,5] (0)	X		X	X		X	X	X	X	X	X[6]
Monitoring											
Medication Diary (4.5)					X	X	X	X	X	X	
AE Review & Evaluation (10.1.10)		X	X	X	X	X	X	X	X	X	X
Survival Assessment (10.4.2)											X

Abbreviations: AE, adverse event; ART, antiretroviral therapy; BMP, basic metabolic panel; CBC, complete blood count; CSF, cerebrospinal fluid; ECG, electrocardiogram; EOS, End of Study; HLA-B, Human Leukocyte Antigen-B; LFTs, liver function tests; LITT, laser interstitial thermal therapy; MRI, magnetic resonance imaging; POD, Post-operative Day; WOCBP, women of childbearing potential

* Screening assessments are to be conducted up to 7 days before initiating the study unless otherwise specified. Please note that the Screening Visit and POD-1 Visit may be the same visit.

** Assessments will be performed on patient hospital admission.

*** Refer to **Section 4.4.1** for a listing of pre- and perioperative activities.

**** Participants will be discharged from the hospital at this visit (**Section 4.4.3**).

† Participants will be seen by a neuro-oncologist prior to receiving their first dose of ART (**Section 4.4.4**).

‡ Participants with confirmed disease progression before POD365±2 days will undergo the same tests and procedures as participants with continued treatment response at POD365±2 days.

⁑ Participants will be followed up for ≤1 year after adjuvant treatment or until participant expiration, whichever occurs later.

⁂ If a participant decides to stop taking part in the study early, the participant will be asked whether the Investigator or a member of the study team may continue to collect data on the participant's overall health after study participation ends (**Section 5.2.1**).

1 Tests and procedures to be performed in the event of suspected disease progression.

2 Brain MRI with and without contrast to occur 6 hours after surgery.

3 In accordance with institutional standards, participants receiving temozolomide will undergo weekly (±3 days) blood draws for CMP and/or BMP analysis. Participants receiving temozolomide will also undergo blood draws for CBC with differential analysis prior to temozolomide treatment on Day 1

and Day 22 of each 28-day cycle of temozolomide unless their ANC falls below 1.5 x 10^9 cells/L and the platelet count falls below 100 x 10^9 platelets/L in which case the frequency of blood draws will occur weekly (±3 days) until recovery (**Section 7.3.4.1**).
[4] Participants with treatment response at or beyond POD365±2 days may continue to receive ART as described in **Section 4.7**.
[5] Blood samples for correlative studies will be collected before and after the LITT procedure as well as before and after the tumor biopsy procedure (if performed) in the event of suspected or confirmed disease progression.
[6] During the Safety Follow-up Period, blood samples for research activities will be collected one time every 6 to 8 weeks at the same time as standard of care blood draws.
[7] If disease progression is suspected or confirmed, the participant may undergo a subsequent stereotactic needle biopsy and CSF collection in accordance with standard of care procedures. If this event occurs, a portion of these samples will be saved for future correlative studies as described in **Section 0**.
[8] As described in **Section 10.3**, radiographic response assessments will be performed at six months and one year after LITT and/or at the timing of the best radiographic response.

9 CORRELATIVE STUDIES

Correlative Studies, Blood:

Blood will be obtained at screening using cell preparation tube (CPT)/lymphoprep tubes and will be stored as plasma and buffy coat. Blood will also be collected before and after the LITT procedure as well as before and after the tumor biopsy procedure (if performed) in the event of suspected or confirmed disease progression.

Blood sample analysis will consist of whole blood centrifuging and isolation of peripheral blood mononuclear cells (PBMCs) and characterization of lymphocytes using single-nucleus ribonucleic acid (snRNA)-sequencing and multiplex flow cytometry.

Solid Tumors and CSF:

Tumor specimen(s) will be collected via stereotactic needle biopsy and CSF collected as described in **Section 10.1.9**. Sample(s) that are set aside for correlative research activities will be flash frozen and stored at -20°C. Samples will be analyzed as described in the Laboratory Manual.

Sample Storage and Analysis

Sample processing, storage, and analysis will be conducted at the location(s) as described in the Laboratory Manual.

10 EVALUATION CRITERIA

10.1 Safety Assessments

Participants will undergo safety assessments at the frequency of visits as described in **Section 8**. Please note that additional assessments may be ordered at the Investigator's discretion if clinically indicated. Study team members will be expected to complete the CRF/eCRF during each visit.

10.1.1 Medical History

A complete medical history, including previous history of radiation therapy, chemotherapy, immunotherapy, and/or other antineoplastic treatments as well as history of allergies/allergic reactions will be collected from each participant at the Screening Visit. At the Screening Visit, demographic information will also be collected as described in **Section 14.4.1**.

10.1.2 Concomitant Medications

Participants will be asked to report all prescription and over-the-counter medications as well as dietary supplements that they are currently taking or have recently taken at the Screening Visit and throughout the study at the frequency of visits as described in **Section 8**. NOTE: patients taking dofetilide must be excluded from study participation as described in **Section 3.2**.

10.1.3 Physical Examinations

A complete physical examination performed at Screening and at the end of study or at the time of suspected disease progression will include an examination of all body systems versus a symptom-directed physical examination, which will only involve examination of the affected systems (**Section 8**).

Height and weight will be measured at the frequency of visits as described in **Section 8**.

10.1.4 Vital Signs

Vital signs to be collected during this study are blood pressure, heart rate, temperature (oral or axillary), and oxygen saturation. Vital signs will be collected at the frequency of visits as described in **Section 8**.

10.1.5 Performance Status

Performance status will be measured using the Karnofsky score as provided in APPENDIX A and at the frequency of visits as described in **Section 8**.

10.1.6 Safety Laboratory Analysis

The following sections outline the laboratory analyses to be performed at Screening and at the frequency of visits as described in **Section 8**.

10.1.6.1 **Blood Cell Counts**

Participants will provide blood samples for CBC with differential analysis at the Screening Visit and at the frequency of visits as described in **Section 8**. As per the temozolomide USPI, participants will undergo blood draws for CBCs during adjuvant treatment as described in **Section 7.3.4.1**.

10.1.6.2 **Coagulation Studies**

The following laboratory tests will be performed for coagulation analysis at the Screening Visit and at the frequency of visits as described in **Section 8**:

- Prothrombin time (PT)/International Normalized Ratio (INR)
- Partial thromboplastin time (PTT)

10.1.6.3 Blood Chemistry

Participants will provide blood samples for blood chemistry analysis (ie, the comprehensive metabolic panel [CMP] and/or the basic metabolic panel [BMP]) at the Screening Visit and at the frequency of visits as described in **Section 8**.

10.1.6.4 Organ Function Tests

Participants will provide blood samples for organ function testing at the Screening Visit and at the frequency of visits as described in **Section 8**. Laboratory tests to be performed will include the following.

- Liver Function Tests (LFT): AST and ALT levels
- Lactate dehydrogenase (LDH)
- Amylase
- Lipase
- C-reactive protein (CRP)
- Troponin T

10.1.6.5 Thyroid Tests

The following hormones will be analyzed for thyroid testing at the Screening Visit and at the frequency of visits as described in **Section 8**.

- Triiodothyronine (T3)
- Free thyroxine (T4)
- Thyroid-stimulating hormone (TSH)

10.1.6.6 Viral Testing

Viral testing will be performed at the Screening Visit and as clinically indicated (**Section 8**).

- Hepatitis panel (Hepatitis B [Hep B] surface antigen, Hep B core antibody, and surface antibody, Hepatitis C [Hep C] polymerase chain reaction [PCR]-ribonucleic acid [RNA] analysis of viral load).
- Human immunodeficiency virus (HIV) test

10.1.6.7 Human Leukocyte Antigen-B Testing

Because patients with the HLA-B*5701 metabolize abacavir differently than patients without this variant, participants will provide blood samples for HLA-B*5701 testing prior to receiving ART to identify participants at increased risk for abacavir-related AEs (**Section 7.3.1.2**). NOTE: participants with a pending HLA-B test result may still undergo the LITT procedure as part of their standard medical care. However, the HLA-B test result must be obtained prior to a participant starting ART as part of this study. Participants with the HLA-B*5701 variant must be excluded from study participation as described in **Section 3.2**; however, these participants will continue to receive standard of care treatment, which includes radiotherapy and chemotherapy, as per the treating neuro-oncologist.

10.1.6.8 Pregnancy Testing

Women of childbearing potential (WOCBP) are required to undergo pregnancy testing prior to study participation and at the frequency of visits as described in **Section 8** and as clinically indicated during the study. Blood or urine samples will be collected for analysis of beta-human chorionic gonadotropin levels in this study.

10.1.6.9 Urinalysis

Urinalysis will include gross and macroscopic examination of urine from samples collected at the frequency of visits as described in **Section 8**.

10.1.7 Imaging

Magnetic resonance imaging (MRI) of the brain will be performed with and without contrast at the Screening Visit and at the frequency of visits as described in **Section 8**. Please note that MRIs may also be performed in accordance with standard of care procedures at a greater frequency than what is listed in the Study Calendar at the discretion of the treating physician. If disease progression is suspected, a brain MRI with and without contrast is to be performed as described in **Section 10.3**.

10.1.8 Electrocardiogram

Participants will undergo a 12-lead ECG one day prior to LITT as listed in the Schedule of Assessments (**Section 8**). The 12-lead ECG will be performed according to institutional standards.

If a participant experiences cardiovascular symptoms associated with PR alteration (**Section 7.3.1.7**), serial ECGs will be performed during treatment at the discretion of the Investigator.

10.1.9 Stereotactic Needle Biopsy and Cerebrospinal Fluid Collection

Participants will undergo a stereotactic needle biopsy for pathological analysis and confirmation of HGG diagnosis prior to the LITT procedure according to institutional standards (**Section 4.4.1**). A portion of this biopsy will be saved for future correlative studies as described in **Section 0**. The neuropathologist will review samples from biopsy and dispense tissue for research only after satisfactory tissue is obtained for tumor diagnosis.

If disease progression is suspected or confirmed, the participant may undergo a subsequent stereotactic needle biopsy per standard of care procedures. If this event occurs, a portion of this biopsy will be saved for future correlative studies as described in **Section 0**.

Cerebrospinal fluid (CSF) via lumbar puncture will be collected in accordance with institutional standards at the same time points as described for the stereotactic needle biopsy (**Section 8**).

10.1.10 Adverse Events

Adverse events (AEs) will be collected as described in **Sections 11.3.4** and **11.4** at the frequency of visits as described in **Section 8**.

Adverse events (AEs) will be classified using NCI CTCAE v5.0, recorded, and reported as described in **Section 11**. Disease symptoms noted at Screening will be considered baseline measurements and not reported as an AE as described in **Section 11.3.4**.

10.2 Response Evaluations

Disease response will be evaluated using the Response Assessment in Neuro-Oncology (RANO) criteria as described in **Table 6** (Leao et al, 2019).

Table 6: Criteria for Response Assessment Incorporating MRI and Clinical Factors

Criterion	CR	PR	SD	PD
T1-Gd +	None	≥50% ↓	<50% ↓ to <25% ↑	≥ 25% ↑†
T2/FLAIR	Stable or ↓	Stable or ↓	Stable or ↓	↑†
New lesion	None	None	None	Present†

Corticosteroids	None	Stable or ↓	Stable or ↓	NA[‡]
Clinical status	Stable or ↑	Stable or ↑	Stable or ↑	↓[†]
Requirement for response	All	All	All	Any[‡]
Summary of HGG response criteria	Requires all of the following: Complete disappearance of all enhancing measurable and nonmeasurable disease sustained for at least 4 weeks; no new lesions; stable or improved nonenhancing (T2/FLAIR) lesions; patients must be off corticosteroids (or on physiologic replacement doses only); and stable or improved clinically.<br><br>Note: Patients with nonmeasurable disease only cannot have achieved CR; the best response possible is SD.	Requires all of the following: ≥50% decrease compared with baseline in the sum of products of perpendicular diameters of all measurable enhancing lesions sustained for at least 4 weeks; no progression of nonmeasurable disease; no new lesions; stable or improved nonenhancing (T2/FLAIR) lesions on same or lower dose of corticosteroids compared with baseline scan; the corticosteroid dose at the time of scan evaluation should be no greater than the dose at time of baseline scan; and stable or improved clinically.	Requires all of the following: Does not qualify for CR, PR, or progression; stable nonenhancing (T2/FLAIR) lesions on the same or lower dose of corticosteroids compared with baseline scan. In the event that the corticosteroid dose was increased for new symptoms and signs without confirmation of disease progression on neuroimaging, and subsequent follow-up imaging shows that this increase in corticosteroids was required because of disease progression, the last scan considered to show SD will be the scan obtained when the corticosteroid dose was equivalent to the baseline dose.	Defined by any of the following: ≥25% increase in the sum of the products of perpendicular diameters of enhancing lesions compared with the smallest tumor measurement obtained either at baseline (if no decrease) or best response on stable or increasing doses of corticosteroids[†]; significant increase in T2/FLAIR nonenhancing lesion on stable or increasing doses of corticosteroids compared with baseline scan or best response after initiation of therapy[†] not caused by comorbid events (eg, radiation therapy, demyelination, ischemic injury, infection, seizures, postoperative changes or other treatment effects); any new lesion; clear clinical deterioration not attributable to other causes apart from the tumor (eg, seizures, medication adverse effects, complications of therapy, cerebrovascular events, infection, etc.) or changes in corticosteroid dose;

				failure to return for evaluation as a result of death or deteriorating condition; or clear progression of nonmeasurable disease.

†Progression occurs when this criterion is met.
‡Increase in corticosteroids alone will not be taken into account in determining progression in the absence of persistent clinical deterioration.
Key: ↓: Decrease; ↑: Increase; CR: Complete response; FLAIR: Fluid-attenuated inversion recovery; GD: Gadolinium; HGG: High-grade glioma; NA: Not applicable; PD: Progressive disease; PR: Partial response; T1: T1-Weighted MRI; T2: T2-Weighted MRI
Reproduced from Chukwueke and Wen, 2019, which modified the table with permission from Levin VA, Crafts DC, Norman DM et al. Criteria for evaluating patients undergoing chemotherapy for malignant brain tumors. J. Neurosurg. 47(3), 329–335 (1977).

Detailed analyses of efficacy endpoints are described in **Section 14.4**.

10.3 Response Assessments

Radiographic response assessments will be performed at six months and one year after LITT and/or at the timing of the best radiographic response. If diagnostic imaging results are suggestive of CR, PR, or progression, repeat MRI will be performed 4 to 6 weeks later (**Section 10.1.7**). If pseudo-progression is considered, short-term steroids are allowed, and a follow up MRI must confirm progression after 6 weeks. Lesions that decrease in size with steroids on 6 week follow up MRI will be considered pseudo-progression and will not be considered true progression.

10.4 Time-to-event Endpoints

10.4.1 Definitions

10.4.1.1 **Progression-Free Survival**

Progression-free survival (PFS) is defined as the elapsed time from start date of LITT to first documented evidence of disease progression or death from any cause, whichever is earlier. For surviving patients without progression, follow-up will be censored at the date of last documented progression-free status.

Disease progression is defined as an increase in tumor size of 25% or greater or clinical worsening not amenable to short-term steroid increases at a minimum of 3 months after laser ablation. If diagnostic imaging results are suggestive of CR, PR, or progression, repeat MRI will be performed 4 to 6 weeks later (**Section 10.1.7**). If pseudo-progression is considered, short-term steroids are allowed, and a follow up MRI must confirm progression after 6 weeks. Lesions that decrease in size with steroids on 6 week follow up MRI will be considered pseudo-progression and will not be considered true progression.

10.4.1.2 **Overall Survival**

Overall survival is determined as the elapsed time from start date of LITT to death from any cause. For surviving patients, follow-up will be censored at the last date known to be alive.

10.4.1.3 Duration of Response

Duration of Response (DoR) is defined as the elapsed time from date of the first response (CR or PR) to the date of initial objectively documented progression or death from any cause, whichever is earlier. For patient with neither progression nor death, follow-up will be censored at the date of last tumor evaluation.

10.4.2 Survival Assessment

Participants will be followed up for survival either via telephone or direct visits with the Investigator or study team.

11 ADVERSE EVENTS AND SERIOUS ADVERSE EVENTS

The descriptions and grading scales found in the revised National Cancer Institute Common Terminology Criteria for Adverse Events (NCI CTCAE) version 5.0 will be utilized for adverse event reporting.

11.1 Definition of Adverse Events

Adverse event (AE) means any untoward medical occurrence associated with the use of an intervention in humans, whether or not considered intervention-related (21 CFR 312.32 (a)).

11.1.1 Abnormal Laboratory Values

Not every laboratory abnormality qualifies as an AE. A laboratory test result must be reported as an AE if it meets any of the following criteria:

- Accompanied by clinical symptoms;
- Results in more frequent unscheduled assessments or further diagnostic evaluation not mandated by protocol;
- Results in a change in study treatment (eg, dosage modification, treatment interruption, or treatment discontinuation);
- Results in a medical intervention (eg, potassium supplementation for hypokalemia) or a change in concomitant therapy; or
- Clinically significant in the Investigator's judgment.

It is the Investigator's responsibility to review all laboratory findings. Medical and scientific judgment should be exercised in deciding whether an isolated laboratory abnormality should be classified as an AE.

If a clinically significant laboratory abnormality is a sign of a disease or syndrome (eg, alkaline phosphatase and bilirubin 5 times the ULN associated with cholestasis), only the diagnosis (ie, cholestasis) should be recorded on the Adverse Event CRF/eCRF.

If a clinically significant laboratory abnormality is not a sign of a disease or syndrome, the abnormality itself should be recorded on the Adverse Event CRF/eCRF, along with a descriptor indicating if the test result is above or below the normal range (eg, "elevated potassium," as opposed to "abnormal potassium"). If the laboratory abnormality can be characterized by a precise clinical term per standard definitions, the clinical term should be recorded as the AE. For example, an elevated serum potassium level of 7.0 mEq/L should be recorded as hyperkalemia.

Observations of the same clinically significant laboratory abnormality from visit to visit should not be repeatedly recorded on the Adverse Event CRF/eCRF unless the etiology changes. The initial severity of the event should be recorded, and the severity or seriousness should be updated any time the event worsens (**Section 11.3.4**).

11.2 Definition of Serious Adverse Events

An AE or suspected adverse reaction is considered "serious" if, in the view of either the Sponsor-Investigator or the pharmaceutical company supplying study product, it results in any of the following outcomes:

1. Death;
2. A life-threatening AE;
3. Inpatient hospitalization or prolongation of existing hospitalization*;

4. A persistent or significant incapacity or substantial disruption of the ability to conduct normal life functions;
5. Congenital anomaly/birth defect.

* Participants who are re-admitted to the hospital after surgery if they have seizures or headaches that are unrelated to the intervention will not be considered as experiencing a serious adverse event (SAE). For example, a patient may be hospitalized because they had a seizure or weaning steroids and feel fatigued.

Important medical events that may not result in death, be life-threatening, or require hospitalization may be considered serious when, based upon appropriate medical judgment, they may jeopardize the participant and may require medical or surgical intervention to prevent one of the outcomes listed in this definition. Examples of such medical events include allergic bronchospasm requiring intensive treatment in an emergency room or at home, blood dyscrasias or convulsions that do not result in inpatient hospitalization, or the development of drug dependency or drug abuse.

11.3 Classification of an Adverse Event

11.3.1 Severity of Event

For AEs not included in the protocol-defined grading system, the following guidelines will be used to describe severity.

- **Mild** – Events require minimal or no treatment and do not interfere with the participant's daily activities.
- **Moderate** – Events result in a low level of inconvenience or concern with the therapeutic measures. Moderate events may cause some interference with functioning.
- **Severe** – Events interrupt a participant's usual daily activity and may require systemic drug therapy or other treatment. Severe events are usually potentially life-threatening or incapacitating. Of note, the term "severe" does not necessarily equate to "serious."

11.3.2 Relationship to Study Intervention

All AEs must have their relationship to study intervention assessed by the clinician who examines and evaluates the participant based on temporal relationship and his/her clinical judgment. The degree of certainty about causality will be graded using the categories below. In a clinical trial, the study product must always be suspect.

Definitely Related – There is clear evidence to suggest a causal relationship, and other possible contributing factors can be ruled out. The clinical event, including an abnormal laboratory test result, occurs in a plausible time relationship to study intervention administration and cannot be explained by concurrent disease or other drugs or chemicals. The response to withdrawal of the study intervention (dechallenge) should be clinically plausible. The event must be pharmacologically or phenomenologically definitive, with use of a satisfactory rechallenge procedure if necessary.

Probably Related – There is evidence to suggest a causal relationship, and the influence of other factors is unlikely. The clinical event, including an abnormal laboratory test result, occurs within a reasonable time after administration of the study intervention, is unlikely to be attributed to concurrent disease or other drugs or chemicals, and follows a clinically reasonable response on withdrawal (dechallenge). Rechallenge information is not required to fulfill this definition.

Potentially Related – There is some evidence to suggest a causal relationship (eg, the event occurred within a reasonable time after administration of the trial medication). However, other factors may have contributed to the event (eg, the participant's clinical condition, other concomitant events). Although an AE may rate only as "possibly related" soon after discovery, it can be flagged as requiring more information and later be upgraded to "probably related" or "definitely related," as appropriate.

Unlikely to be Related – A clinical event, including an abnormal laboratory test result, whose temporal relationship to study intervention administration makes a causal relationship improbable (eg, the event did not occur within a reasonable time after administration of the study intervention) and in which other drugs or chemicals or underlying disease provides plausible explanations (eg, the participant's clinical condition, other concomitant treatments).

Not Related – The AE is completely independent of study intervention administration, and/or evidence exists that the event is definitely related to another etiology. There must be an alternative, definitive etiology documented by the clinician.

11.3.3 Expectedness

The Sponsor-Investigator will be responsible for determining whether an AE is expected or unexpected. An AE will be considered unexpected if the nature, severity, or frequency of the event is not consistent with the risk information previously described for the study intervention.

11.3.4 Time Period and Frequency for Event Assessment and Follow-Up

The occurrence of an AE or SAE may come to the attention of study personnel during study visits and interviews of a study participant presenting for medical care or upon review by a study monitor.

All AEs will be captured on the appropriate CRF/eCRF as well as in the Adverse Event reporting section in Velos, a HIPAA AND 21 CFR part 11-compliant database, and will be reported to the University of Miami's IRB per institutional requirements.

Any medical condition that is present at the time that the participant is screened will be considered as baseline and not reported as an AE. However, if the study participant's condition deteriorates at any time during the study, it will be recorded as an AE.

Changes in the severity of an AE will be documented to allow an assessment of the duration of the event at each level of severity to be performed. AEs characterized as intermittent require documentation of onset and duration of each episode.

Study personnel will record all reportable events with start dates occurring any time after informed consent is obtained until 7 (for non-serious AEs) or 30 days (for SAEs) after the last day of study participation or after the end of the DLT monitoring period (**Section 4.5.5**) if the participant withdraws consent for further study participation or meets one or more study discontinuation criteria (**Section 5.2**) during the DLT monitoring period, whichever occurs later. At each study visit, the Investigator will inquire about the occurrence of AE/SAEs since the last visit. Events will be followed for outcome information until resolution or stabilization.

11.4 Adverse Event Reporting

Adverse events (AEs) may be spontaneously identified by the participant and/or in response to an open question from study personnel or revealed by observation, physical examination, or other diagnostic procedures. Any clinically relevant deterioration in laboratory assessments (**Section 11.1.1**) or other

clinical finding is considered an AE. When possible, signs and symptoms indicating a common underlying pathology should be noted as one comprehensive event.

The Sponsor-Investigator is responsible for reporting AEs to any regulatory agency, to the pharmaceutical company supplying study product, and to the Sponsor-Investigator's IRB.

11.5 Serious Adverse Event Reporting

Generally, any AE considered serious by the Principal Investigator or Sub-Investigator or which meets the definition of an SAE included in **Section 11.2, Definition of Serious Adverse Events.**

Serious adverse events (SAEs) will be captured on the appropriate CRF/eCRF as well as in the Serious Adverse Event reporting section in Velos, a HIPAA AND 21 CFR part 11-compliant database, and will be reported to the University of Miami's IRB per institutional requirements.

Serious adverse events (SAEs) will be reported to the pharmaceutical company supplying study product using either a company-provided SAE Report Form Medwatch 3500A Reporting form once the Investigator determines that the event meets the protocol definition of an SAE. The Sponsor-Investigator or designee will report the SAE to the pharmaceutical company supplying study product **within 24 hours of his/her becoming aware of these events regardless of relationship of the SAE to the use of study intervention**.

According to 21 CFR 312.32(c)(1), "the sponsor must notify FDA in an [Investigational New Drug] IND safety report of potential serious risks, from clinical trials or any other source, as soon as possible, but in no case later than 15 calendar days after the sponsor determines that the information qualifies for reporting… In each IND safety report, the sponsor must identify all IND safety reports previously submitted to FDA concerning a similar suspected adverse reaction and must analyze the significance of the suspected adverse reaction in light of previous, similar reports or any other relevant information. The sponsor must report any suspected adverse reaction that is both serious and unexpected. The sponsor must report an adverse event as a suspected adverse reaction only if there is evidence to suggest a causal relationship between the drug and the adverse event, such as:

> (A) A single occurrence of an event that is uncommon and known to be strongly associated with drug exposure (eg, angioedema, hepatic injury, Stevens-Johnson Syndrome);
> (B) One or more occurrences of an event that is not commonly associated with drug exposure, but is otherwise uncommon in the population exposed to the drug (eg, tendon rupture);
> (C) An aggregate analysis of specific events observed in a clinical trial (such as known consequences of the underlying disease or condition under investigation or other events that commonly occur in the study population independent of drug therapy) that indicates those events occur more frequently in the drug treatment group than in a concurrent or historical control group."

Furthermore, according to 21 CFR 312.32(c)(2), "the sponsor must also notify FDA of any unexpected fatal or life-threatening suspected adverse reaction as soon as possible but in no case later than 7 calendar days after the sponsor's initial receipt of the information."

11.6 Reporting Events to Participants

Not Applicable

11.7 Events of Special Interest

Not Applicable

11.8 Reporting of Pregnancy

Every effort should be made to prevent pregnancy throughout the entire duration of participation in this study. All participants of reproductive potential involved in the study are required to use effective methods of contraception during the study according to the contraception requirements in **Section 4.11**.

If, following initiation of the investigational product, it is subsequently discovered that a study participant is pregnant or may have been pregnant at the time of investigational product exposure, pregnant participants will discontinue study medication for the duration of the pregnancy. The Investigator may collect information on pregnancy outcomes for female participants or pregnant female partner(s) of male participants.

Follow-up information regarding the course of the pregnancy, including perinatal and neonatal outcome and, where applicable, offspring information may be reported on the CIOMS, MedWatch, Surveillance Form, **or** approved site SAE form.

Pregnancy events for enrolled participants will be reported to the pharmaceutical company supplying study product by email or by fax as soon as possible but not later than (3) business days following receipt of notice of such occurrence.

12 UNANTICIPATED PROBLEMS

12.1 Definition of Unanticipated Problems

The Office for Human Research Protections (OHRP) considers unanticipated problems involving risks to participants or others to include, in general, any incident, experience, or outcome that meets **all** of the following criteria:

- Unexpected in terms of nature, severity, or frequency given (a) the research procedures that are described in the protocol-related documents, such as the IRB-approved research protocol and informed consent document; and (b) the characteristics of the participant population being studied;
- Related or possibly related to participation in the research ("possibly related" means there is a reasonable possibility that the incident, experience, or outcome may have been caused by the procedures involved in the research); and
- Suggests that the research places participants or others at a greater risk of harm (including physical, psychological, economic, or social harm) than was previously known or recognized.

12.2 Unanticipated Problem Reporting

The Sponsor-Investigator will report unanticipated problems (UPs) to the reviewing IRB.

12.3 Reporting Unanticipated Problems to Participants

Not Applicable

13 DATA REPORTING

13.1 Data Submission

Electronic case report forms (eCRFs) for study data entry will be developed by staff at the University of Miami using Velos, a HIPAA AND 21 CFR part 11-compliant database. Only Investigators and assigned research staff will have access to study data. The eCRFs will be available to Sponsor, IRB, or regulatory authorities in event of an audit.

13.2 Data and Safety Monitoring

All clinical studies conducted at the University of Miami (UM) Sylvester Comprehensive Cancer Center (SCCC) must include provisions for data and safety monitoring. The University of Miami Sylvester Comprehensive Cancer Center Data and Safety Monitoring Committee (DSMC) evaluates all Investigator-initiated studies to ensure participant safety monitoring is commensurate with risk.

The DSMC is responsible for:

- Protecting participant safety
- Ensuring the credibility and integrity of the data
- Providing analysis of efficacy data and interim reviews of safety monitoring

The DSMC is composed of experts in relevant scientific disciplines to ensure critical data and safety review of all cancer related institutional, interventional studies. The committee comprises medical oncologists, scientists, nurses, clinical research coordinators, regulatory analysts, a statistician, and a pharmacist.

14 STATISTICAL CONSIDERATIONS

14.1 Overview

This study is a single institution, single-arm, phase 1 study to determine the RP2D of ritonavir as part of the ART regimen in patients with newly-diagnosed, unresectable HGG post LITT. Participants will also receive standard of care adjuvant therapy (temozolomide + radiotherapy).

For dose escalation/de-escalation of ritonavir, a traditional 3+3 design will be performed for testing four planned dose levels: 100, 300 (starting dose), 400, and 600 mg two times per day (**Table 3**). Dose expansion will be conducted to confirm safety and tolerability of the determined RP2D from the dose-finding part and to assess the preliminary efficacy of the treatment (**Section 4.5.2.1**).

14.2 Sample Size Justification

We will enroll a total of 24 eligible participants in this study. Based on our 3+3 design in part 1, the number of participants for dose escalation/de-escalation is between 12–18 (if only dose levels 2, 3, and 4 are tested) and maximum 24 (if all four dose levels are tested with 6 participants per level). Thus, the expected total number of participants in part 1 is 12–24. An additional 6–18 participants including 6 participants treated with the RP2D in part 1 will be enrolled in dose-expansion cohort to be treated at the RP2D of ritonavir.

14.3 Definitions and Populations for Analyses

On study: A participant is considered to be *on study* at the time an informed consent is signed.

On treatment: Participants are considered to be *on treatment* as long as they continue to receive study treatment. Participants who are *off treatment* will remain on study and will be followed for toxicity, progression, and survival.

Per-protocol population: All evaluable participants for safety and/or efficacy endpoints.

Safety population: All **evaluable participants for safety.** Study-eligible participants who initiate the study treatment and received at least **one dose** of ritonavir, regardless of treatment being completed. All evaluable participants for safety will be assessed for toxicity.

Efficacy population (Per-protocol Analysis Dataset): All **evaluable participants for efficacy.** Study-eligible participants who initiate the study treatment and received at least **one dose** of ritonavir, regardless of treatment being completed. All evaluable participants for efficacy will be assessed for treatment response at the time periods as described in **Section 10.3**, end of study, and/or disease progression as well as for survival. All evaluable participants for efficacy will be under follow-up until they withdraw consent for further follow-up (**Section 5.2**) or die.

- **Not evaluable participants (Exclusions from study analysis):**
    - Participants who are enrolled on study but not treated (do not receive any dose of ritonavir) will be excluded from all analyses. Reasons for such withdrawals, such as eligibility not confirmed or consent withdrawn, will be characterized.
    - Any participant who is treated but later found to be ineligible for study (a “protocol violation”) will be withdrawn from study but followed for progression, survival and toxicity. Such participant experience will be characterized separately from that of evaluable participants.

14.4 Statistical Analyses

14.4.1 Baseline Descriptive Statistics

Baseline patient characteristics will be summarized using descriptive statistics. Counts and percentages will be used to summarize the distribution of categorical variables. Median, range, mean, and standard deviation will be used for continuous variables.

14.4.2 Analysis of the Primary Endpoint

Treatment dose level cohorts will be summarized reporting the number of patients treated, the number who experience DLT, SAEs and grade 3 or higher AE, the number of patients who discontinue therapy, and the reasons for discontinuation. Comprehensive safety data on all toxicities will be tabulated by type, grade, duration, attribution to treatment, and dose level received. A patient level summary by worst grade toxicity will be included. Toxicity will be assessed by the PI and assigned severity and attribution using NCI CTCAE version 5.0.

14.4.3 Analysis of the Secondary and Exploratory Endpoint(s)

Secondary and exploratory efficacy endpoints include ORR (defined as proportion of patients achieving CR or PR after LITT + ART as determined by the RANO criteria (**Section 10.4.1.2**)), PFS (**Section 10.4.1.1**), OS (**Section 10.4.1.2**), and DoR (**Section 10.4.1.3**). ORR will be summarized using point estimates and corresponding 95% confidence interval (CI) using the Clopper-Pearson exact method. PFS, OS and DoR will be analyzed using Kaplan-Meier (KM) method. When KM method is used, point estimates and 2-sided 95% confidence intervals will be reported for selected times using Greenwood's variance and the log-log transform method. Median PFS, OS and DoR will also be reported if attained.

14.4.4 Planned Interim Analyses

Interim analyses are not planned for this study.

14.5 Early Stopping Guidelines

14.5.1 Early Stopping Due to Safety

We expect the number of participants in the expansion cohort to be 6–18 participants. We propose the following guidelines for DSMC to consider in its review of accumulating study data. The proposed guidelines were developed using Bayesian methods, which can be applied at any stage of enrollment without advance specification of the number of interim analyses to be performed or the number of evaluable participants at the time such assessments are made. Under the Bayesian method, we assign a prior probability (level of belief at the start of the trial) to a range of possible values for the true toxicity rate. As toxicity data on study participants become available, the prior probability distribution is revised, and the resulting posterior probability distribution becomes the basis for recommending either early termination or continuation of the study.

The following early stopping guidelines in **Table 7**, which are based on a Bayesian method, will be applied to ensure safety of this trial. Safety monitoring will be based on the occurrence of treatment related (possible, probable, or definite) grade 3 or higher toxicity or DLT (**Section 4.5.5**) occurring during ART treatment. We suggest as a guideline for decreasing the dose to previous level a posterior probability of 80% or higher that the rate of grade 3 or higher toxicity exceeds 15%. **Table 7** shows specific instances where this guideline is met.

Table 7. Early Stopping Rules for Safety in the Dose Expansion Cohort

Number of participants with treatment related grade 3+ toxicity*	Total participants evaluated	Observed rate ≥
2	3 to 6	33%
3	7 to 11	27%
4	12 to 16	25%
5	17 to 18	28%
* Possible, probable, or definite treatment-related toxicity		

To illustrate the stopping guidelines, suppose that 6 evaluable participants have been assessed for toxicity and 2 of them have experienced grade 3 treatment-related toxicity (the first row in **Table 7**). Under this circumstance, the observed rate of grade 3+ toxicity is 33.3%, resulting in a posterior probability of 80.1% that the true underlying rate exceeds 15%, thereby suggesting early termination. Posterior probabilities used to derive guidelines for toxicity are calculated under a weak prior beta distribution with parameters $\boldsymbol{\beta_1}$=0.3 and $\boldsymbol{\beta_2}$=1.7, which corresponds to an expected unacceptable toxicity rate of 15% based on prior information roughly equal to having studied 2 participants. This prior distribution assigns a small a priori chance of only 32.3% that the true rate of unacceptable toxicity is 15% or greater.

14.5.2 Early Stopping Due to a Lack of Efficacy

Not planned due to small sample size in dose expansion cohort.

15 SUPPORTING DOCUMENTATION AND OPERATIONAL CONSIDERATIONS

15.1 Informed Consent Process

15.1.1 Consent/Assent and other Informational Documents Provided to Participants

Consent forms describing in detail the study intervention, study procedures, and risks are given to the participant and written documentation of informed consent is required prior to starting intervention/administering study intervention.

15.1.2 Consent Procedures and Documentation

Informed consent is a process that is initiated prior to the individual's agreeing to participate in the study and continues throughout the individual's study participation. Consent forms will be IRB-approved, and the participant will be asked to read and review the document. We anticipate enrolling non-English speaking participants. Consent forms will be translated to applicable languages for the participant population in the trial site. The Investigator will explain the research study to the participant and answer any questions that may arise. A verbal explanation will be provided in terms suited to the participant's comprehension of the purposes, procedures, and potential risks of the study and of their rights as research participants. The consent process will take place in a private clinic room. Participants will be given as much time as needed for them to be comfortable with participating in this study. Participants will have the opportunity to carefully review the written consent form and ask questions prior to signing. The participants should have the opportunity to discuss the study with their family or surrogates or think about it prior to agreeing to participate. The participant will sign and date the informed consent document prior to any procedures being done specifically for the study. Participants must be informed that participation is voluntary and that they may withdraw from the study at any time without prejudice. A copy of the informed consent document will be given to the participants for their records. The informed consent process will be conducted and documented in the source document (including the date) and the form signed before the participant undergoes any study-specific procedures. The rights and welfare of the participants will be protected by emphasizing to them that the quality of their medical care will not be adversely affected if they decline to participate in this study or leave this study early.

The Investigator or qualified personnel from the research team will determine if participants have the capacity to provide legal consent.

15.2 Study Discontinuation and Closure

This study may be temporarily suspended or prematurely terminated if there is sufficient reasonable cause. Written notification, documenting the reason for study suspension or termination, will be provided by the suspending or terminating party to study participants, Investigators, funding agency, the IND Sponsor, and regulatory authorities. If the study is prematurely terminated or suspended, the Sponsor-Investigator will promptly inform study participants, the IRB, and the pharmaceutical company supplying study product and will provide the reason(s) for the termination or suspension. Study participants will be contacted, as applicable, and be informed of changes to the study visit schedule.

Circumstances that may warrant termination or suspension include but are not limited to:

- Determination of unexpected, significant, or unacceptable risk to participants
- Demonstration of efficacy that would warrant stopping

- Insufficient compliance to protocol requirements
- Data that are not sufficiently complete and/or evaluable
- Determination that the primary endpoint has been met
- Determination of futility

Study may resume once concerns about safety, protocol compliance, and data quality are addressed and satisfy the IRB and/or FDA.

15.3 Confidentiality and Privacy

Research participants will sign a consent form and HIPAA authorization before data is collected as part of this study. Data collected as part of this trial includes PHI or personally identifiable information and data from the electronic medical record. Study personnel will access electronic medical record or other PHI without obtaining a signed HIPAA authorization from the patient to identify potential participants for recruitment.

The Principal Investigator (and/or Study Team members) will record (eg, write down, abstract) the data collected in a manner that does not include any direct identifiers of any participant. Instead, the Principal Investigator and/or Study Team members will assign a code (that is not derived in whole or in part from any direct or indirect identifiers of the individual) to each study participant and link the code to the study participant's identity. The link to each participant's identity and/or other identifiable information will be maintained on a document separate from the research data.

Information collected as part of this study will be destroyed or de-identified at the earliest opportunity. Information collected will not be reused or disclosed to any other person or entity, except as required by law, for authorized oversight of the research study or for other research for which the use or disclosure of PHI is permissible.

Research data may be sent to the pharmaceutical company supplying study product**.** Data sent will be coded/de-identified. Information may be sent via email, fax, FedEx, UPS, USPS, courier, or via a study-specific electronic data capture system. Procedures to protect confidentiality of information being sent are the following: de-identifying/coding reports, utilizing cover sheets for faxes, sending emails via secure transmittal, password-protected files, and utilizing delivery confirmation/tracking for items shipped via courier.

Specimens obtained for this research will be de-identified and coded with the link between code and the participant's identity maintained separately from the data. Specimens will not be sent to external entities.

Participant confidentiality and privacy is strictly held in trust by the Sponsor-Investigator, participating Investigators, and their staff. This confidentiality is extended to cover testing of biological samples and genetic tests in addition to the clinical information relating to participants. Therefore, the study protocol, documentation, data, and all other information generated will be held in strict confidence. No information concerning the study or the data will be released to any unauthorized third party without prior written approval of the Sponsor-Investigator.

All research activities will be conducted in as private a setting as possible.

The study monitor, other authorized representatives of the Sponsor-Investigator, representatives of the IRB, regulatory agencies, or pharmaceutical company supplying study product may inspect all documents and records required to be maintained by the Investigator, including but not limited to

medical records (office, clinic, or hospital) and pharmacy records for the participants in this study. The Sponsor-Investigator will permit access to such records.

The study participant's contact information will be securely stored in the University of Miami's password-protected electronic devices and University of Miami approved cloud-based storage systems for internal use during the study. At the end of the study, all records will continue to be kept in a secure location for as long a period as dictated by the reviewing IRB, Institutional policies, or pharmaceutical company supplying study product requirements.

Electronic case report forms (eCRFs) for study data entry will be developed by staff at the University of Miami using Velos, a HIPAA AND 21 CFR part 11-compliant database. Only Investigators and assigned research staff will have access to study data.

15.4 Future Use of Stored Specimens and Data

Data collected for this study will be analyzed and stored at the University of Miami on Velos.

With the participant's approval and as approved by local IRBs, biological samples will be stored at the University of Miami per institutional regulations. These samples could be used to research the causes of the condition being studied as part of this protocol or may be stored indefinitely for research as new knowledge or technology becomes available. The laboratory storing the biological specimens will also be provided with a code-link that will allow linking the biological specimens with the phenotypic data from each participant, maintaining the blinding of the identity of the participant. The Principal Investigator and other designated study staff will have access to the code sheet including Sub-Investigators, clinical coordinators, data coordinators, clinical manager, and regulatory staff.

During the conduct of the study, an individual participant can choose to withdraw consent to have biological specimens stored for future research. However, withdrawal of consent with regard to biospecimen storage may not be possible after the study is completed.

When the study is completed, access to study data and/or samples will be provided through Velos and the laboratory storing the biological specimens.

15.5 Study Auditing and Monitoring

This study will be monitored (as applicable) and may be audited according to the University of Miami requirements. See also:
http://research.med.miami.edu/clinical-research/crors/monitoring

Following the monitoring plan, the monitors will verify that the clinical trial is conducted, data are generated, and biological specimens are collected, documented (recorded), and reported in compliance with the protocol, International Conference on Harmonisation Good Clinical Practice (ICH GCP), and applicable regulatory requirements.

15.5.1 Trial Monitoring, Auditing, and Inspecting

The Investigator will permit trial-related monitoring, quality audits, and inspections by government regulatory authorities of all trial-related documents (eg, source documents, regulatory documents, data collection instruments, CRFs/eCRFs). The Investigator will ensure the capability for inspections of applicable trial-related facilities. The Investigator will ensure that the trial monitor or any other compliance or Quality Assurance (QA) reviewer is given access to all trial-related documents and trial-related facilities.

Participation as an Investigator in this trial implies the acceptance of potential inspection by government regulatory authorities.

15.6 Quality Assurance and Quality Control

In addition to the Clinical Monitoring component of this protocol, QA will be implemented to assess compliance with Good Clinical Practice (GCP) and applicable regulatory requirements. Data or documentation audited shall be assessed for compliance to the protocol, accuracy in relation to source documents, and compliance to applicable regulations.

15.7 Data Handling and Record Keeping

15.7.1 Data Collection and Management Responsibilities

Data collection is the responsibility of the clinical trial staff at the site under the supervision of the Sponsor-Investigator. The Sponsor-Investigator is responsible for ensuring the accuracy, completeness, legibility, and timeliness of the data reported.

All source documents should be completed in a neat, legible manner to ensure accurate interpretation of data.

Data for CRFs/eCRFs will be entered into Velos. A CRF/eCRF is required for every participant who received any amount of study treatment. The Investigator will ensure that the CRFs/eCRFs are accurate, complete, legible and timely. Participants for whom documentation is inadequate to determine eligibility will generally be deemed ineligible. Data recorded in the CRF/eCRF derived from source documents should be consistent with the data recorded on the source documents.

Clinical data (including AEs, concomitant medications, and expected adverse reactions data) and clinical laboratory data will be entered into Velos, a HIPAA AND 21 CFR part 11-compliant data capture system provided by the University of Miami. The data system includes password protection and internal quality checks, such as automatic range checks, to identify data that appear inconsistent, incomplete, or inaccurate. Clinical data will be entered directly from the source documents.

15.7.2 Study Records Retention

All records and documents relating to research studies and participants must be kept confidential to the extent permitted by law; however, records and documents shall be available in a timely manner to the University authorized employees or other agents authorized by the University including IRB members and Human Subjects Research Office (HSRO) staff and appropriate governmental agencies including but not limited to the Department of Health and Human Services (DHHS), OHRP, and the FDA.

Although Principal Investigators are responsible for the creation and maintenance of research records and documents, such records and documents (including data collected pursuant to research) are the property of the University. Until the temporal requirements for record/document retention are met, Investigators or others may not remove or destroy research records or documents (or copies of such records or documents) without written permission from the Vice Provost of Research. This permission requirement extends to Investigators leaving the University even if they plan to continue the research at another institution.

With certain exceptions, Investigators must retain complete records and documents (including the consent documents) from their study for the duration of that study and for a minimum period of three (3) years following closure of a study. Exceptions to this 3-year minimum retention period are:

a. HIPAA REQUIREMENTS: if a study involves the collection of identifiable health information, records must be retained for a minimum of six (6) years following study closure. This retention period is consistent with the HIPAA Privacy Rule under which subjects [participants] may ask Investigators for an accounting of all uses and disclosures of their study information for a period of 6 years after their participation is completed (c.f. 45 CFR 164.528)
b. FDA REQUIREMENTS FOR A STUDY INVOLVING AN INVESTIGATIONAL DRUG UNDER AN IND (c.f. 21 CFR 312.62): if a study involves the use of an investigational drug under an IND, Principal Investigators must retain study records and documents until at least the later of the following dates:
    a. Two (2) years following the date of a marketing application is approved for the drug for the indication for which it was being investigated; or
    b. Two (2) years after the investigation is discontinued and the FDA is notified if no marketing application is to be filed or, if the application is not approved for such indication; or
    c. Three (3) years after IRB approval of the closure of the study
c. FDA REQUIREMENTS FOR A STUDY INVOLVING AN INVESTIGATIONAL DEVICE UNDER AN IDE (c.f. 21 CFR 812.140): if a study involves an investigational device under an IDE [Investigational Device Exemption], Principal Investigators must retain study records and documents until at least the later of the following dates:
    a. Two (2) years following the date on which the investigation is terminated or completed; or
    b. Two (2) years following the date that the records are no longer required for purposes of supporting a premarket approval application or a notice of completion of a product development protocol; or
    c. Three (3) years after IRB approval of the closure of the study.

NOTE–The FDA two-year requirements may occur during the applicable retention period, or it may occur afterward and be additional to that period.

d. VA REQUIREMENTS: if a study engages the VA [Veterans Affairs], Investigators must retain research records and documents for a minimum of five (5) years after IRB approval of study closure. This retention period is consistent with the VA's Records Control Schedule (RCS 10-1).
e. ICH-GCP REQUIREMENTS: trial documents must be retained as specified in Essential Documents for the Conduct of a Clinical Trial and as required by the applicable regulatory requirement(s). Measures must be taken to prevent accidental or premature destruction of these documents.

Records must be retained longer than the times specified above as other requirements may apply, such as may be forthcoming from sponsors in executed contracts, institutional entities, or extramural funding agencies.

If your Human Research is funded by a pharmaceutical company supplying study product, contact the pharmaceutical company before disposing of Human Research records. The protocol and clinical trial agreement/contract will also contain terms for records retention.

15.8 Compliance with Protocol

The Investigator/institution should conduct the trial in compliance with the protocol agreed to by the pharmaceutical company supplying study product and, if required, by the regulatory authorities and which was given approval opinion by the IRB.

The Investigator should not implement any deviation from or changes of the protocol without agreement by the pharmaceutical company supplying study product and prior review and documented approval opinion from the IRB of an amendment, except where necessary to eliminate an immediate hazard to trial participants, or when the changes involve only logistical or administrative aspects of the trial (eg, change in monitors, change of telephone numbers).

The Investigator or designee should document and explain any deviation from the approved protocol.

The Investigator may implement a deviation from or change of the protocol to eliminate an immediate hazard to trial participants without prior IRB approval opinion. As soon as possible, the implemented deviation or change, the reasons for it, and, if appropriate, the proposed protocol amendments should be submitted:

a. To the IRB for review and approval opinion;
b. To the pharmaceutical company supplying study product for agreement, if required;
c. To the regulatory authorities.

15.9 Publication and Data Sharing

All information provided regarding the trial as well as all information collected/documented during the course of the trial will be regarded as confidential.

The financial disclosure information will be completed prior to trial participation from all Principal Investigators and Sub-Investigators who are involved in the trial and named on the FDA 1572 form.

The Sponsor-Investigator or designee will register the trial on www.clinicaltrials.gov. In addition, Sponsor-Investigator will publish the results of the trial.

15.10 Conflict of Interest Policy

The independence of this study from any actual or perceived influence, such as by the pharmaceutical industry, is critical. Therefore, any actual conflict of interest of persons who have a role in the design, conduct, analysis, publication, or any aspect of this trial will be disclosed and managed. Furthermore, persons who have a perceived conflict of interest will be required to have such conflicts managed in a way that is appropriate to their participation in the design and conduct of this trial. The study leadership in conjunction with the University of Miami has established policies and procedures for all study group members to disclose all conflicts of interest and will establish a mechanism for the management of all reported dualities of interest.

16 REFERENCES

1. Chow WA, Jiang C, Guan M. Anti-HIV drugs for cancer therapeutics: back to the future? Lancet Oncol. 2009 Jan;10(1):61-71. doi: 10.1016/S1470-2045(08)70334-6. PMID: 19111246.
2. Coffey RJ, Lunsford LD, Taylor FH: Survival after stereotactic biopsy of malignant gliomas . Neurosurgery. 1988 Mar;22(3):465–73. doi: 10.1227/00006123-198803000-00003. PMID: 2452376.
3. de Groot JF, Kim AH, Prabhu S, Rao G, Laxton AW, Fecci PE, O'Brien BJ, Sloan A, Chiang V, Tatter SB, Mohammadi AM, Placantonakis DG, Strowd RE, Chen C, Hadjipanayis C, Khasraw M, Sun D, Piccioni D, Sinicrope KD, Campian JL, Kurz SC, Williams B, Smith K, Tovar-Spinoza Z, Leuthardt EC. Efficacy of laser interstitial thermal therapy (LITT) for newly diagnosed and recurrent IDH wild-type glioblastoma. Neurooncol Adv. 2022 Apr 6;4(1):vdac040. doi: 10.1093/noajnl/vdac040. PMID: 35611270; PMCID: PMC9122789.
4. Fazeny-Dörner B, Wenzel C, Veitl M, Piribauer M, Rössler K, Dieckmann K, Ungersböck K, Marosi C. Survival and prognostic factors of patients with unresectable glioblastoma multiforme. Anticancer Drugs. 2003 Apr;14(4):305-12. doi: 10.1097/00001813-200304000-00008. PMID: 12679735.
5. Figueroa JM, Semonche A, Magoon S, Shah A, Luther E, Eichberg D, Komotar R, Ivan ME. The role of neutrophil-to-lymphocyte ratio in predicting overall survival in patients undergoing laser interstitial thermal therapy for glioblastoma. J Clin Neurosci. 2020 Feb;72:108-113. doi: 10.1016/j.jocn.2019.12.057. Epub 2020 Jan 7. PMID: 31918907.
6. Khansur E, Shah AH, Lacy K, Komotar RJ. Novel Immunotherapeutics for Treatment of Glioblastoma: The Last Decade of Research. Cancer Invest. 2019;37(1):1-7. doi: 10.1080/07357907.2018.1479414. Epub 2019 Jan 11. PMID: 30632816.
7. Leao DJ, Craig PG, Godoy LF, Leite CC, Policeni B. Response Assessment in Neuro-Oncology Criteria for Gliomas: Practical Approach Using Conventional and Advanced Techniques. AJNR Am J Neuroradiol. 2020 Jan;41(1):10-20. doi: 10.3174/ajnr.A6358. Epub 2019 Dec 19. PMID: 31857322; PMCID: PMC6975322.
8. Leuthardt EC, Duan C, Kim MJ, Campian JL, Kim AH, Miller-Thomas MM, Shimony JS, Tran DD. Hyperthermic Laser Ablation of Recurrent Glioblastoma Leads to Temporary Disruption of the Peritumoral Blood Brain Barrier. PLoS One. 2016 Feb 24;11(2):e0148613. doi: 10.1371/journal.pone.0148613. PMID: 26910903; PMCID: PMC4766093.
9. Ma X, Lv Y, Liu J, Wang D, Huang Q, Wang X, Li G, Xu S, Li X. Survival analysis of 205 patients with glioblastoma multiforme: clinical characteristics, treatment and prognosis in China. J Clin Neurosci. 2009 Dec;16(12):1595-8. doi: 10.1016/j.jocn.2009.02.036. Epub 2009 Sep 29. PMID: 19793663.
10. Mendez Valdez MJ, Lu VM, Kim E, Rivas SR, Govindarajan V, Ivan M, Komotar R, Nath A, Heiss JD, Shah AH. Glioblastoma multiforme in patients with human immunodeficiency virus: an integrated review and analysis. J Neurooncol. 2022 Sep;159(3):571-579. doi: 10.1007/s11060-022-04095-4. Epub 2022 Jul 20. PMID: 35857248.
11. Rauschenbach L, Wieland A, Reinartz R, Kebir S, Till A, Darkwah Oppong M, Dobersalske C, Ullrich V, Ahmad A, Jabbarli R, Pierscianek D, Fröhlich H, Simon M, Brüstle O, Sure U, Glas M, Scheffler B. Drug repositioning of antiretroviral ritonavir for combinatorial therapy in glioblastoma. Eur J Cancer. 2020 Nov;140:130-139. doi: 10.1016/j.ejca.2020.09.017. Epub 2020 Oct 19. PMID: 33091717.
12. Reardon DA, Mitchell DA. The development of dendritic cell vaccine-based immunotherapies for glioblastoma. Semin Immunopathol. 2017 Feb;39(2):225-239. doi: 10.1007/s00281-016-0616-7. Epub 2017 Jan 30. PMID: 28138787.
13. Shah AH, Burks JD, Buttrick SS, Debs L, Ivan ME, Komotar RJ. Laser Interstitial Thermal Therapy as a Primary Treatment for Deep Inaccessible Gliomas. Neurosurgery 2019 Mar 1;84(3):768-777. doi: 10.1093/neuros/nyy238. PMID: 29873756.

14. Shah AH, Semonche A, Eichberg DG, Borowy V, Luther E, Sarkiss CA, Morell A, Mahavadi AK, Ivan ME, Komotar RJ. The Role of Laser Interstitial Thermal Therapy in Surgical Neuro-Oncology: Series of 100 Consecutive Patients. Neurosurgery. 2020 Aug 1;87(2):266-275. doi: 10.1093/neuros/nyz424. PMID: 31742351.
15. Shah AH, Gilbert M, Ivan ME, Komotar RJ, Heiss J, Nath A. The role of human endogenous retroviruses in gliomas: from etiological perspectives and therapeutic implications. Neuro Oncol. 2021 Oct 1;23(10):1647-1655. doi: 10.1093/neuonc/noab142. PMID: 34120190; PMCID: PMC8485438.
16. Shin DH, Melnick KF, Tran DD, Ghiaseddin AP. In situ vaccination with laser interstitial thermal therapy augments immunotherapy in malignant gliomas. J Neurooncol. 2021 Jan;151(1):85-92. doi: 10.1007/s11060-020-03557-x. PMID: 32757094.
17. Srinivasan ES, Sankey EW, Grabowski MM, Chongsathidkiet P, Fecci PE. The intersection between immunotherapy and laser interstitial thermal therapy: a multipronged future of neuro-oncology. Int J Hyperthermia. 2020 Jul;37(2):27-34. doi: 10.1080/02656736.2020.1746413. PMID: 32672126.
18. Stupp R, Mason WP, van den Bent MJ, Weller M, Fisher B, Taphoorn MJ, Belanger K, Brandes AA, Marosi C, Bogdahn U, Curschmann J, Janzer RC, Ludwin SK, Gorlia T, Allgeier A, Lacombe D, Cairncross JG, Eisenhauer E, Mirimanoff RO; European Organisation for Research and Treatment of Cancer Brain Tumor and Radiotherapy Groups; National Cancer Institute of Canada Clinical Trials Group. Radiotherapy plus concomitant and adjuvant temozolomide for glioblastoma. N Engl J Med. 2005 Mar 10;352(10):987-96. doi: 10.1056/NEJMoa043330. PMID: 15758009.
19. Stupp R, Hegi ME, Mason WP, van den Bent MJ, Taphoorn MJ, Janzer RC, Ludwin SK, Allgeier A, Fisher B, Belanger K, Hau P, Brandes AA, Gijtenbeek J, Marosi C, Vecht CJ, Mokhtari K, Wesseling P, Villa S, Eisenhauer E, Gorlia T, Weller M, Lacombe D, Cairncross JG, Mirimanoff RO; European Organisation for Research and Treatment of Cancer Brain Tumour and Radiation Oncology Groups; National Cancer Institute of Canada Clinical Trials Group. Effects of radiotherapy with concomitant and adjuvant temozolomide versus radiotherapy alone on survival in glioblastoma in a randomised phase III study: 5-year analysis of the EORTC-NCIC trial. Lancet Oncol. 2009 May;10(5):459-66. doi: 10.1016/S1470-2045(09)70025-7. Epub 2009 Mar 9. PMID: 19269895.
20. Vogl TJ, Wissniowski TT, Naguib NN, Hammerstingl RM, Mack MG, Münch S, Ocker M, Strobel D, Hahn EG, Hänsler J. Activation of tumor-specific T lymphocytes after laser-induced thermotherapy in patients with colorectal liver metastases. Cancer Immunol Immunother. 2009 Oct;58(10):1557-63. doi: 10.1007/s00262-009-0663-1. Epub 2009 Jan 29. PMID: 19184001.
21. Wang WJ, Mao LF, Lai HL, Wang YW, Jiang ZB, Li W, Huang JM, Xie YJ, Xu C, Liu P, Li YM, Leung ELH, Yao XJ. Dolutegravir derivative inhibits proliferation and induces apoptosis of non-small cell lung cancer cells via calcium signaling pathway. Pharmacol Res. 2020 Nov;161:105129. doi: 10.1016/j.phrs.2020.105129. Epub 2020 Aug 9. PMID: 32783976.

APPENDIX A: PERFORMANCE STATUS SCALES

PERFORMANCE STATUS CRITERIA					
**ECOG (Zubrod)**		**Karnofsky**		**Lansky**	
Score	Description	Score	Description	Score	Description
0	Fully active, able to carry on all pre-disease performance without restriction.	100	Normal, no complaints, no evidence of disease.	100	Fully active, normal.
		90	Able to carry on normal activity, minor signs or symptoms of disease.	90	Minor restrictions in physically strenuous activity.
1	Restricted in physically strenuous activity but ambulatory and able to carry out work of a light or sedentary nature, e.g., light housework, office work.	80	Normal activity with effort, some signs or symptoms of disease.	80	Active, but tires more quickly.
		70	Cares for self, unable to carry on normal activity or do active work.	70	Both greater restriction of, and less time spent in, play activity.
2	Ambulatory and capable of all selfcare but unable to carry out any work activities. Up and about more than 50% of waking hours.	60	Requires occasional assistance, but is able to care for most of his/her needs.	60	Up and around, but minimal active play; keeps busy with quieter activities.
		50	Requires considerable assistance and frequent medical care.	50	Gets dressed, but lies around much of the day; no active play; able to participate in all quiet play and activities.
3	Capable of only limited selfcare, confined to bed or chair more than 50% of waking hours.	40	Disabled, requires special care and assistance.	40	Mostly in bed, participates in quiet activities.
		30	Severely disabled, hospitalization indicated. Death not imminent.	30	In bed, needs assistance even for quiet play.
4	Completely disabled. Cannot carry on any selfcare. Totally confined to a bed or chair.	20	Very sick, hospitalization indicated. Death not imminent.	20	Often sleeping, play entirely limited to very passive activities.
		10	Moribund, fatal processes progressing rapidly.	10	No play, does not get out of bed.
5	Dead	0	Dead	0	Dead

As published in Am J Clin Oncol: Oken MM, Creech RH, Tormey DC, Horton J, Davis TE, McFadden ET, Carbone PP. Toxicity and Response Criteria of the Eastern Cooperative Oncology Group. Am J Clin Oncol 1982;5:649-655. The Eastern Cooperative Oncology Group, Robert Comis, MD, Group Chair.
